# Host–virus interactions dominate fitness and conceal additive genetic variation

**DOI:** 10.1371/journal.pbio.3003836

**Published:** 2026-10-22

**Authors:** Aleksei Belyi, Yiyang Du, Alastair J. Wilson, Ben Longdon, Francis M. Jiggins

**Affiliations:** 1 Department of Genetics, University of Cambridge, Cambridge, United Kingdom; 2 Centre for Ecology and Conservation, University of Exeter, Penryn, United Kingdom; Fred Hutchinson Cancer Research Center, UNITED STATES OF AMERICA

## Abstract

Host–parasite coevolution is expected to generate strong selection for resistance and infectivity that has the potential to erode genetic variation. Despite this, natural populations often retain extensive genetic variation in these traits. Genotype-by-genotype interactions on fitness can generate negative frequency-dependent selection, which helps maintain this variation by preventing any host or pathogen genotype from retaining a permanent advantage. Because these interactions place genetic variance in the interaction term rather than in additive differences among hosts or pathogens, that variance is masked from directional selection and can remain ‘cryptic’. Here we combine coevolutionary modelling with large‑scale experimental infection assays to quantify how host-pathogen interactions structure fitness variance in a vertically transmitted virus system, *Drosophila melanogaster* and sigma virus. Simulations show that coevolution typically erodes additive genetic variance in host and pathogen fitness, concentrating variance in host-virus interaction terms. Consistent with these predictions, experiments spanning 90 host-virus genotype combinations reveal that transmission, viral load, and virulence are overwhelmingly governed by host-virus genetic interactions rather than host or virus main effects. As a result, neither host resistance alleles nor viral genotypes confer consistently higher fitness across genetic backgrounds. Interactions tend to be sex‑specific, further limiting heritable fitness variation. Our results demonstrate that coevolution can substantially mask heritable genetic variation from directional selection by rendering fitness context-dependent. This extensive cryptic genetic variation may be revealed either when ecological or evolutionary conditions shift, or when the process of coevolution itself alters the direction of selection. This demonstrates the need to account for genotype-specific interactions when forecasting evolutionary responses.

## Abstract 2

Host–parasite coevolution has the potential to erode genetic variation, but natural populations often retain extensive genetic variation in susceptibility and infectivity. This study of Drosophila melanogaster and sigma virus shows that coevolution erodes additive genetic variance in host and pathogen fitness, concentrating variance in host–virus interaction terms.

## Introduction

Adaptation by natural selection depends not simply on how much genetic variation a population contains, but on how reliably that variation predicts fitness. Directional selection responses require additive genetic variance which results in differences in mean fitness being predictably passed from parents to offspring. However, if the relative fitness of a genotype depends on the environment it experiences, genotype-by-environment interactions (G × E) can reduce this predictability: a parental genotype that thrives in one setting may fare poorly when expressed by offspring in another. Therefore, there can be abundant genetic variation in phenotype, but little additive variance in fitness. Because selection can act only on consistent differences in mean fitness, such interactions can sometimes slow or even stall the rate of adaptation, even in populations that are genetically variable [1,2]. A biologically important case arises when the relevant ‘environment’ is actually the genotype of another organism [3]. In interactions between males and females [4], competitors, or hosts and their parasites and pathogens (hereafter parasite), the fitness of each partner can depend directly on the genotype of the other [5–7]. This raises the potential for non-additive genotype-by-genotype (G × G) interactions [8] that can reduce the additive genetic variance in the fitness of both parties.

Host-parasite interactions are an excellent model in which to test for G × G effects, because the fitness of each partner depends directly on the genotype of the other: hosts are harmed by their parasites, while parasites must infect a host to survive and reproduce [9]. This mutual dependence makes each party part of the other’s selective environment, so that an adaptation in one selects for a counter-adaptation in the other [10]. The resulting antagonistic coevolution has been implicated in the diversification of immune systems [11], the maintenance of sexual over asexual reproduction [12] and the evolution of sexual selection [13], and it can drive ongoing and exceptionally rapid evolutionary change in both host and parasite.

This rapid evolution is fuelled by high levels of genetic variation in host susceptibility to infection, which have been observed across organisms from bacteria to humans [14–20]. Similarly, parasites exhibit genetic variation in infectivity [5,21]. Although the exact mechanisms maintaining this variation remain the matter of debate [9], in both hosts and parasites coevolution is thought to play a key role [22,23]. Alleles conferring resistance may be costly because they have harmful pleiotropic effects on other traits, or because alleles that confer resistance to one pathogen genotype confer susceptibility to other genotypes. When resistance alleles spread through a population, the prevalence of pathogen genotypes affected by those alleles can decline to a point where these costs outweigh the benefits of resistance [8]. The equivalent process can occur in the parasite population, resulting in negative frequency-dependent selection (NFDS). Importantly, this form of selection can maintain genetic variation in both parties [8].

The rate of evolutionary change in hosts and parasites will depend on the extent to which this genetic variation in host susceptibility and pathogen infectivity results in additive genetic variance in, and is therefore predictive of, transmission and fitness [5]. It is common for infectious disease to be a major determinant of host survival and reproduction, and the ability of pathogens to infect and transmit is strongly affected by host genotype [21,24–26]. However, given nonadditive interactions between host and parasite genotypes (G × G), this may not translate into effects on (average) allelic fitness. For instance, a host allele that has high fitness when exposed to one pathogen genotype may not be beneficial overall if it has low fitness when exposed to a different genotype or when uninfected. Similarly, genetic lineages of pathogens may pass through different host genotypes where they have high and low fitness. The premise that genotype-by-genotype interactions on fitness can enable NFDS and so maintain global genetic variance is recognised by game theoretic and quantitative genetic treatments of coevolutionary dynamics across many different types of biological interaction [27,28]. NFDS can lead to stable equilibrium allele frequencies—at which point all host and pathogen alleles have the same expected fitness [29]. This means there is no additive genetic variation for fitness among hosts or pathogens at equilibrium. Nevertheless, the outcome of each infection still depends on non-additive genetic factors–specifically the interaction of specific host and pathogen genotypes. However, time lags in evolutionary responses mean that coevolutionary models of host-parasite systems often do not predict stable equilibria, but instead generate temporal fluctuations in the direction of selection and therefore allele frequencies [8]. By preventing the population from reaching a stable equilibrium, these temporal cycles in selection can potentially move genetic variance back into main effects, restoring genetic variance in fitness.

The sigma virus (DMelSV) is a negative-sense RNA virus (family *Rhabdoviridae*) and a natural pathogen of *Drosophila melanogaster* that infects 0%–20% of wild flies [30–32]. DMelSV is transmitted vertically through eggs and at a lower rate through sperm [30,33–37], and laboratory and field studies have estimated that infection results in a ~20% reduction in fitness [30,38,39] (Fig 1A). Incomplete vertical transmission, combined with the fitness costs of infection, prevents the virus from reaching fixation and instead maintains it at low prevalence. Although transmission through sperm occurs less efficiently than transmission through eggs, it is required to maintain the virus in the population [33]. In *D. melanogaster* populations, there is considerable genetic variation in viral titres and transmission [30]. Resistant alleles of three genes—*p62* (*ref(2)P*) [14,40], *CHKov1* [41], *Ge-1* [42]—reduce both viral replication and transmission. The effects of these alleles on transmission differ between males and females [14,43]. A number of other major-effect genes and a substantial polygenic component also influence viral load [23]. There is also genetic variation in the viral population, with some viral genotypes escaping the effects of the *p62* resistance allele [44–46]. The spread of the *p62* resistance allele has driven the evolution of DMelSV viral types capable of infecting resistant flies [32,45]. This viral type appears to be recent [46]; European DMelSV populations containing both types shared a common ancestor only ~200 years ago [31]. Its rapid spread was observed in France and Germany during the 1980s [47], where it largely replaced the viral type permissive/sensitive to *p62* over about a decade.

**Fig 1:**
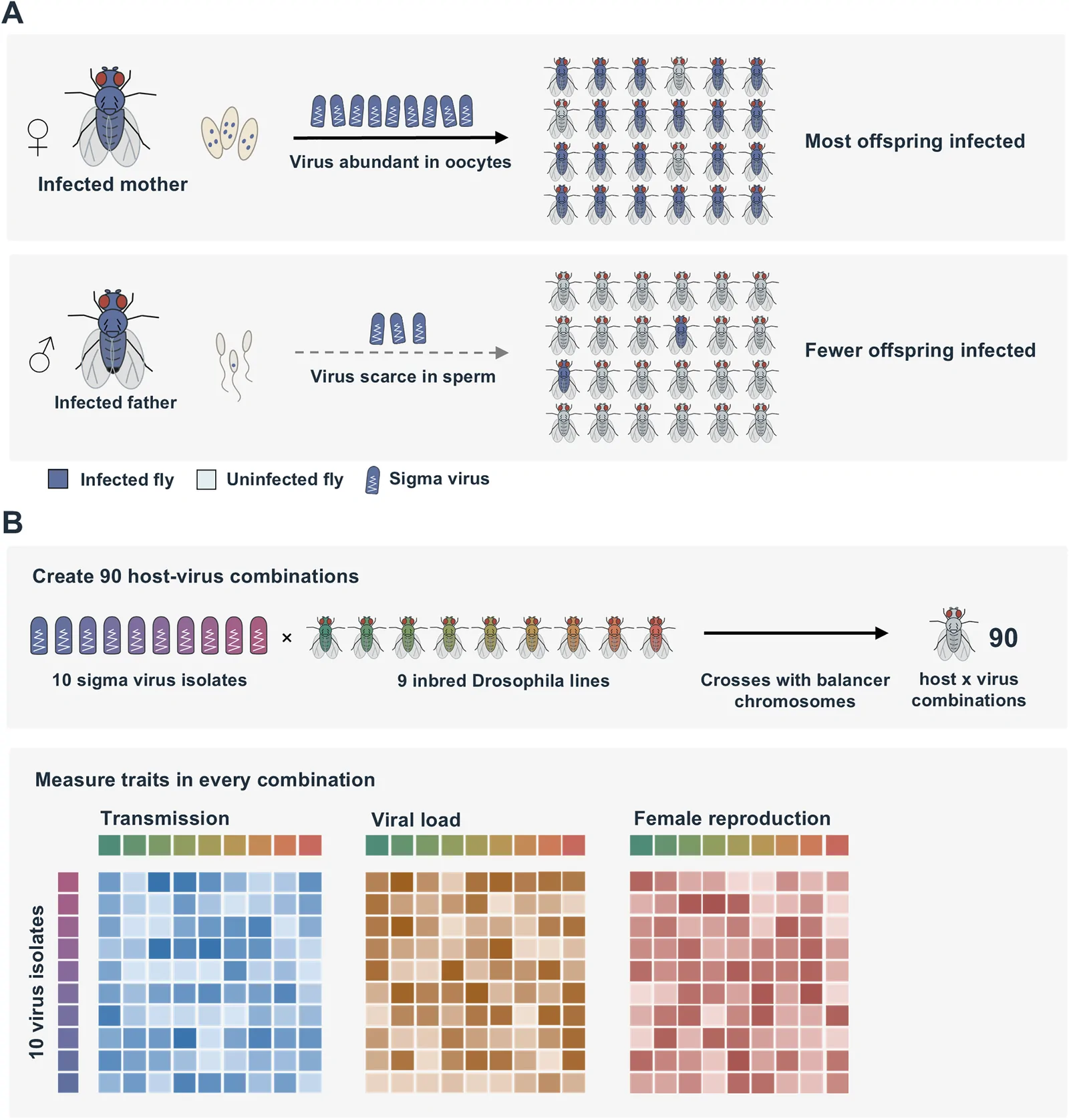
Sigma virus transmission and experimental design. **(A)** DMelSV is vertically transmitted through eggs and sperm, with maternal transmission generally more efficient. **(B)** 10 virus isolates were introduced into nine inbred *Drosophila melanogaster* genotypes using crosses with balancer chromosomes, generating 90 host-virus combinations. Transmission, viral load, fecundity, and egg hatch rate were measured in each combination. This figure is schematic and has no underlying numerical data.

The observation that DMelSV carries a substantial cost to the host is at first sight surprising as vertically transmitted parasites rely on host survival and reproduction for their own transmission. A classical explanation is that there is a trade-off between transmission rate (proportion offspring infected) and virulence (number of offspring), mediated by viral load [48–56]. Therefore, increasing viral replication may enhance transmission fidelity while simultaneously reducing host fitness. Consequently, viral replication provides a mechanism by which the evolutionary interests of host and parasite become decoupled, generating an intrinsic conflict that can promote reciprocal coevolutionary dynamics [57–63].

To investigate the role of host-genotype-by-virus-genotype interactions, we combined modelling with empirical experiments of *D. melanogaster* and DMelSV. By infecting a panel of host genotypes with a panel of viral isolates (Fig 1B), we find that host and viral genotypes strongly influence transmission, viral load, and virulence. However, consistent with coevolutionary predictions [64], much of this variance reflects pronounced genotype-by-genotype interactions. Thus, despite extensive genetic effects on infection outcomes, coevolution has reduced heritable variation in fitness for both host and pathogen, potentially limiting the response to directional selection.

## Results

### Coevolution creates genetic interactions that reduce fitness variance

To examine coevolutionary dynamics in a vertically transmitted virus, we simulated a host population segregating a resistance allele and a viral population segregating for an escape allele that is insensitive to/can overcome host resistance, as observed for DMelSV in nature. In line with classical gene-for-gene models (S1 Fig), both host resistance and viral escape incurred costs [65]. We used parameter values within the empirical ranges estimated for natural *Drosophila*-sigma virus populations [30,35–39], with resistance cost (for which no field estimate is available) varied across a broad plausible range. The system most commonly produced oscillatory coevolutionary dynamics, of two types: damped oscillations converging on a stable polymorphism in host and virus (Figs 2A–2C and S2A–S2C and S3A–S3C), or persistent cycles in allele frequencies in both populations (Figs 2D–2F, S2D–S2F and S3D–S3F). With no cost of resistance, there was a partial selective sweep of host resistance that stalled when the viral escape genotype became fixed (S4A–S4C Figs).

**Fig 2:**
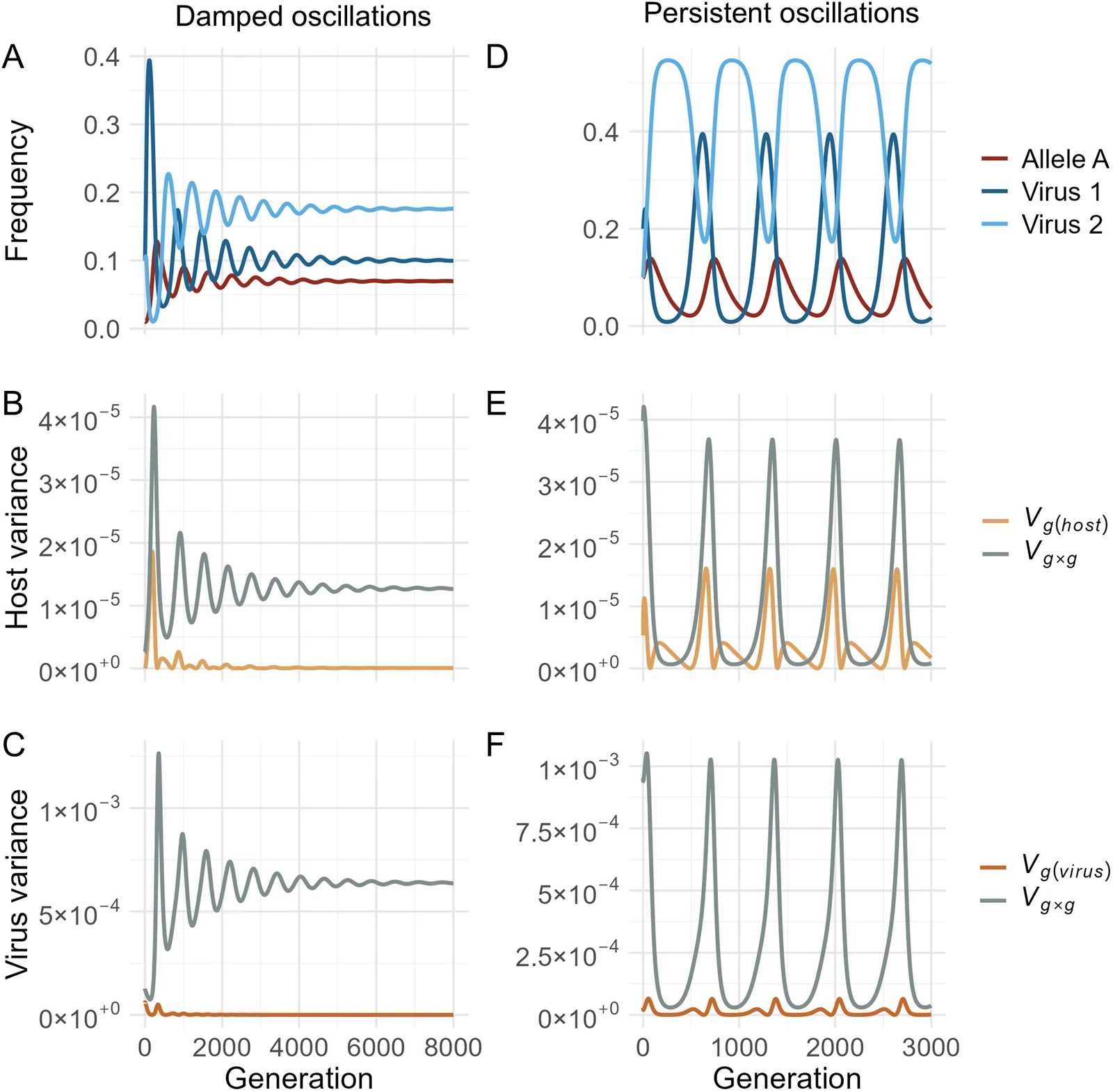
Time series of coevolutionary dynamics between a host and vertically transmitted pathogen under a gene-for-gene model. The host carries one biallelic resistance locus (*A/a*), where allele *A* is dominant and partially resistant to the virus. The virus has two genotypes, where Virus 1 is susceptible to host allele *A* and Virus 2 is an escape variant that is not affected. The left column **(A–C)** illustrates damped oscillations converging on a stable polymorphism (*t*_*1*_ = 0.68, *t*_*2*_ = 0.66, *c* = 0.23, *r* = 0.27, *d* = 0.006; started from infected frequencies of 0.1 for Virus 1 and 0.1 for Virus 2 and a resistance-allele *A* frequency of 0.01, run for 8,000 generations). The right column **(D–F)** shows persistent allele-frequency cycles (*t*_*1*_ = 0.72, *t*_*2*_ = 0.70, *c* = 0.255, *r* = 0.27, *d* = 0.007; started from infected frequencies of 0.2 for Virus 1 and 0.1 for Virus 2 and a resistance-allele *A* frequency of 0.1, run for 3,000 generations). Parameters are *t*_*1*_ and *t*_*2*_, the base vertical-transmission rates of Virus 1 and Virus 2; *c*, the viability cost of infection on offspring; *r*, the proportional reduction in virus transmission in hosts carrying a matched resistance allele; *d*, the per-locus fertility cost of carrying a dominant resistance allele. (A, D) Frequencies of the two viral genotypes and of resistance allele *A* across generations. (B, E) Host-fitness genetic variance partitioned into the additive host component *V*_*G(host)*_ (orange) and the host × virus interaction *V*_*G×G*_ (grey). (C, F) Viral-fitness variance partitioned analogously into *V*_*G(virus)*_ (red) and *V*_*G×G*_ (grey). The per-generation values underlying all panels can be found in S2 Data.

To link these dynamics to fitness, we decomposed the genetic variance in fitness into additive components for host and virus, *V*_*G(host)*_ and *V*_*G(virus)*_, and a genotype-by-genotype term, *V*_*G×G*_, capturing genetic effects on infection outcomes that do not translate into heritable fitness (for the host, this term also includes differences in the fitness of infected and uninfected individuals). With damped oscillations (Fig 2A), additive genetic variances *V*_*G(host)*_ and *V*_*G(virus)*_ declined towards zero as equilibrium was approached, while *V*_*G×G*_ remained high (Fig 2B and 2C). This is an expected signature of NFDS eroding any net advantage of individual genotypes. In contrast, persistent cycles (Fig 2D) maintained additive genetic variance in fitness—*V*_*G(host)*_ and *V*_*G(virus)*_—through time (Fig 2E and 2F). However, *V*_*G×G*_ typically dominated for both host and viral fitness, although this oscillated through time along with the genotype frequencies. These qualitative patterns were robust across different genetic interactions, such as matching-allele and multi-locus gene-for-gene models (S2 and S3 Figs; middle and bottom). When there are selective sweeps of viral escape alleles and partial sweeps of host resistance, *V*_*G×G*_ becomes important for the host once viral escape becomes common (S4B Fig).

To investigate these dynamics more systematically, we explored a range of parameter values (Fig 3; full parameter sweeps in S6 and S7 Figs). The coefficient of variation (CV) of viral-genotype frequencies (Fig 3A) tracks the dynamical regime, with lower values corresponding to damped oscillations approaching a stable equilibrium. In these regions of parameter space, the fitness variance was concentrated in the interaction term, with the proportion of additive genetic variance approaching zero for both host and virus (*V*_*G*_ / [*V*_*G*_ + *V*_*G×G*_]; Fig 3B and 3C). A higher CV marks parameters where the polymorphism persists through large, ongoing allele-frequency cycles (Fig 3A). These cycles repeatedly shift variance onto the host and virus main effects, so the additive share of fitness variance is higher when genotype frequencies fluctuate more (Fig 3B and 3C). The extent to which genotype frequencies fluctuate depends on the costs of infection and resistance (Fig 3A), and this relationship in turn changes depending on other model parameters (S6 and S7 Figs). Even so, when averaged over time the interaction term was larger than the corresponding additive component in most, but not all, parameter combinations that maintained polymorphism (Figs 3B and 3C; S6 and S7). Additive components could also briefly exceed the interaction component within individual generations. Thus, genotype-by-genotype interactions arising during coevolution are expected, in most conditions, to substantially depress additive genetic variance in host and virus fitness.

**Fig 3:**
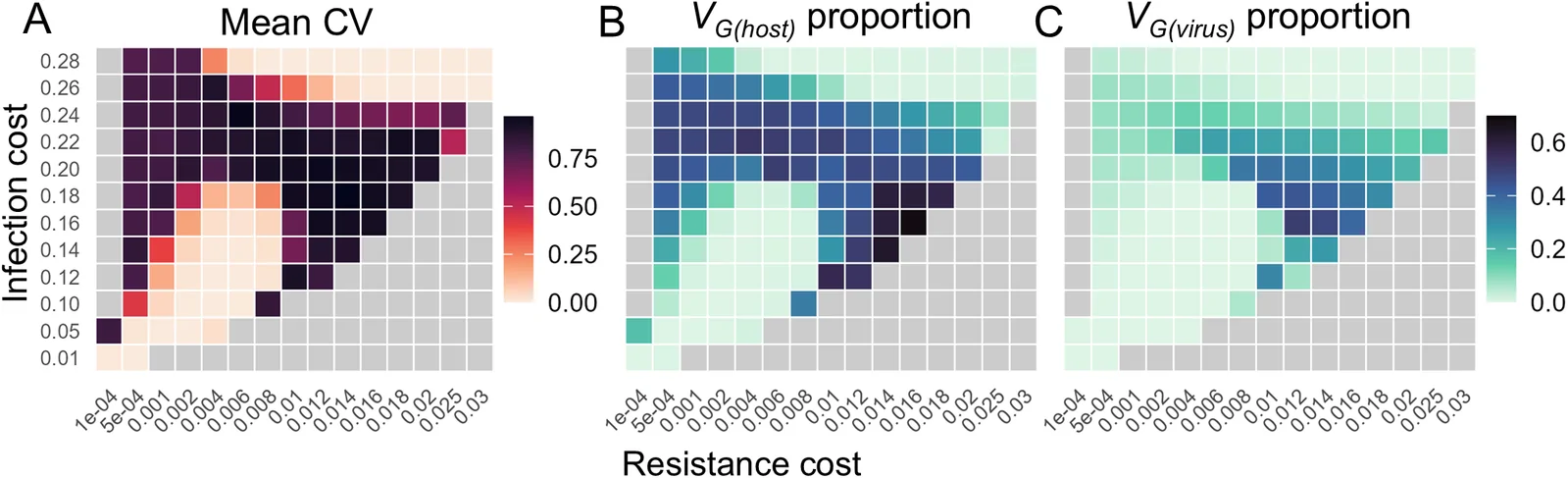
Coevolutionary dynamics and fitness variance across parameter space. Coevolution was simulated as in Fig 2, assuming a gene-for-gene model, where one viral genotype escapes the effects of a host resistance allele. Plots summarise a range of fertility costs of resistance *d* and viability cost of infection *c*. Transmission rates in susceptible hosts were fixed at *t*_*1*_ = 0.73, *t*_*2*_ = 0.70 (within the empirically observed sigma virus range), and the resistance effect at *r* = 0.27. Each run started from initial frequencies of 0.2 (Virus 1), 0.1 (Virus 2) and 0.1 (resistance allele *A*). Each cell is a 40,000-generation run with statistics computed over the final 20,000 generations. **(A)** The mean coefficient of variation (CV) of viral-genotype frequencies, averaged across the two viral strains. **(B)** The proportion of additive genetic variance in host fitness, *V*_G(host)_ / [*V*_G(host)_ + *V*_G×G_]; and **(C)** the corresponding proportion for viral fitness. Variance components in (B) and (C) were restricted to generations with viral prevalence > 0.01%. Parameter combinations leading to the loss of either both viruses or just allele *A* are shown as grey cells. More extensive exploration of parameter space is given in S6 and S7 Figs. The data supporting these figures can be found in S2 Data.

Across most of the parameter space, polymorphisms were not maintained so coevolution ceased (loss of one or both virus genotypes or loss of host resistance). Across the two resistance effects, 80% of the parameter combinations producing oscillations had base transmission rates of *t*_1_ = 67%–83% and *t*_2_ = 66%–80%, and infection costs were about 1% to 30%. These ranges overlap empirical estimates of transmission and virulence in wild *Drosophila*-sigma virus populations (S6 and S7 Figs; detailed parameter ranges, outcome classifications, and a broader exploration of parameter space are provided in S1 Text) [30,39,66].

In our experiments, vertical-transmission rates are measured between adult generations, so they do not reveal whether virus loss occurs during transmission to gametes or during offspring development. To check our assumptions are not affecting our conclusions, we explored the effects of viral loss at different stages in the life cycle. While these changes did alter the boundaries where the dynamics shifted between regimes, oscillatory dynamics were consistently present and our qualitative conclusions remained (S8 Fig; S1 Text).

### Experimental data confirm genetic variation in sigma virus fitness is strongly reduced by G_H_ × G_P_ interactions

The fitness of a vertically transmitted pathogen is determined by the number of offspring produced by infected hosts and the rate at which the virus is transmitted to those offspring. We quantified how host and viral genotypes influence sigma virus transmission rates (Fig 4A and 4B) by infecting nine *D. melanogaster* lines from the *Drosophila* Genetic Reference Panel (DGRP) [67,68] with 10 strains of sigma virus. As the virus is vertically transmitted, we crossed viruses into the fly lines (via backcrossing with balancers), ensuring natural transmission dynamics [37]. This crossing design generated all 90 combinations independently, decoupling viral isolate from host genetic background. As the virus is haploid and nonrecombining, its genome is transmitted clonally. There is no dominance, and any epistasis among viral loci is inherited intact and so contributes solely to the additive component of genetic variance. The virus main-effect variance is therefore an estimate of the additive genetic variance. The host lines are inbred, so the main-effect variance will capture nonadditive (dominance and epistatic) variance. Nevertheless, most genetic variance is theoretically expected to be additive [69], so this term should predominantly reflect additive genetic variance.

**Fig 4:**
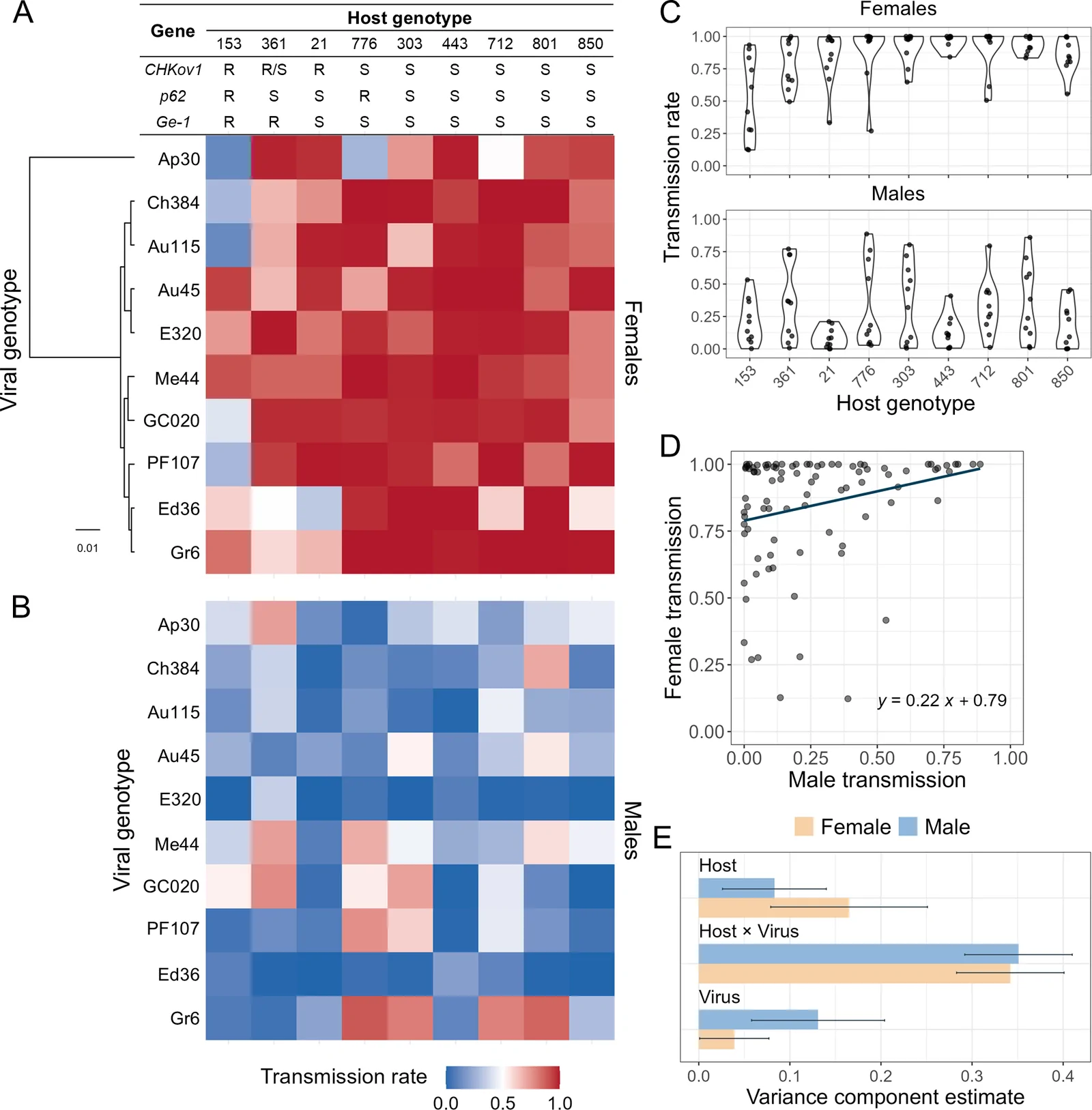
The effect of host and viral genotype on vertical transmission rate. **(A)** The proportion of progeny that were infected from an infected mother crossed to an uninfected father. Infection was detected using the CO_2_ assay. Host genotypes are annotated for three loci with known resistance (R) or susceptibility (S) alleles; heterozygotes are denoted R/S. The phylogeny of the virus strains is shown to the left; all nodes are supported with posterior probability ≥99.9%. **(B)** The proportion of progeny that were infected from an infected father crossed to an uninfected mother. **(C)** The distribution of transmission rates across host genotypes. **(D)** Phenotypic correlation between maternal and paternal transmission. Each point is a unique combination of host and viral genotypes. **(E)** Variance components estimated from an ASReml model with standard errors. The data supporting panels A–D can be found in S1 Data, and the values for panel E are provided in Table B in S1 Text. The sequence alignment and tree underlying the phylogeny are provided in S3 Data.

In line with known transmission patterns, we observed strong sex differences: on average infected females transmitted the virus to 85% of their offspring, compared to just 26% for infected males (Fig 4C and 4D). Across all host-virus combinations, transmission rates were correlated between sexes (phenotypic correlation: *r*_*p*_ = 0.25, *p* = 0.016; Fig 4D; all phenotypic correlations are given in Table G in S1 Text). However, while low-transmitting female genotypes never exhibited high male transmission, the reverse is not true (Fig 4D). The existence of lines where transmission by females is near-perfect but transmission through males is strongly reduced suggests specific barriers that may affect entry into the male germline. Despite this disparity in mean transmission, variability was similar between sexes (female standard deviation = 0.22; male standard deviation = 0.25).

In females, the combined genetic effects of the host or virus accounted for 55% of total transmission variance. However, this does not correspond to high genetic variation in viral fitness (as averaged across hosts), because the majority of this genetic variance in transmission was attributed to host-virus genotype-by-genotype (G_H_ × G_P_) interactions (63%; Intraclass Correlation Coefficient: ICC = 0.34 ± 0.06 SE, *p* < 0.001; ICC represents proportion of total phenotypic variance; Fig 4E; all genetic variance components are shown in Table B in S1 Text), while only 7% was explained by genetics of the virus alone (ICC = 0.04 ± 0.04 SE, *p* = 0.076; Fig 4E). Therefore, a virus that has high transmission in one host genotype will frequently have low transmission in future generations when it finds itself in a different genotype. For instance, despite the virus strain Ap30 having high transmission in most host genotypes, transmission was strongly reduced in host lines 153 and 776. In this case this likely reflects sensitivity to the resistant allele of *p62* (Fig 4A).

The pattern was similar in males (Fig 4E). Genetic influences of the host or virus again explained the majority of transmission variance (56%). However, G_H_ × G_P_ interactions contributed the largest proportion (62% of genetic variance; ICC = 0.35 ± 0.06 SE, *p* < 0.001), exceeding the effect of viral genotype alone (main effect virus: 23% genetic variance; ICC = 0.13 ± 0.07 SE, *p* < 0.001). For example, virus strain Gr6 had the highest mean transmission in males (0.45), more than 7-fold higher than the lowest-transmitting strain (Fig 4B and 4C). However, this was driven by high rates in four host genotypes, with strongly reduced transmission in other lines.

For a vertically transmitted pathogen, transmission rate also affects host fitness. High transmission rates mean more of an individual’s offspring suffer the costs of infection. Mirroring the effects in viruses, G_H_ × G_P_ interactions strongly reduced the influence of this factor on host fitness. In females, host genotype explained 30% of the transmission genetic variance (ICC = 0.17 ± 0.09 SE, *p* < 0.001), compared to 63% explained by G_H_ × G_P_ interactions (Fig 4E). In males, host genotype explained 15% of the genetic variance (ICC = 0.08 ± 0.06 SE, *p* = 0.006) compared with 62% explained by G_H_ × G_P_ term (Fig 4E). Therefore, as was the case in the virus, despite high levels of host genetic variation, the host fitness variation is greatly reduced by G_H_ × G_P_ interactions. These interactions persisted when the analysis was restricted to the five susceptible lines, although both the interaction and host main-effect estimates were lower and less precise with fewer lines (Table F in S1 Text). Restricting the analysis to the seven UK viral isolates likewise left G_H_ × G_P_ as the largest genetic component of transmission in both females (65%; ICC = 0.32 ± 0.07 SE, *p* < 0.001) and males (53%; ICC = 0.29 ± 0.07 SE, *p* < 0.001; Table I in S1 Text).

Despite the phenotypic correlations between sexes (Fig 4D), the underlying genetic architecture of transmission was markedly sex specific. The host-virus interaction effects, which dominated the genetic variance in both sexes, were only partially correlated across sexes (*r*_*g*_ = 0.34 ± 0.12 SE; all cross-sex genetic correlations are reported in Table D in S1 Text), indicating substantial genotype-by-sex interactions. The cross-sex correlations of the viral and host main effects were poorly estimated (virus: *r*_*g*_ = 0.54 ± 0.44 SE; host: *r*_*g*_ = 0.17 ± 0.46 SE).

### Sex-specific interactions between host and virus genotypes determine viral load

Viral load is frequently a key determinant of host and viral fitness, mediating both transmission rates and virulence. We quantified viral load in 347 pooled RNA samples, comprising 172 female and 175 male pools and representing 806 female and 774 male flies across all 90 host-virus genotype combinations (Fig 5A and 5B), observing a significant phenotypic correlation between sexes (*r*_*p*_ = 0.50, *p* < 0.001; Fig 5D), showing that host-virus combinations with higher viral load in females also tended to have higher viral load in males.

**Fig 5:**
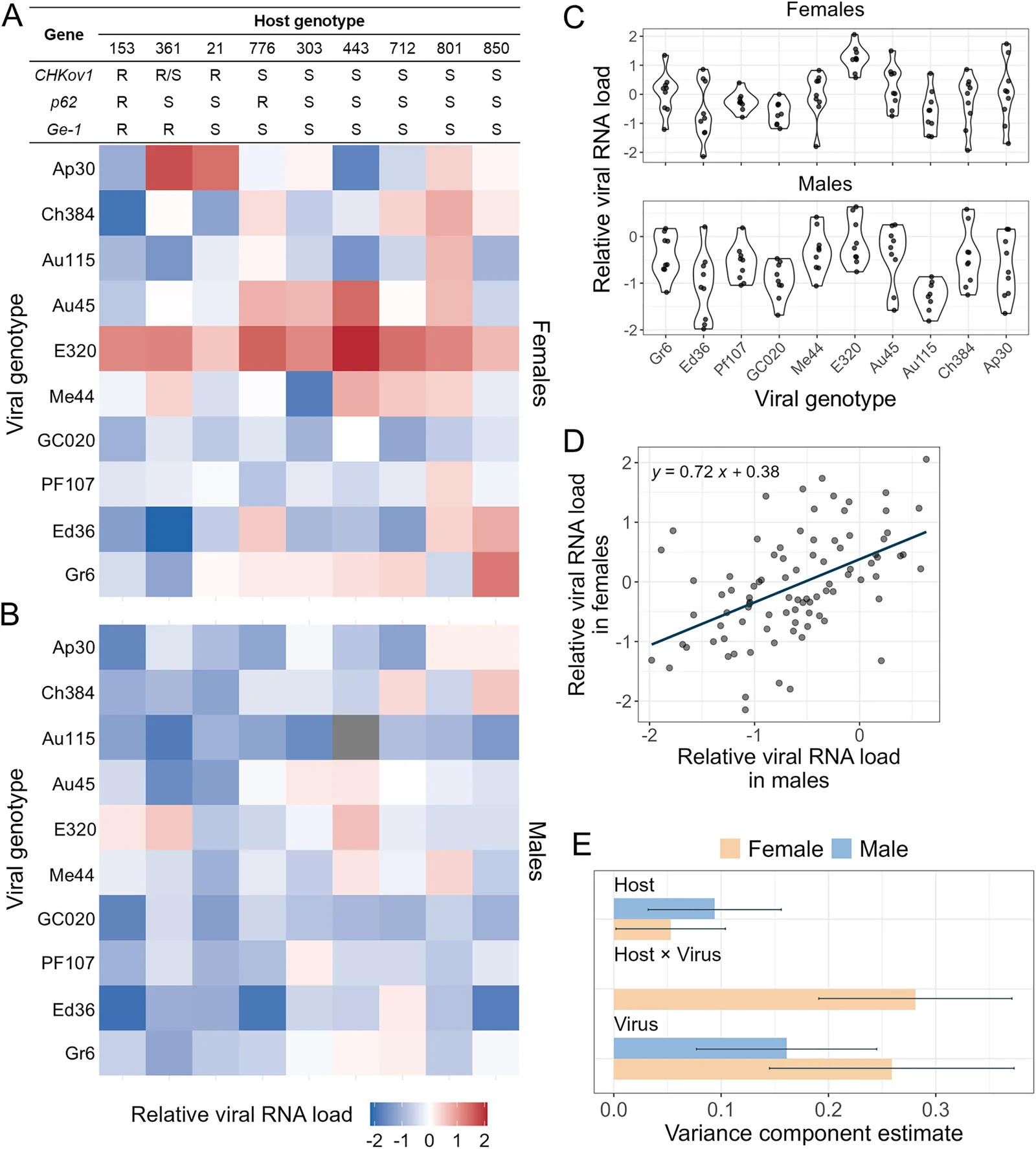
The effect of host and viral genotype on viral load. **(A)** Viral load in females across 90 combinations of *D. melanogaster* lines and sigma virus strains. **(B)** Viral load in males across the same host-virus combinations. Viral RNA was quantified by qPCR relative to the mean-centred expression of the host gene *RpL32*. Viral load estimates are based on 347 RNA samples from ~1,580 flies. The Au115–DGRP443 combination lacked a male viral-load estimate. **(C)** Distribution of viral load by virus genotype. **(D)** Correlation of viral load between sexes across all unique host-virus combinations. Line indicates linear regression. **(E)** Variance component estimates from ASReml mixed models with standard errors. The absence of a male interaction term reflects a variance component not detected in the model. The data supporting panels A–D can be found in S1 Data, and the values for panel E are provided in Table B in S1 Text.

In females, viral load varied by 18-fold, with 59% of phenotypic variance attributable to genetic effects. Notably, G_H_ × G_P_ interactions accounted for the largest share of the total genetic variance (47%; ICC = 0.28 ± 0.09 SE, *p* < 0.001; Fig 5E). For instance, virus strain Me44 typically exhibited high viral titres across hosts but showed markedly reduced load in line 303 (Fig 5A). Viral genotype alone explained 44% of the total genetic variance in females (ICC = 0.26 ± 0.11 SE, *p* < 0.001), with the highest-titre strain (E320) reaching approximately four times the mean load of the lowest (GC020; Fig 5A and 5C). Although all three known host genetic polymorphisms that restrict sigma virus replication were present among the DGRP lines, their effects were inconsistent across viral genotypes (Fig 5A and 5C). Consequently, host genotype alone explained only 9% of the total genetic variance in females (ICC = 0.05 ± 0.05 SE, *p* = 0.067). Nonetheless, the line with the lowest overall viral load (line 153) was the only genotype carrying the resistant allele at all three loci (Fig 5A).

In males, genetic contributions to viral load were substantially reduced, with only 25% of phenotypic variance explained by host and viral genotypes. Unlike in females, G_H_ × G_P_ interactions were not detectable (Fig 5E). Viral genotype accounted for the majority of total genetic variance in males (63%; ICC = 0.16 ± 0.08 SE, *p* < 0.001; Fig 5E), with strain E320 again exhibiting the highest viral load (Fig 5B and 5C). Host genotype explained the remaining 37% of total genetic variance (ICC = 0.09 ± 0.06 SE, *p* = 0.003; Fig 5E).

Together, these findings point to sex differences in the genetic architecture of viral load, with G_H_ × G_P_ interactions a detectable contributor in females but not in males. Restricting the analysis to the five susceptible lines gave the same pattern (Table F in S1 Text). With the seven UK isolates, G_H_ × G_P_ remained undetectable in males and significant in females, but in females it fell to 35% of the genetic variance (ICC = 0.22 ± 0.10 SE, *p* = 0.002), below the host main effect (ICC = 0.31 ± 0.15 SE, *p* = 0.018; Table I in S1 Text). Therefore, the interaction variance in female viral load depends on the viral genotypes assayed, although estimates from only seven isolates are less precise. The cross-sex correlations of the host and viral main effects were positive but imprecisely estimated (main effect host genotype: *r*_*g*_ = 0.76 ± 0.43 SE; the viral estimate was bound near one, with the among-virus variance close to zero, and is not interpretable; Table D in S1 Text). These estimates are consistent with some shared genetic influences on viral load across sexes, but do not establish their strength.

### Transmission is positively genetically correlated with viral load

The relationship between pathogen load and transmission potential is a common assumption in disease ecology [56]. While high pathogen loads are frequently associated with increased transmission rates in horizontally transmitted systems [50], in vertically transmitted parasites the relationship is more variable than this generalisation suggests, with both the strength and direction differing across host-parasite systems [70]. Although it has received theoretical attention [71], to our knowledge no study has separated the host and viral genetic contributions to this relationship empirically. We found that transmission is positively correlated with viral load in both females (*r*_*p*_ = 0.35, *p* < 0.001; Fig 6A) and males (*r*_*p*_ = 0.21, *p* = 0.044; Fig 6B), suggesting that physiological mechanisms linking replication to transmission, such as viral access to gametes, operate in both sexes.

**Fig 6:**
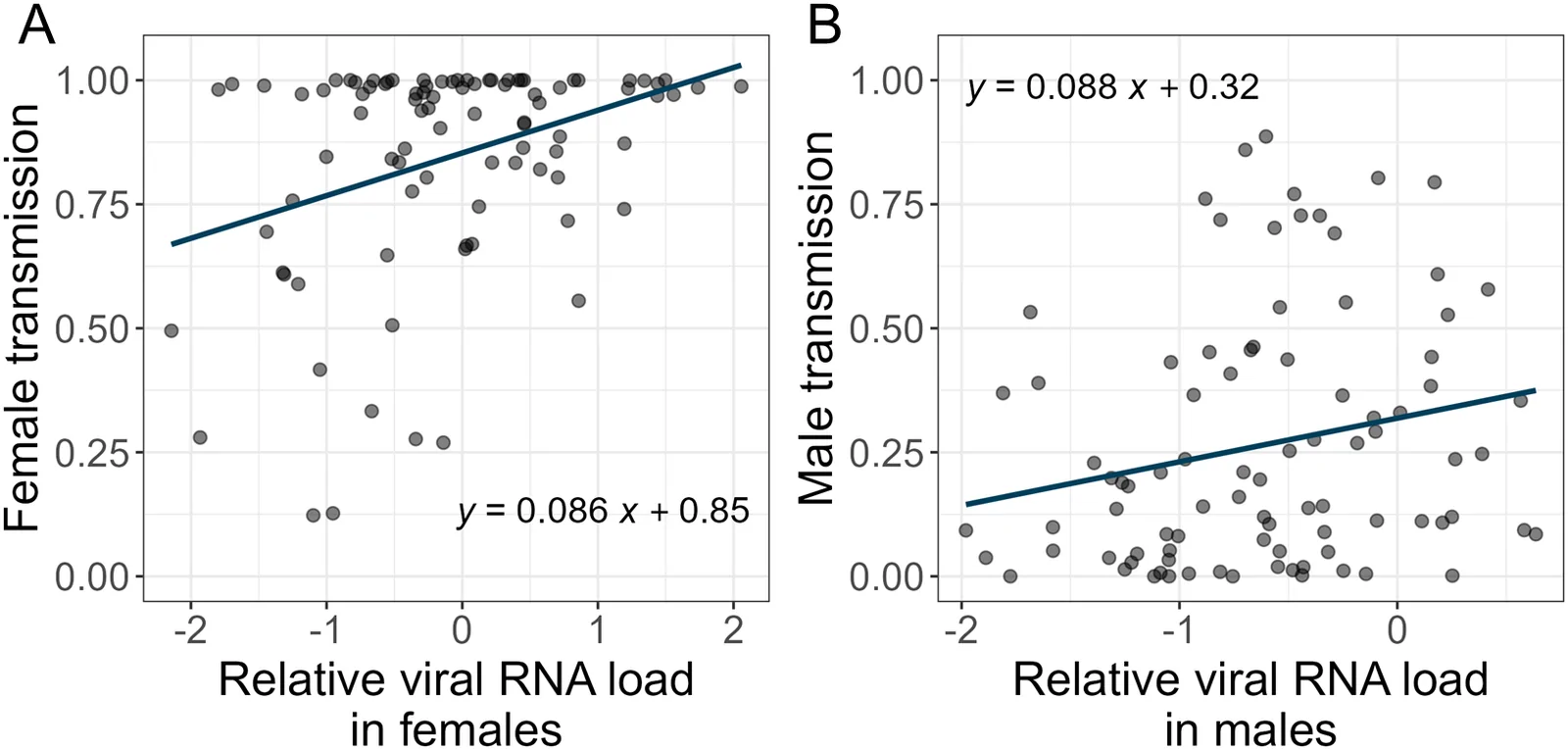
The impact of viral load on the transmission rate. The relationship between viral load and the proportion of progeny that were infected **(A)** maternally and **(B)** paternally. Viral load is relative to *RpL32* expression and is shown for females in (A) and males in (B). Each point is a unique combination of host and viral genotypes. Linear regression lines are shown. The data supporting these figures can be found in S1 Data.

To disentangle the genetic contributions to this correlation, we partitioned cross-trait genetic correlations (*r*_*g*_) by host genotype, viral genotype, and host-virus genotype interactions. Although most *r*_*g*_ estimates exhibited substantial standard errors, consistent trends emerged. In females, the interaction term showed a positive genetic correlation (host-virus interaction *r*_*g*_ = 0.42 ± 0.15 SE; all between-trait genetic correlations are reported in Table C in S1 Text). The main effects in females and all effects in males were poorly estimated (Table C in S1 Text). Therefore, despite a lack of statistical power for most correlations, the female interaction estimate suggests partly shared genetic determination of viral load and transmission.

### Genotype specificity is partly structured by viral phylogeny

To examine whether the G_H_ × G_P_ interaction reflected viral evolutionary history, we tested whether related isolates shared similar host-specific effects. For each trait, we removed each isolate’s mean and compared Euclidean distances between the resulting G_H_ × G_P_ profiles with patristic genetic distance on the viral phylogeny (Fig 4A) across the 45 pairs of isolates. Closely related isolates had more similar G_H_ × G_P_ profiles for female viral load (Mantel *r* = 0.71, Holm-adjusted *p* = 0.041). Female transmission showed a positive but nonsignificant relationship (*r* = 0.63, adjusted *p* = 0.223), while neither male viral load (*r* = 0.40, adjusted *p* = 0.347) nor male transmission (*r* = 0.06, adjusted *p* = 0.347) was significantly associated with phylogenetic relatedness (Table H in S1 Text).

### Interactions between host and virus genotype reduce the additive genetic variance in host reproduction

The fitness consequences of infection for the host are determined by viral virulence—the degree of harm inflicted on the host. Because vertically transmitted viruses depend on host survival and reproduction for their own propagation, virulence also directly constrains viral fitness in this system. We quantified virulence across 90 host-virus genotype combinations by measuring fecundity (eggs laid) and hatch rate (proportion of eggs hatched; Fig 7A and 7B). As the virus is passed on through the eggs, these traits affect the fitness of both the host and the virus. After normalising for baseline differences among host genotypes, hatch rate and fecundity were weakly and not significantly correlated (*r*_*p*_ = 0.17, *p* = 0.12; Fig 7C). For some host-virus combinations, mean fecundity or hatch rate exceeded the corresponding uninfected control (Fig 7A and 7B).

**Fig 7:**
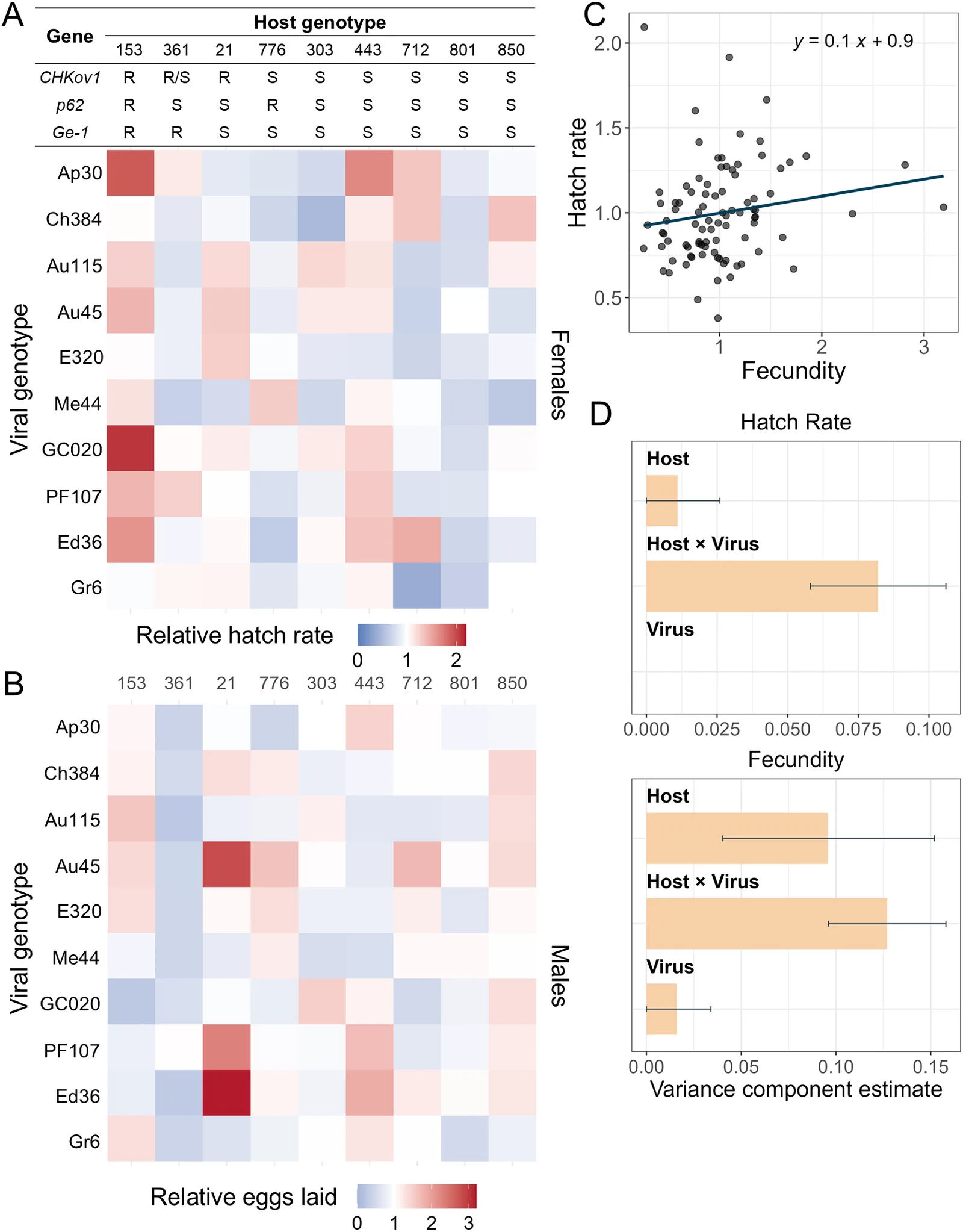
The effects of infection on female reproductive traits. The effect of viral infection on **(A)** The hatch rate and **(B)** relative number of eggs laid (fecundity). Both statistics are relative to the estimate from uninfected flies. **(C)** The correlation between the number of eggs laid and the proportion of eggs that hatch. Each point is a unique combination of host and viral genotypes. Eggs were laid by individual 6–7-day-old females over a 24-hour period, and the proportion that hatched was counted 48 hours later. **(D)** Variance-component estimates for hatch rate and fecundity from an ASReml model. Error bars indicate standard errors. The absent virus component for hatch rate reflects a variance component not detected in the model. The data supporting panels A–C can be found in S1 Data, and the values for panel D are provided in Table B in S1 Text.

To partition genetic effects, we included the mean trait value of each uninfected line as a covariate in the corresponding fecundity and hatch-rate model. Hatch rate and fecundity were positively correlated at the level of host-virus interactions (*r*_*g*_ = 0.51 ± 0.18 SE). The host-level correlation was too imprecisely estimated to interpret (*r_g_* = −0.74 ± 0.64 SE). However, the combined effects of host and viral genetics accounted for only 9% of the phenotypic variance in hatch rate and 24% in fecundity, substantially less than for viral load or transmission and indicating high residual variability in reproductive traits. Within the genetic component, the large majority of total genetic variance in hatch rate was attributable to G_H_ × G_P_ interactions (88%, ICC = 0.082 ± 0.024 SE, *p* < 0.001; Fig 7D). G_H_ × G_P_ effects also explained 53% of the total genetic variance in fecundity (ICC = 0.127 ± 0.031 SE, *p* < 0.001; Fig 7D). Thus, host-virus genetic interactions are a detectable contributor to variation in the reproductive effects of infection, but they do not account for most of the observed phenotypic variation.

The main effect of viral genotype did not significantly influence hatch rate or fecundity (Fig 7D), indicating no detectable effect of viral genotype on host reproduction at the population level. Host genotype effects were similarly weak for hatch rate (ICC = 0.011 ± 0.015 SE, *p* = 0.155) but accounted for 40% of the total genetic variance in fecundity (ICC = 0.096 ± 0.056 SE, *p* < 0.001, Fig 7D).

We next tested for a trade-off between transmission and virulence, expected if higher replication enhances transmission at the expense of host fitness [50,72]. Phenotypically, correlations of maternal and paternal transmission with fecundity, hatch rate, and relative larval offspring were all negative, consistent with such a trade-off, although only three were significant (Figs 8A and 8B, S5). Genetically, we found no evidence for the trade-off. Correlations at the G_H_ × G_P_ interaction level were close to zero (for example, fecundity and male transmission, *r_g_* = −0.01 ± 0.15 SE), while host-level estimates had standard errors too large to interpret (Tables C and D in S1 Text).

**Fig 8:**
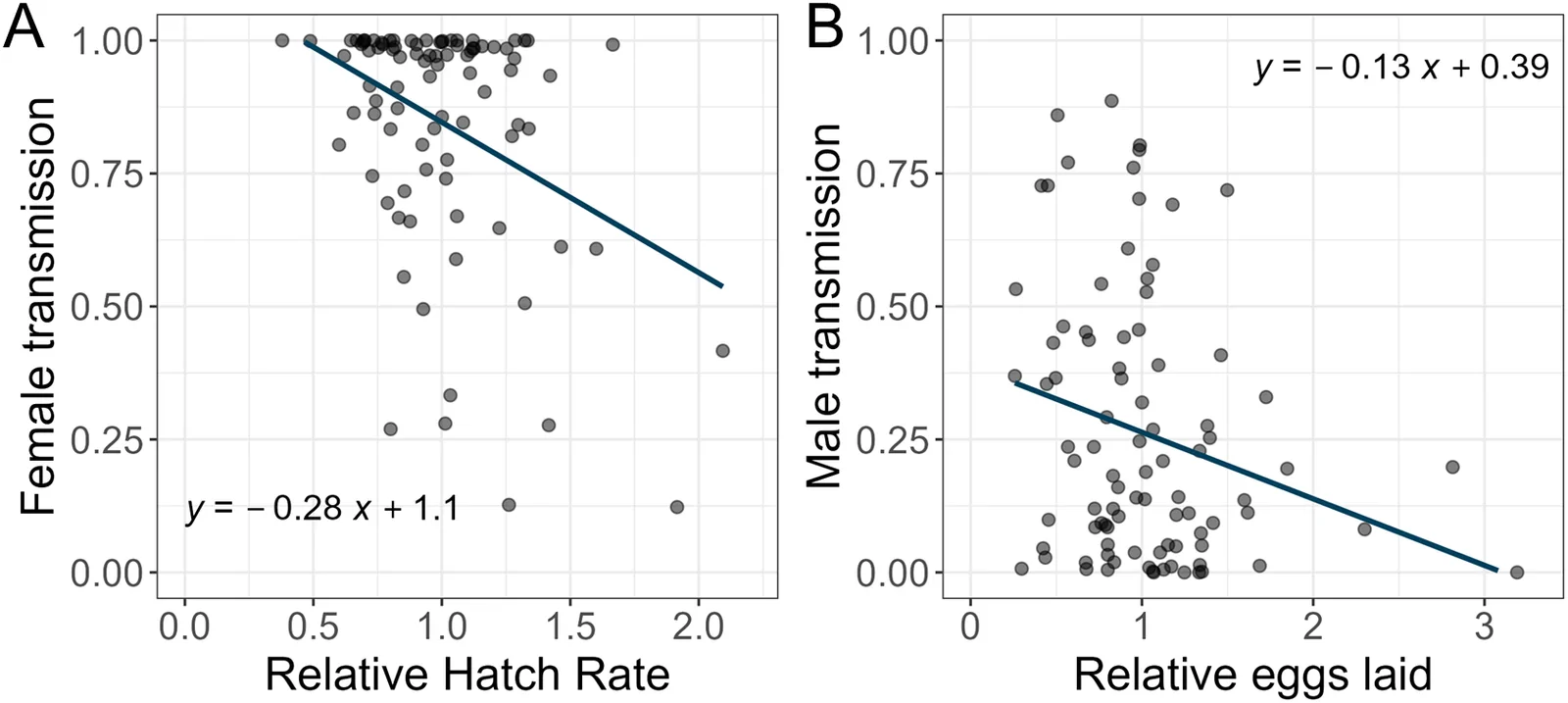
Relationships between transmission and host reproductive traits. **(A)** Correlation between relative hatch rate and female transmission. **(B)** Correlation between relative fecundity and male transmission. Each point is a unique combination of host and viral genotypes. Linear regression lines and equations are shown. The data supporting these figures can be found in S1 Data.

## Discussion

A central question in coevolution is what maintains genetic variation in interacting partners despite ongoing selection. One explanation lies in genotype-by-genotype effects on fitness that can result in NFDS, in which no genotype gains a permanent advantage. This selection shapes the structure of fitness variance: the fitness of any genotype depends on which partner genotypes it encounters, shifting genetic variance from additive main effects into the interaction term. This will reduce the additive genetic variance in fitness and slow rates of adaptation. To investigate how strong this pattern is in a natural system, and what it reveals about coevolutionary dynamics, we investigated the *Drosophila*-sigma virus system, combining theoretical simulations with large-scale experimental tests.

To establish theoretical expectations, we first modelled host-parasite coevolution in a system with vertical transmission. Although the dynamics were sensitive to parameters, ranging from damped to persistent oscillations, fitness variance for both host and virus was consistently dominated by the *V*_*G×G*_ term. This pattern is robust across both gene-for-gene and matching-allele interaction models, indicating it is a general consequence of host-parasite coevolution with vertical transmission. The parameter space producing these dynamics is consistent with infection parameters measured in natural *Drosophila*-sigma virus populations [30,35–39]. Thus, across much of the biologically plausible parameter space, a snapshot of a natural population is expected to show *V*_*G×G*_ dominating and additive genetic variance in fitness eroded.

Our model is deterministic, describing dynamics in the absence of drift. NFDS opposes the loss of rare alleles, but our models predict that in some parameter space alleles become transiently rare. Here, persistence will depend on the effective population size [73,74]. In small or strongly subdivided populations, host and virus alleles may fix, eliminating the variation on which the interaction term depends. If fixation occurs locally, coevolution may generate differences between rather than within populations, in which case whether G_H_ × G_P_ dominates fitness variance is in part a demographic question. However, in our system we know the resistance and viral polymorphisms segregate within wild populations.

No host genotype in our panel was universally resistant, and no viral genotype was universally infective. Previous work in this system showed that a *p62* resistance allele affected maternal transmission of particular viral isolates and had no effects on isolates that had evolved to escape the effects of this allele [43,45,75,76]. On the other hand, Wilfert and Jiggins found that with high genetic variation in paternal transmission across both host and viral genotypes, the host resistance gene remained effective across virus isolates, suggesting predominantly general resistance [46]. Carpenter and colleagues similarly found positive genetic correlations between transmission rates across host genotypes, though the fact that these correlations were below one indicated that there was a significant G_H_ × G_P_ component [76]. We found that G_H_ × G_P_ was the largest genetic component for every trait except male viral load. For host reproductive traits, however, that genetic component was itself small. Fitness in this system is therefore largely a property of specific host-parasite combinations, with little additive genetic variance available for selection on either party alone.

While the relative strength of G_H_ × G_P_ interactions varies across host-parasite systems [6,7,21,25,77–81], our study revealed strong G_H_ × G_P_ interactions governing most sigma virus infection outcomes. Therefore, despite extensive genetic variation in both host and sigma virus, there may be substantially less variation in the mean fitness of either party in the population. In this case, interactions among genotypes maintain coexistence without any genotype becoming universally dominant [82]. Theory predicts that at a stable equilibrium the host and parasite additive genetic variances approach zero. Fitness depends on partner genotype, but averaged across partners no host or virus genotype has a consistent advantage for directional selection to act on. This interaction variance is in effect cryptic at the population level. However, while it is inaccessible to directional selection under the current distribution of partner genotypes it will be exposed to selection when that distribution shifts. In practice natural populations may spend much of their time away from the stable equilibrium, because of mutational input of new resistance or escape mutations, environmental change, and demographic changes. Such perturbations can shift the variance into host or pathogen additive components, releasing previously hidden variance and enabling adaptive evolution. In parameter space where genotypes cycle, shifts in allele-frequency of the opposing party are a form of environmental change, periodically shifting the variance to the main effect, allowing rapid adaptation.

Two features of our experimental design could in principle have influenced how variance was partitioned: the host lines were chosen around three major-effect resistance polymorphisms, and the viral isolates came from geographically separated populations rather than from the source population of the host lines. Neither fully explains the interactions in transmission, the trait on which viral fitness primarily depends. G_H_ × G_P_ remained the largest genetic component of transmission in both sexes when either the host panel was restricted to lines carrying the susceptible allele at all three loci or the viral panel was restricted to isolates from the UK (Tables F and I in S1 Text). We also detected no significant relationship between the G_H_ × G_P_ specificity of transmission and viral phylogeny. Female viral load behaved differently, as restricting the viral panel to the UK reduced its interaction variance below the host main effect (Table I in S1 Text). However, the G_H_ × G_P_ interaction variance remained substantial.

Geographical structure will affect our estimates. The best-characterised source of genotype specificity in this system is the sensitivity of viral genotypes to the *p62* polymorphism. This may in part explain why G_H_ × G_P_ effects were clustered on the viral phylogeny, as the escape type arose and spread recently. However, both the host and viral polymorphisms are found within multiple natural populations [44–46]. What our data cannot establish is the precise balance of main and interaction variance that would be found in a population sampled at one place and time. Obtaining that estimate would require contemporaneous samples of host and viral genotypes from the same population.

Sex is among the most pervasive sources of variation in infection outcome. Males and females differ in susceptibility, immune signalling and pathology in animals from insects to humans [83,84], and in *Drosophila* these differences are associated with distinct behavioural, epithelial, cellular and systemic defences [84]. Consistent with prior research, we found that the sigma virus is a striking example of this, with strong sex differences in transmission rates [14,30]. This likely results from the virus having different transmission routes in males and females, through ovaries and oocytes versus the male germline and sperm. Infection-induced transcriptional responses also differ between the sexes [85]. Quantifying viral load directly in ovaries, testes, and gametes across host-virus combinations could test whether sex-specific transmission reflects differences in reproductive-tissue infection rather than whole-body viral load. While maternal transmission is the primary route of sigma virus spread, male transmission is essential for viral persistence at the population level: in its absence, the fitness costs of infection would gradually reduce the viral prevalence [33,57,68]. The variance in transmission is similar in both males and females. As there is expected to be selection on the host to reduce transmission to offspring and the virus to increase transmission in both sexes, there are comparable influences of maternal and paternal transmission on fitness variance.

Work on the sexual dimorphism of infection outcomes typically treats sex as a main effect, asking whether one sex is more resistant or has a different immune response. It is less understood whether the specificity of host-pathogen interactions is sex-specific. Our results demonstrate that the identities of the host-pathogen genotype combinations in which infection succeeds differ between the sexes. The host-virus interactions in transmission were only weakly shared across the sexes (cross-sex *r*_*g*_ = 0.34 ± 0.12 SE). Maternal and paternal transmission are therefore two distinct traits with partly separate genetic and mechanistic bases, not one trait expressed twice. This means that selection on one party will not optimise both routes of transmission, because a viral genotype with high transmission in eggs is only weakly predictive of transmission in sperm, and a host genotype that blocks transmission through eggs may not prevent transmission through sperm. This means sex becomes a further source of context dependence alongside partner genotype, dispersing genetic variance still further from the main effects on which directional selection acts.

For fly life-history traits, although G_H_ × G_P_ remained the largest genetic component, the host main effect was of comparable magnitude for fecundity, indicating consistent differences among host lines in their reproductive response to infection. Previous sigma virus studies reported lower egg production in infected flies [33] and reduced egg viability but no significant overall effect on male or female fertility [66], although neither examined combinations of host and viral genotypes. Intriguingly, despite well-documented fitness costs of sigma infection [33,34,39,68,86,87], the reproductive effect of infection was not uniform but depended on the particular host-virus combination and sometimes led to increases in female reproductive output, mirroring weakly supported reports of minor reproductive gains in infected males [66,88]. Whether these apparent increases represent reproducible biological responses to infection, and whether they occur in natural populations, remains unclear.

Transmission tended to be negatively correlated with fecundity and hatch rate, but this phenotypic pattern was not consistently reflected in genetic correlations of either transmission or viral load with fly fitness. We therefore cannot conclude that viral replication underpins a trade-off between virulence and transmission. One possible explanation for this decoupling is tolerance: host genotypes may differ in the reproductive loss they suffer at a given viral burden, so that higher replication need not translate into greater harm. Genetic variation in fecundity tolerance has been documented in insects [20], including *D. melanogaster* infected with *Drosophila* C virus [89]. Under vertical transmission, this could benefit both partners because the virus depends on host reproduction for transmission [71,72]. Tolerance that preserves fecundity therefore also preserves opportunities for viral transmission, potentially weakening selection against otherwise costly infections [55,56]. The generally weak genetic correlations among infection-related traits suggest the G_H_ × G_P_ interactions often have trait-specific effects. This trait-specificity, as observed in *Drosophila* anti-parasitoid responses, argues against the same genetic loci governing different aspects of host-virus interactions [90]. Instead, several independent genetic factors are likely to shape different traits and outcomes [91,92].

We did not aim to dissect the roles of known *Drosophila* resistance genes or sigma infectivity architecture, so we could not isolate individual resistance allele effects. However, although all three known host resistance polymorphisms (*p62*, *CHKov1*, *Ge-1*) were represented in our panel, their effects differed among viral genotypes. While major-effect genes have traditionally been emphasised in studies of host resistance [23], these genotype-specific responses suggest a more complex architecture that cannot be reduced to these loci alone.

Although the concentration of fitness variance in G_H_ × G_P_ may partly reflect features specific to sigma virus, the dynamics and genotype-specific effects that generate this pattern in our models are not restricted to vertically transmitted parasites. For example, temporal studies of *Daphnia*-*Pasteuria* and wild flax-flax rust found reciprocal cycling in host resistance and parasite infectivity through time [93,94], while genomic evidence from *Daphnia* resistance loci indicates that these polymorphisms can persist over long timescales [95]. Fluctuating selection and the maintenance of polymorphisms are therefore common in horizontally transmitted parasites. Furthermore, G_H_ × G_P_ interactions are widespread across many host and parasite traits [6,7]. However, experiments in other systems typically do not decompose variance components for both parties, and studies of horizontally transmitted pathogens often measure traits, such as parasite burden or infectivity, which may not reliably reflect fitness. For example, the burden of the parasite *Ordospora colligata* in *Daphnia* did not reliably predict estimates of fitness from persistence in experimental populations across time [96]. A notable exception is the monarch butterfly-*Ophryocystis* system, where host and parasite genotypes interact to determine virulence and transmission in wild populations (although here the variance was not decomposed into main and interaction components) [6]. In our system, the number of infected offspring provides a simple measure of parasite fitness because the virus is vertically transmitted, and multiple host traits are relatively easily measurable.

The balance of variance components is also contingent on whether coevolution sustains genotype specificity (G_H_ × G_P_) or favours broadly effective resistance and infectivity (main effects). This may be system-specific, with broadly infective phages evolving during bacteria-phage coevolution [97], and general host resistance emerging in the arms race between an alga and its DNA virus [98]. Other features of viral biology may matter too: reassortment or recombination can move compatibility alleles between viral backgrounds, whereas sigma virus is nonrecombining. Therefore, while we would expect our results to hold qualitatively across many other systems, quantitatively, the extent to which the concentration of fitness variance in G_H_ × G_P_ extends beyond this system remains an open and testable question.

Host-parasite coevolution has long been proposed to maintain genetic diversity through genotype-specific fitness effects, yet empirical demonstrations of how these interactions structure variance across different components of fitness have remained rare. Our results show that, in a natural vertically transmitted virus system, genetic variance across a suite of host and virus traits was dominated by the V_G×G_ interaction term, consistent with coevolution masking heritable variation from directional selection while maintaining it through negative frequency-dependent (balancing) selection. This observation aligns with theoretical predictions that NFDS arising from genotype-by-genotype interactions shifts fitness effects from additive effects into interaction components, thereby slowing adaptation and promoting coexistence. Furthermore, G_H_ × G_P_ interactions were frequently sex- and trait-specific, which may further reduce the additive genetic variance in fitness. These results highlight that coevolution can generate extensive cryptic genetic variation, which may be revealed either when ecological or evolutionary conditions shift, or when the process of coevolution itself alters the direction of selection. Our results illustrate the importance of considering genotype-specific interactions when predicting evolutionary responses to emerging pathogens, environmental change, or interventions that alter the distribution of host or parasite genotypes.

## Materials and methods

### Simulation model

We implemented a deterministic model to examine the dynamics of coevolution and how fitness variance is partitioned between additive and interaction components. The basic version of the model, a single pathogen with no genetic variation in either host or parasite, is shown schematically in Fig 9 and specified below. The full model extends this to two co-circulating viral genotypes and two biallelic host resistance loci that coevolve. Simulations were implemented in R (v.4.4.0) in RStudio. The complete derivation of the full model, including the interaction matrices for each of the coevolution regimes considered here, is given in S1 Text.

**Fig 9:**
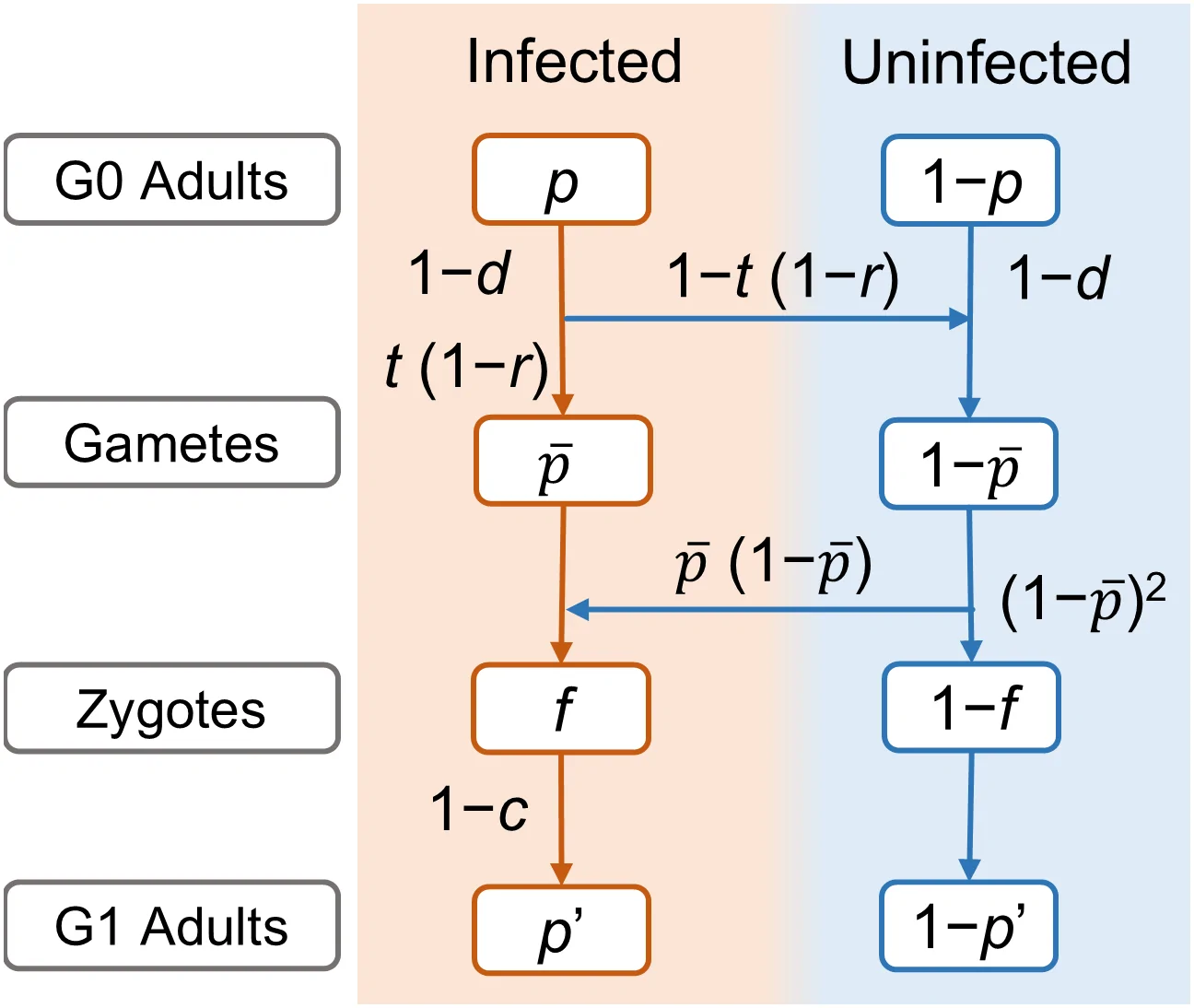
Schematic of the model of a vertically transmitted pathogen. The schematic shows the basic single-pathogen, single-locus version with no genetic variation, corresponding to equations (1)–(3); the full model incorporates two pathogen genotypes and two host resistance loci. The frequencies of infected individuals are *p* among adults, $\bar{p}\;$among gametes, *f* among zygotes, and *p*’ among adults in the next generation. Parameters: *t*, pathogen transmission rate (in *t*he full model *t*ᵢ for virus strain *i*, further reduced to *t*_*ᵢ,G*_ = *t*ᵢ(1 − *r*· 1_*ᵢ,G*_) in hosts carrying a matched resis*t*ance allele); *r*, propor*t*ional reduction in pathogen transmission in hosts carrying a matched dominant resistance allele; *d*, fertility cost per locus at which the host carries a dominant resistance allele; *c*, cost of infection on offspring viability (a single parameter, applied equally to hosts infected with either virus). In the simple model *r* and *d* act uniformly on every host; both become dynamically relevant only when resistance varies across host genotypes in the full model. This figure is schematic and has no underlying numerical data.

The model tracks cohorts of infected (frequency *p*) and uninfected (frequency 1 − *p*) adult hosts across discrete, nonoverlapping generations. Each adult produces gametes, and hosts that carry a matched dominant resistance allele incur a fertility cost *d*, so each such adult’s per-capita gamete output is (1 − *d*). Each infected parent transmits the pathogen to a fraction *t* of its gametes, with equivalent transmission through males and females. Resistance reduces transmission by a proportion *r*, so the effective transmission rate from a matched resistant host is *t(*1 *− r)*. The frequency of infected gametes ($\bar{p}$) is the ratio of infected to total gametes in the population:


$$\begin{array}{c} \bar{p}=\;\frac{p\;(\text{1}\;-\;d)\cdot t(\text{1}-r)\;}{p(\text{1}\;-\;d)\cdot t(\text{1}-r)+\;p(\text{1}\;-\;d)\cdot \left(\text{1}-t(\text{1}-r)\right)+(\text{1}-p)(\text{1}\;-\;d)}=\;p\cdot t\cdot (\text{1}-r) \end{array}$$ (1)

In the simple model, *d* acts uniformly on every adult and so factors out of every term in the gamete pool, leaving $\bar{p}$ to depend only on *p*, *t*, and *r*.

Random union of male and female gametes yields zygotes. A zygote is infected either if the maternal gamete carries the pathogen, or if the maternal gamete is uninfected and the paternal gamete is infected; the resulting infected zygote frequency *f* is


$$\begin{array}{c} f\;=\;\bar{p}+\;\left(\text{1}\;-\;\bar{p}\right)\bar{p}=\;\bar{p}\left(\text{2}\;-\;\bar{p}\right) \end{array}$$ (2)

Infected zygotes develop at a relative viability cost *c*. After applying the viability cost, the frequency of infected adults in the next generation is


$$\begin{array}{c} p^{\prime }=\frac{f(\text{1}\;-\;c)(\text{1}\;-\;d)}{\left[f(\text{1}\;-\;c)(\text{1}\;-\;d)+\;(\text{1}\;-\;f)(\text{1}\;-\;d)\right]}=\frac{f(\text{1}\;-\;c)}{\left[\text{1}\;-\;c\;\cdot \;f\right]} \end{array}$$ (3)

The parameter *d* therefore has no dynamical effect in the simple model because it acts uniformly and cancels during normalisation. The parameter *r* reduces transmission and therefore affects infection prevalence, but it contributes to coevolutionary dynamics only when resistance varies across host genotypes in the full model (S1 Text).

The full model extends equations (1)–(3) by letting resistance and transmission depend on genotype. The infection cost *c* is retained as a single parameter applied equally to hosts infected with either virus, but each viral strain *i* has its own base transmission rate *tᵢ* (*t*_*1*_ and *t*_*2*_ set independently), and *tᵢ* is further reduced in hosts carrying a matched resistance allele, giving the effective transmission rate *t*_*ᵢ,G*_ = *tᵢ*(1 − *r·* 1_*ᵢ,G*_), where 1_*ᵢ,G*_ is a resistance indicator (1 if host *G* is resistant to virus *i*, 0 otherwise). The fertility cost *d* is then applied at the gamete stage, multiplicatively per locus at which the host carries a dominant resistance allele, so host genotypes now differ in their gamete output (a host resistant at both loci pays (1 − *d*)²). The difference *t*_*1*_ − *t*_*2*_ represents an infectivity cost of escape from host resistance when *t*_*2*_ *< t*_*1*_—*t*he parasite-side counterpart of the host’s fertility cost *d*.

At each generation, fitness variance across the joint host-virus genotype distribution is decomposed into host-genotype, virus-genotype, and host × virus interaction components, for both host and virus fitness (S1 Text).

The simulations track the frequencies of host genotypes and their infection status across generations. The full model maintains a two-locus structure with two biallelic resistance loci (*A/a* and *B/b*) and two viral genotypes, coevolving the nine possible diploid host genotypes with the two viruses. Three coevolution models were implemented (S1 Fig). In the single-locus gene-for-gene models (GFG1), shown in the main text, dominant allele *A* confers resistance to Virus 1 and gene *B* is absent. In the two-locus gene-for-gene model (GFG2), a dominant allele *B* of a second unlinked host gene confers resistance to Virus 2. In the matching-allele model (MA), allele *A* confers resistance to Virus 1 while the susceptible genotype (*aa*) confers resistance to Virus 2—the same locus determines resistance to both viruses, in opposite directions depending on genotype. A selective sweep model was additionally implemented. This is identical to GFG2, but with no cost of resistance and with the sequential introduction of Virus 2 and allele *B* at fixed generations, simulating directional arms-race dynamics. Full model mechanics and interaction matrices are provided in S1 Text.

### Parameter sweep

To characterise the parameter space supporting different coevolutionary outcomes, we systematically varied key parameters of the GFG1 model across biologically plausible ranges informed by field estimates from natural *Drosophila*-sigma virus populations, with extended ranges to explore model behaviour outside the empirically observed window. For each parameter combination, we recorded mean resistance-allele and virus-genotype frequencies, the fitness variance components *V*_*G(host)*_, *V*_*G(virus)*_*,* and *V*_*G×G*_, and the qualitative type of coevolutionary dynamics. Full details of the parameter-sweep procedure are given in S1 Text.

### Fly lines

We used nine *D. melanogaster* lines from the DGRP [67], six of which were kindly provided by Pedro Vale, University of Edinburgh. These lines were selected based on fitness scores [99] and variants of genes known to confer resistance to DMelSV (*Ge-1*, *CHKov1*, *p62* (*ref2p*)) [41–43,75,100]. Line 153 carries resistance alleles at all three genes; line 361 carries a resistance allele at *Ge-1* and is heterozygous at *CHKov1*; line 21 carries a resistance allele at *CHKov1* only; line 776 carries a resistance allele at *p62* only; and lines 303, 443, 712, 801, and 850 carry susceptible alleles at all three genes. We confirmed the alleles by PCR with primers targeting susceptible and resistant alleles [23].

### Virus isolates

Ten sigma virus isolates from geographically diverse *D. melanogaster* populations were used, including publicly available Ap30 – Florida, United States; PF107 – Athens, Greece; GC020 – Galicia, Spain; E320 – Essex, UK [46,101]; and strains collected from the wild fly populations during summer 2021 in the United Kingdom: Me44 – Cambridgeshire; Ed36 – Edinburgh; Gr6 – Sussex; Au45 and Au115 – Perth and Kinross; and Ch384 – Cambridge. To identify unique genotypes of the viral isolates from the same collection locations and to confirm the correct genotypes of the publicly available isolates, we sequenced part of the viral genome covering the region from gene P to the beginning of gene L by Sanger sequencing. The sequences were deposited in GenBank under accession numbers PZ436669–PZ436678. Sequences were aligned using MUSCLE and then trimmed by eye to remove regions with missing data [102]. Phylogenetic reconstruction was performed using BEAST v1.10.4 with 6,332 bp of sequences fitted to an uncorrelated relaxed lognormal molecular clock model using a speciation birth-death process tree-shape prior [103]. An HKY substitution model with a gamma distribution of rate variation with four categories and a proportion of invariable sites was used. Models were run for 100,000,000 steps using a 10% burn-in. Sequence alignment was also used for designing qRT-PCR primers (Table E in S1 Text).

### Generation of infected lines

Infected host-virus lines were established by pairing each of the nine host lines with each of the 10 viral isolates. Viral isolates were introduced into DGRP host genetic backgrounds using a backcross scheme adapted from Wilfert and Jiggins (2010) [46], using the translocation balancer stock *T(2;3)*CyO-TM8 (CyO, *Adh*^*nB*^: TM8, *l*^*3*^*DTS4*^*1*^, Bloomington). Balancer chromosomes carry dominant visible markers and suppress recombination, allowing specific chromosomes to be tracked through crosses. The translocation between chromosomes 2 and 3 makes the two main autosomes segregate in the progeny of the cross as if they were a single chromosome, simplifying the genetic crosses. We used the balancer stock to introduce each virus isolate into the intended DGRP genetic background in a controlled way and to confirm the resulting genotypes. For published viral isolates, infection was first established in *w*^1118^ (DrosDel) [104] adults by intra-abdominal injection of crude homogenates prepared from infected fly tissue, following standard sigma virus injection protocols [105]. Once infection was established, infected *w*^1118^ females were crossed to males of the balancer stock (Generation 1 cross: *w*^*1118*^ ♀ × *T(2;3)CyO*-*TM8* ♂). Infected F1 females carrying *T(2;3)CyO*-*TM8* were crossed to males of the target DGRP line (Generation 2 cross: F1 infected *T(2;3)CyO*-*TM8* / *w*^*1118*^ ♀ × DGRP ♂). From F2 offspring, infected siblings carrying the balancer chromosome were crossed to establish stabilised infected stocks (Generation 3 cross: F2 infected *T(2;3)CyO*-*TM8* / DGRP ♀ × F2 infected *T(2;3)CyO*-*TM8* / DGRP ♂). Stocks were then continued by selecting infected offspring not carrying the balancer chromosome. Established stocks were maintained for two or more generations until infections were stabilised before phenotypic assays were performed. After two generations of backcrossing, infected lines were considered to carry the autosomal genetic background of the recipient DGRP line. For wild-caught viral isolates, infected females collected from natural populations were crossed directly to males of the balancer stock as the Generation 1 cross (infected wild-caught ♀ × *w*^*1118*^; *T(2;3)CyO*-*TM8* ♂). All subsequent crosses proceeded identically to those for the infected *w*^*1118*^ isolates. Sigma virus infection status was confirmed at each generation selection step by CO_2_ sensitivity assay, in which actively infected flies exhibit prolonged paralysis upon exposure to CO_2_ gas [37].

### Experimental design

All assays were conducted using a randomised block design. Given the scale of the experiment (90 infected fly stocks plus uninfected controls), assays were completed across several rounds with 10–30 lines per round. Approximately one-quarter of lines from each previous round were carried forward as internal replicates to allow correction for between-round variation. The number of DGRP lines per round was restricted to 2–3 to minimise the number of uninfected controls required. We maintained fly stocks and conducted all phenotypic tests at 25 °C with 65% humidity and a 12:12 hour light:dark photoperiod. For all phenotypic tests, to stabilise sigma virus infection (transmission rates are higher through offspring infected by their mothers) [37], infected parental flies used in tests were the F1 offspring of one infected female crossed to 2–3 males of the corresponding uninfected DGRP line.

### Viral transmission test

We tested the transmission rate of the different virus isolates in each host genetic background. Transmission and reproductive-trait assays were performed using the randomised block design described above. For the transmission test, we used individual virgin 2–3-day-old infected parental females or males from each of the 90 infected lines and a single uninfected fly of the opposite sex of the same age. Flies were kept in vials with cornmeal medium [23] supplemented with dried yeast for four days for egg laying. Next, 6–7-day-old parents were collected and tested for infection and immediately homogenised in 180 µL TRIzol (Invitrogen, USA) and stored at −80 °C for subsequent RNA extraction. The total number of the offspring and the number of infected flies were counted for each of the infected parents. Infection was scored using the standard CO_2_ assay, in which infected flies are paralysed by exposure to CO_2_. A minimum of two biological replicates were used for grandparental flies and three for the parental flies for each infected line and sex tested.

### Virulence test

The impact of sigma infection on female reproductive performance was assessed by measuring changes in fecundity (number of eggs laid) and hatch rate (proportion of eggs hatched) in both infected and uninfected flies. The virulence test across all 90 infected lines was conducted following the same randomised block design as described above. For the control, we used uninfected DGRP lines, and all tests were performed in the same way as for the infected flies. For the tests, individual infected virgin females (parental test flies) were crossed with an uninfected male of the same age from the corresponding DGRP lines. For most lines, a minimum of two biological replicates for progenitor flies and two for parent flies were used for each infected line.

Flies were placed on yeasted cornmeal medium, then after four days transferred to vials containing apple-juice agar (2% agar) with 30 µL of yeast solution (1 mg live yeast in 3 mL H_2_O). After two further days, flies were transferred to vials with fresh apple-agar medium containing 0.5% activated charcoal (to provide contrast for counting) and 3 µL of yeast solution (1 mg dry yeast in 10 mL H_2_O) for 24 hours. The surface of the medium was brushed to create an optimal surface for oviposition. The number of eggs laid per vial was counted for infected and control flies. For the fertility test, 2 days after the fecundity test, hatched eggs were counted. Hatch rate was calculated as hatched eggs divided by total eggs laid and was recorded as missing when no eggs were laid. The number of eggs hatched was also used as a measure of larval offspring. To account for variation in baseline fitness across DGRP lines, relative fecundity, relative hatch rate, and relative larval offspring were calculated by dividing each infected-fly value by the mean for uninfected control flies of the same DGRP line.

### Measurement of viral RNA load

We measured the viral load in 6–7-day-old adult flies used in the transmission test for all combinations of viral and host genotypes and both sexes. For each infected fly line and sex, up to 14 randomly selected individual flies, homogenised in TRIzol, derived from up to six grandparental flies (see viral transmission test), were proportionally pooled in one sample for viral RNA extraction. RNA was extracted following the standard TRIzol extraction protocol.

In total, two pooled RNA samples were obtained for most infected lines and both sexes. We reverse transcribed RNA with GoScript reverse transcriptase (Promega, USA) and random hexamer primers. Viral RNA was quantified by qRT-PCR relative to the endogenous control housekeeping gene *RpL32* in two technical replicates, using primers from Longdon and colleagues [106]. The viral RNA was amplified using primers targeting sequence common for all virus genotypes overlapping viral genes *X-M* (Table E in S1 Text). We performed qRT-PCR using cDNA diluted 1:10 with nuclease-free water on the Applied Biosystems StepOnePlus system. The reaction was done with SensiFAST SYBR Hi-ROX (Meridian Bioscience, USA) and the following PCR cycle: 50 °C for 2 min followed by 95 °C for 10 min and followed by 40 cycles of: 95 °C for 15 s followed by 60 °C for 60 s. Each technical replicate for either housekeeping or viral primers for all samples was placed on one 384-well plate. Samples were excluded when the Ct difference between technical replicates exceeded 1.8 for either *RpL32* or the viral target.

A large sex-specific difference in *RpL32* expression was detected, requiring normalisation that accounted for sex. This difference is expected in *D. melanogaster*: *RpL32* (*Rp49*) is normally expressed more highly in females than males (FlyAtlas 2; [107]). Normalising within each sex removes this baseline difference from the viral load estimates. *RpL32* Ct values were mean-centred within each host genotype × virus genotype × sex combination, and the overall mean *RpL32* Ct was added back to retain the original Ct scale and allow comparison with the sigma virus Ct values. Normalised ΔCt, representing relative viral RNA load, was calculated as the mean-centred *RpL32* Ct minus the Ct of the targeted sigma virus gene. Relative viral RNA load corresponds to log_2_ viral load.

### Statistical analysis

We used linear mixed models, fitted with ASReml-R to estimate genetic variance components for viral load, transmission rate, fecundity and hatching rate [108]. Given strong evidence of sexual dimorphism in viral load and transmission we modelled these as sex-specific traits in order to detect any genotype×sex interactions present. First, we fitted a univariate model to each of the six traits, with host identity, virus identity, and their interaction fitted as random effects. Viral load and transmission models had no fixed effects. For fecundity and hatch rate, the line-specific mean of uninfected flies was fitted as a fixed covariate and only infected flies were included as responses. This yielded estimates of the host and virus main-effect variances, and variance attributable to G_H_ × G_P_ interactions. We scaled all three variance components to intra-class correlations (i.e., proportions of total phenotypic variance). We repeated the transmission and viral load models using two restricted datasets: first, only the five DGRP lines susceptible at all three known resistance loci (303, 443, 712, 801, and 850), retaining all 10 viral isolates; and second, all nine DGRP lines but only the seven viral isolates collected in the UK (E320, Me44, Ed36, Gr6, Au45, Au115, and Ch384). For each trait, we tested the significance of the three genetic variance components using likelihood ratio tests (assuming a 50:50 mix of *χ*^*2*^ distributions with zero and one degree of freedom following [109]). All models assumed Gaussian error structures. Variance components were constrained to be positive and, in some instances, estimates were bound to the edge of allowable parameter space. In such cases, we interpret the point estimate variance as zero (with no standard error estimable).

Next, we formulated a multivariate mixed model to estimate among-trait genetic correlations at host, virus and host×virus levels. Fixed and random effects were as described for the univariate models. Random effect structures were modelled using generalised heterogeneous correlation matrices in ASReml-R, while a diagonal matrix (i.e., trait-specific variances but no covariances) was used for the residual structure. This is because different traits were measured on different flies (so observation-level residual covariance is not estimable). Traits were scaled to standard deviation units to facilitate model fitting, but despite this we could not obtain a stable convergence for the full six-trait model. Consequently, results are from a five-trait model after dropping hatch rate. Genetic correlations with hatch rate and the other traits were then estimated using a series of bivariate models. The phenotypic correlations among virulence, transmission, and viral load were estimated by Pearson’s product-moment correlation.

Additionally, we tested whether closely related viruses had similar patterns of host-specific effects across fly lines. For transmission and viral load in each sex, we subtracted each virus’s mean across fly lines from its value in every line, removing the virus main effect. We then calculated Euclidean distances between these sets of deviations for each pair of viruses and compared them with phylogenetic distances, measured as the summed branch lengths between each pair of viruses, using one-sided Mantel tests with 9,999 permutations. We also adjusted *p* values across the four tests using Holm’s method.

### Use of artificial intelligence tools

ChatGPT (OpenAI) and Claude (Anthropic) were used during manuscript revision to assist with language editing, preparation and checking of R scripts, consistency checks across the manuscript and supporting files, and the graphical design of the schematic in Fig 1. All outputs were reviewed and edited by the authors, and numerical results were checked against the underlying data and original analyses. The authors determined all scientific interpretations and conclusions and take full responsibility for the final manuscript. AI tools were not used to generate primary data.

## Supporting information

S1 Text Supplementary methods, full description of the coevolutionary simulation model, and supplementary statistical results. S1.1 Full coevolution model. S1.2 Tables. Supplementary statistical results. Includes Tables A–I: host resistance indicators used in the simulation models; genetic variance components from the full and restricted-line analyses; genetic correlations from multivariate and bivariate models; and primers used in this study.
(DOCX)

S1 Data Experimental data. Excel workbook containing the experimental data underlying Figs 4–8 and S5. Sheets contain summary statistics for each host–virus genotype combination, raw qRT-PCR data used to quantify viral load, raw transmission data, raw reproductive-fitness data, and descriptions of all columns.
(XLSX)

S2 Data Simulation data. Excel workbook containing parameter-sweep results and per-generation time series underlying Figs 2 and 3 and S2–S4, S6–S8. Separate sheets contain the original, pre-cost and post-cost virus-loss analyses shown in S8 Fig. The Data_descriptions sheet defines the recorded variables, filters and summary statistics.
(XLSX)

S3 Data Sequence alignment and phylogenetic tree used in Fig 4. The archive contains the trimmed FASTA alignment and annotated NEXUS tree. (ZIP)

S1 Fig Host-virus infection matrices for the three interaction models. The matrices show the effective vertical-transmission rate for each host-virus genotype combination under the matching-allele, one-locus gene-for-gene, and two-locus gene-for-gene models. Susceptible combinations have transmission rate tᵢ, whereas matched resistant combinations have transmission rate *tᵢ*(1 − *r*). Resistance is dominant. Parameters are defined in Fig 9. This figure is schematic and has no underlying numerical data.
(TIF)

S2 Fig Coevolutionary dynamics under a matching-allele model (MA). Time series of allele frequencies and genetic variance components under damped (left) and persistent (right) oscillation regimes. The host carries one biallelic resistance locus where allele *A* confers resistance to Virus 1 and homozygous recessive aa confers resistance to Virus 2. The data supporting this figure can be found in S2 Data.
(TIF)

S3 Fig Coevolutionary dynamics under a two-locus gene-for-gene model (GFG2). Time series of allele frequencies and genetic variance components under damped (left) and persistent (right) oscillation regimes. The host carries two unlinked biallelic resistance loci (*A/a* and *B/b*); allele *A* confers resistance to Virus 1 and allele *B* to Virus 2. The data supporting this figure can be found in S2 Data.
(TIF)

S4 Fig Selective sweep dynamics in the GFG2 model with cost-free resistance (*d* = 0). Resistance alleles undergo sequential selective sweeps rather than persistent oscillations. Virus 1 and resistance allele *A* were initially present at frequencies of 0.0001. Virus 2 was introduced at generation 600 and resistance allele *B* at generation 700, each at a frequency of 0.0001. Parameters were *t*_*1*_ = 0.73, *t*_*2*_ = 0.70, *c* = 0.23, *r* = 0.27, and *d* = 0; therefore, the escaping viral genotype had a transmission cost of 0.03. (A) Frequencies of viral genotypes and resistance alleles. (B) Host-fitness variance components. (C) Viral-fitness variance components. The data supporting this figure can be found in S2 Data.
(TIF)

S5 Fig Relationships between viral transmission and host reproductive traits. Scatter plots show transmission rate against relative hatch rate, relative eggs laid, and relative larval offspring, with the comparisons shown separately by sex. The underlying data are provided in S1 Data.
(TIF)

S6 Fig Parameter-space summaries for *r* = 0.27. Multi-page figure showing (A) the qualitative coevolutionary outcome; (B–D) coefficients of variation in the frequencies of Virus 1, Virus 2, and resistance allele *A*; (E–G) their mean frequencies; (H and I) host-by-virus interaction variance for host and virus fitness; and (J and K) the host and virus additive proportions. Each page shows results across infection cost *c* and resistance cost *d* for the transmission-rate pairs indicated in the panel headers. Grey cells in panels B–K indicate parameter combinations for which no tail-window generations passed the prevalence filter. The data supporting this figure can be found in S2 Data.
(PDF)

S7 Fig Parameter-space summaries for r = 0.70. Multi-page figure showing (A) the qualitative coevolutionary outcome; (B–D) coefficients of variation in the frequencies of Virus 1, Virus 2, and resistance allele *A*; (E–G) their mean frequencies; (H and I) host-by-virus interaction variance for host and virus fitness; and (J and K) the host and virus additive proportions. Each page shows results across infection cost *c* and resistance cost *d* for the transmission-rate pairs indicated in the panel headers. Grey cells in panels B–K indicate parameter combinations for which no tail-window generations passed the prevalence filter. The data supporting this figure can be found in S2 Data.
(PDF)

S8 Fig Effect of the timing of virus loss on coevolutionary dynamics. The gene-for-gene parameter sweep of Fig 3 was repeated with virus loss occurring (A) during transmission to gametes, as in the original model; (B) after fertilisation but before the viability cost of infection; or (C) after the viability cost. The top row shows the position of virus loss in the life cycle and the bottom row shows the mean coefficient of variation of viral-genotype frequencies. Loss before the viability cost produced stronger damping, whereas loss after the cost produced results similar to the original model. Persistent oscillations remained possible under all three timings. Grey cells indicate loss of the virus or resistance allele. The data supporting this figure can be found in S2 Data.
(TIF)

## Abbreviations

CV: coefficient of variation
DGRP: Drosophila Genetic Reference Panel
GFG1: gene-for-gene model
GFG2: gene-for-gene model
NFDS: negative frequency-dependent selection.
