# Supplementary material for: Host–virus interactions dominate fitness and conceal additive genetic variation
Source: PLoS Biol. 2026 Oct 22;24(10):e3003836. doi: 10.1371/journal.pbio.3003836 (PMC13690250; doi:10.1371/journal.pbio.3003836)
Supplement: Supplementary file 1 [file pbio.3003836.s010.pdf]

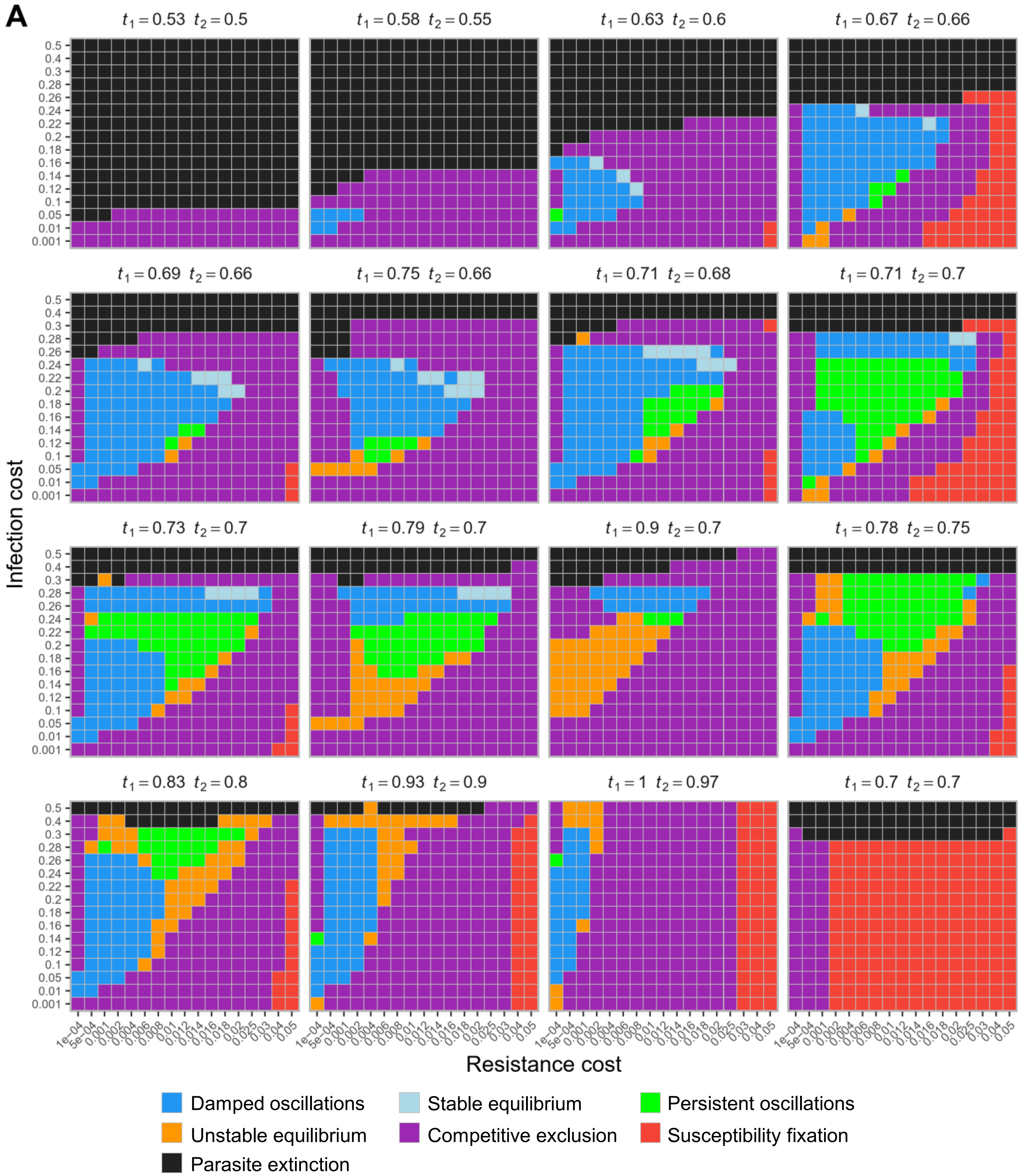

S6A Fig. Qualitative outcome across parameter space for *r* = 0.27. Each cell shows the classified coevolutionary outcome (stable equilibrium, damped oscillations, persistent oscillations, unstable equilibrium, competitive exclusion, susceptibility fixation, or parasite extinction) for a given combination of infection cost c (vertical axis) and resistance cost d (horizontal axis). Each panel corresponds to one (t_1, t_2) transmission-rate pair as indicated in the panel header. The data supporting this figure can be found in S2 Data.

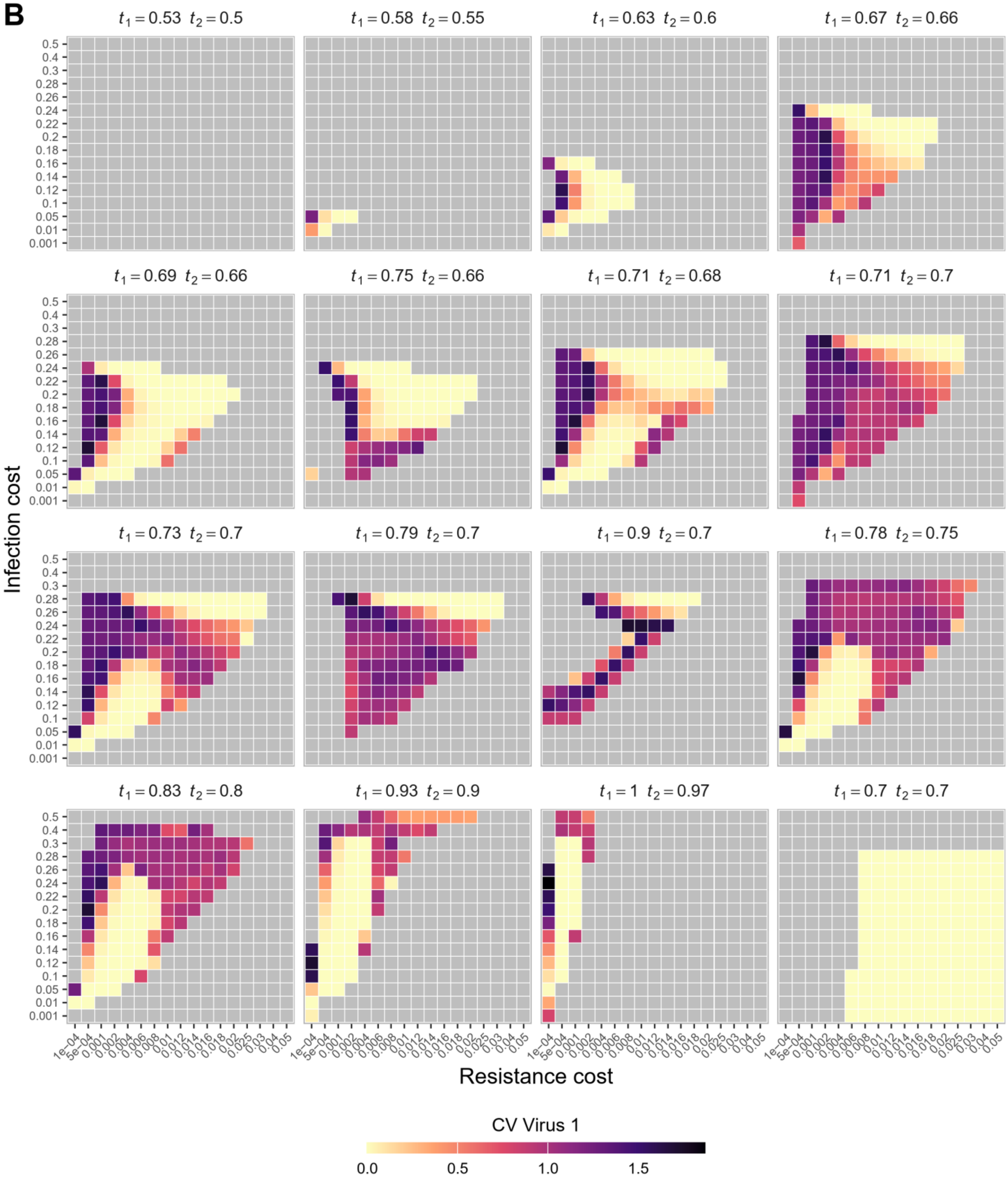

S6B Fig. Coefficient of variation (CV = SD/mean) of Virus 1 frequency across the tail window, for *r* = 0.27. Higher CV indicates larger oscillation amplitude. Here and below grey cells indicate parameter combinations where no tail-window generations passed the prevalence filter. The data supporting this figure can be found in S2 Data.

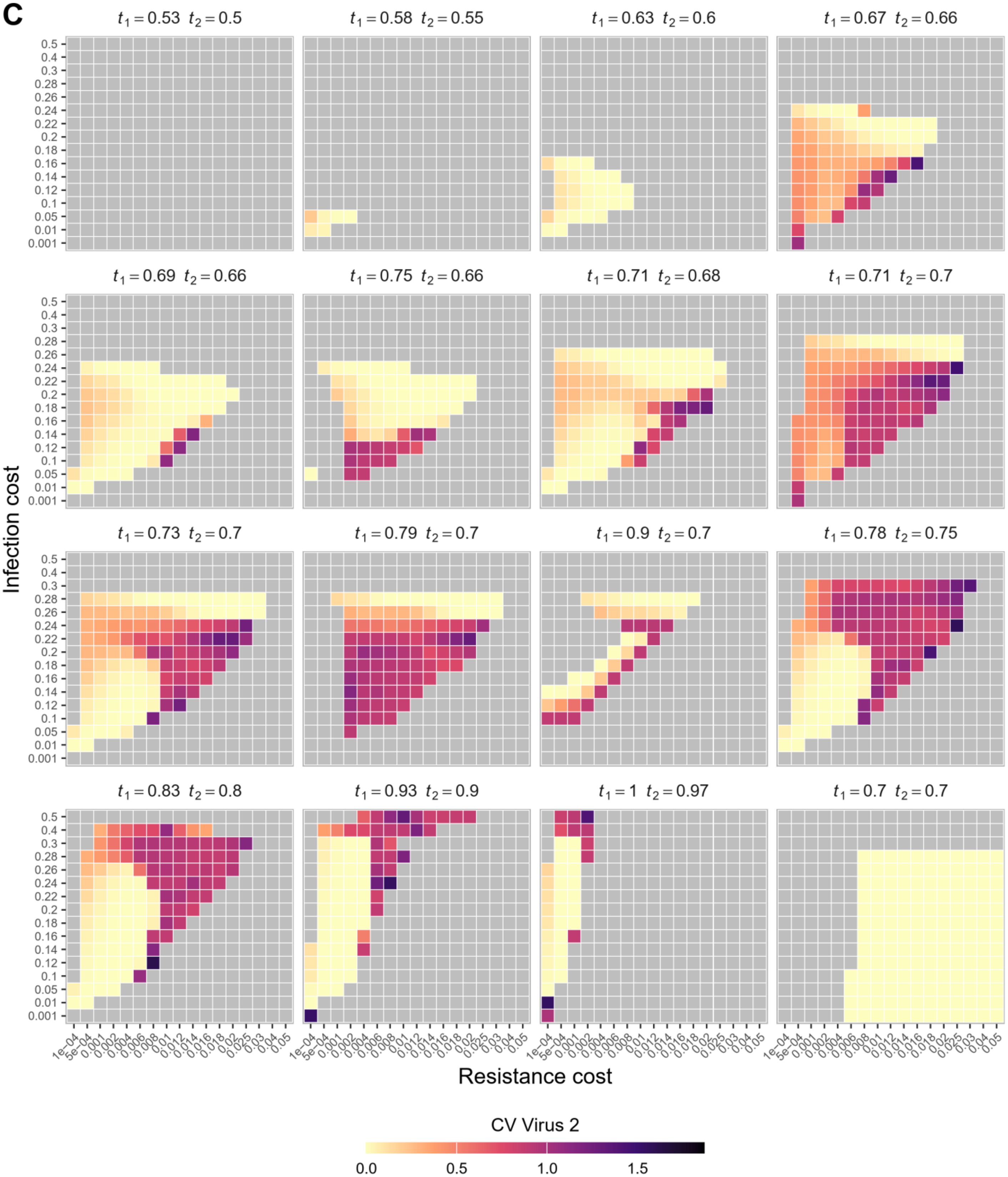

S6C Fig. Coefficient of variation of Virus 2 frequency, for *r* = 0.27. Higher CV indicates larger oscillation amplitude. The data supporting this figure can be found in S2 Data.

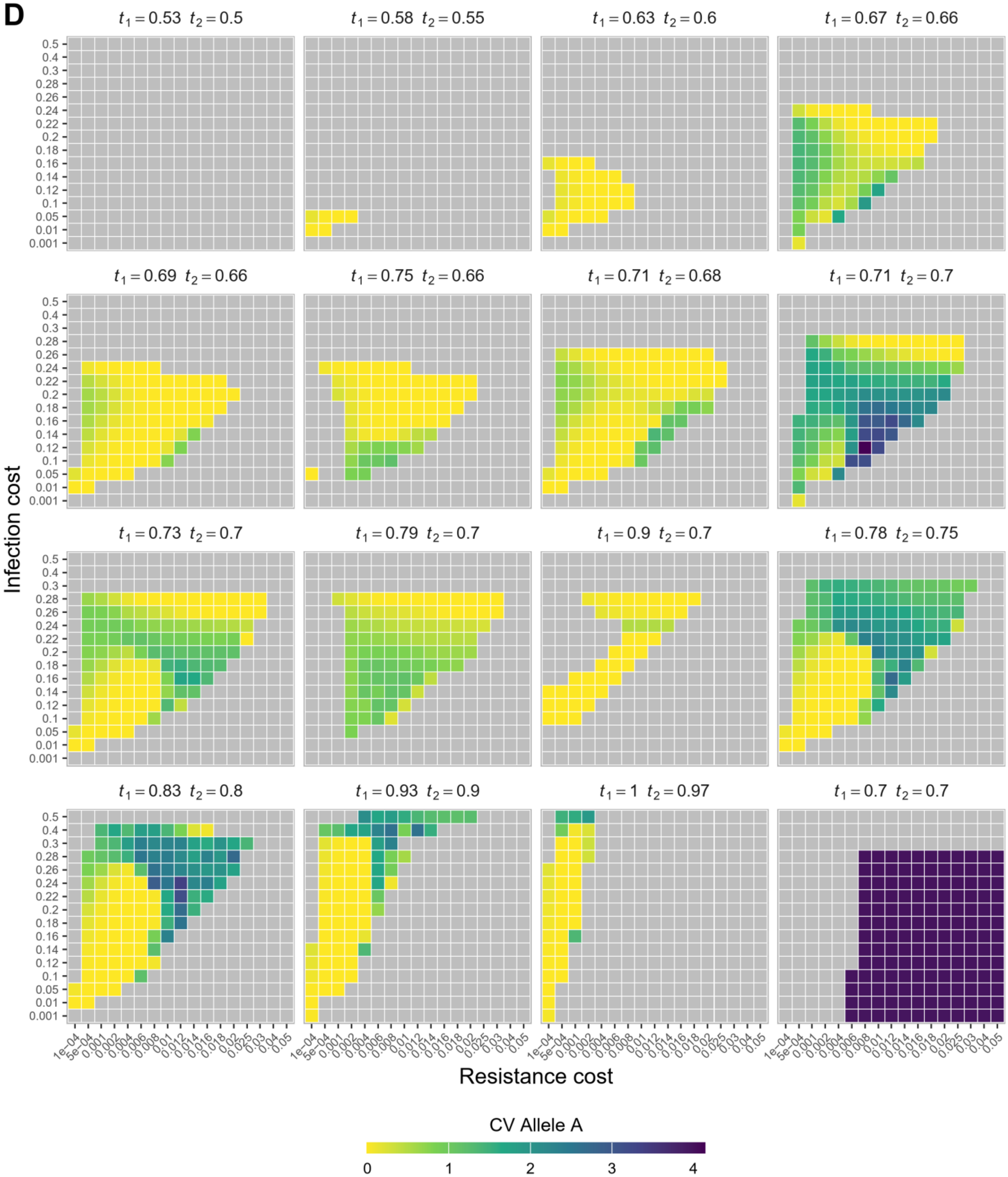

S6D Fig. Coefficient of variation of resistance allele *A* frequency across the tail window, for *r* = 0.27. Higher CV reflects greater fluctuation in host allele frequencies, consistent with ongoing coevolutionary cycling. The data supporting this figure can be found in S2 Data.

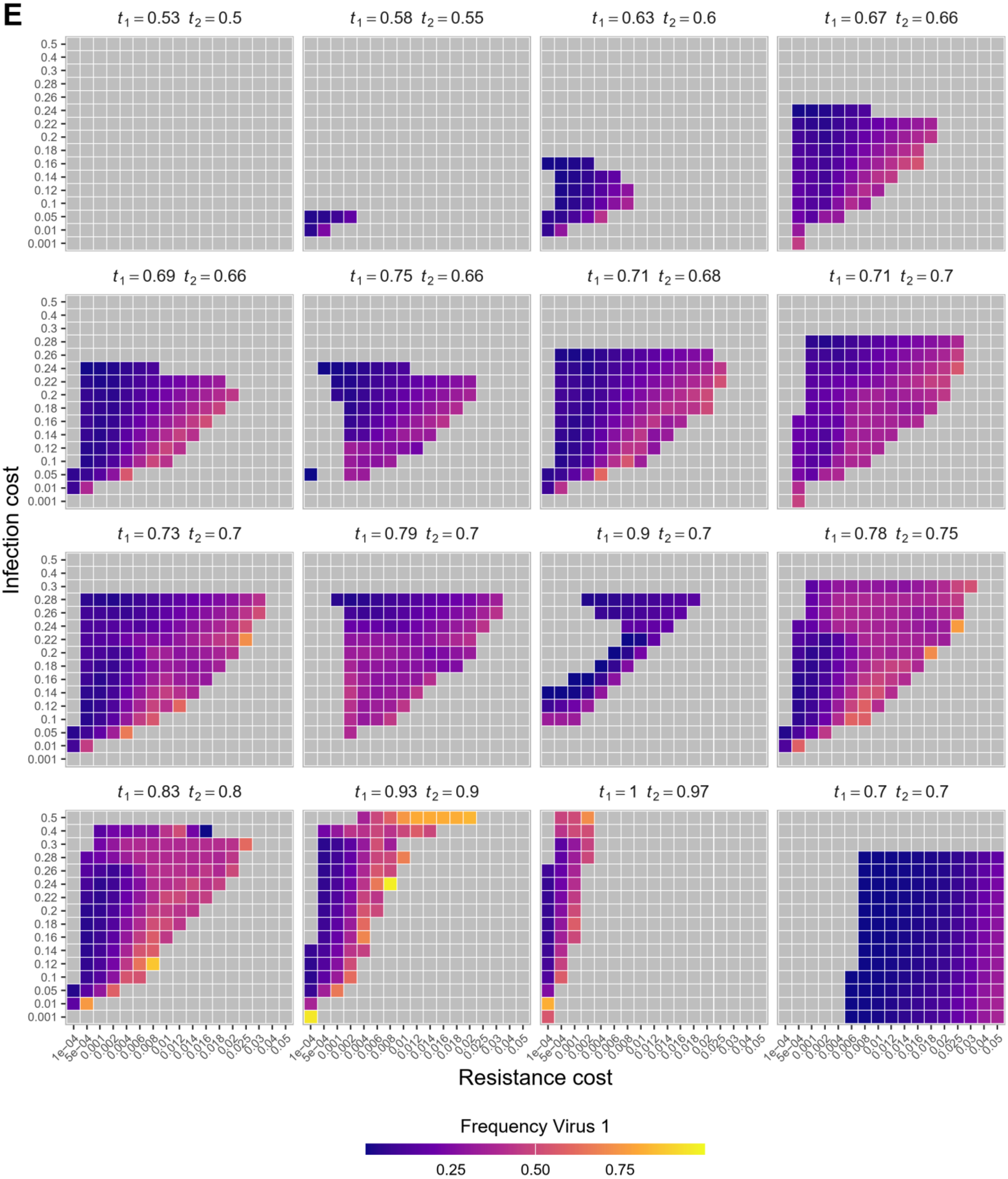

S6E Fig. Mean Virus 1 frequency, for *r* = 0.27. The data supporting this figure can be found in S2 Data.

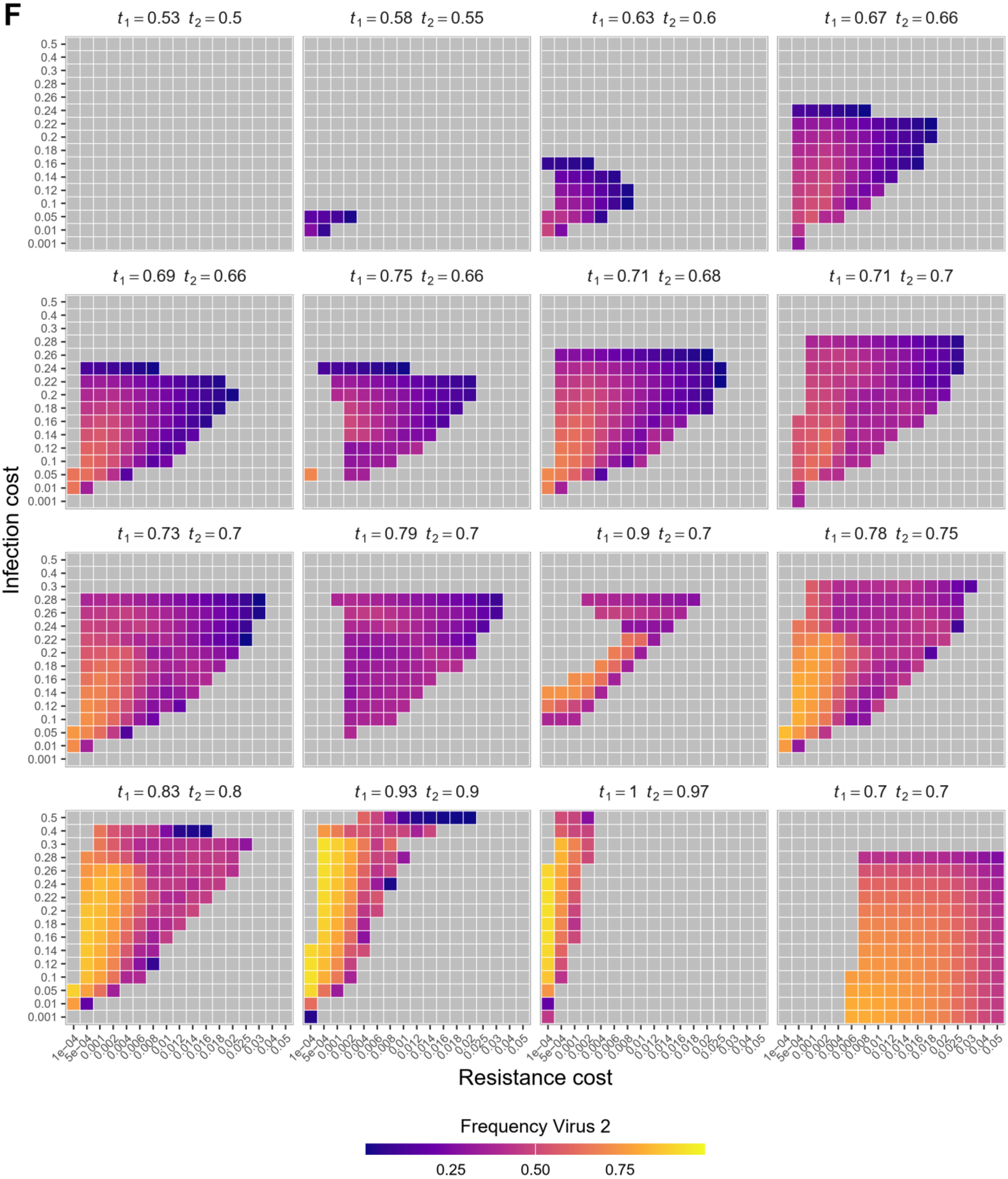

S6F Fig. Mean Virus 2 frequency, for r = 0.27. The data supporting this figure can be found in S2 Data.

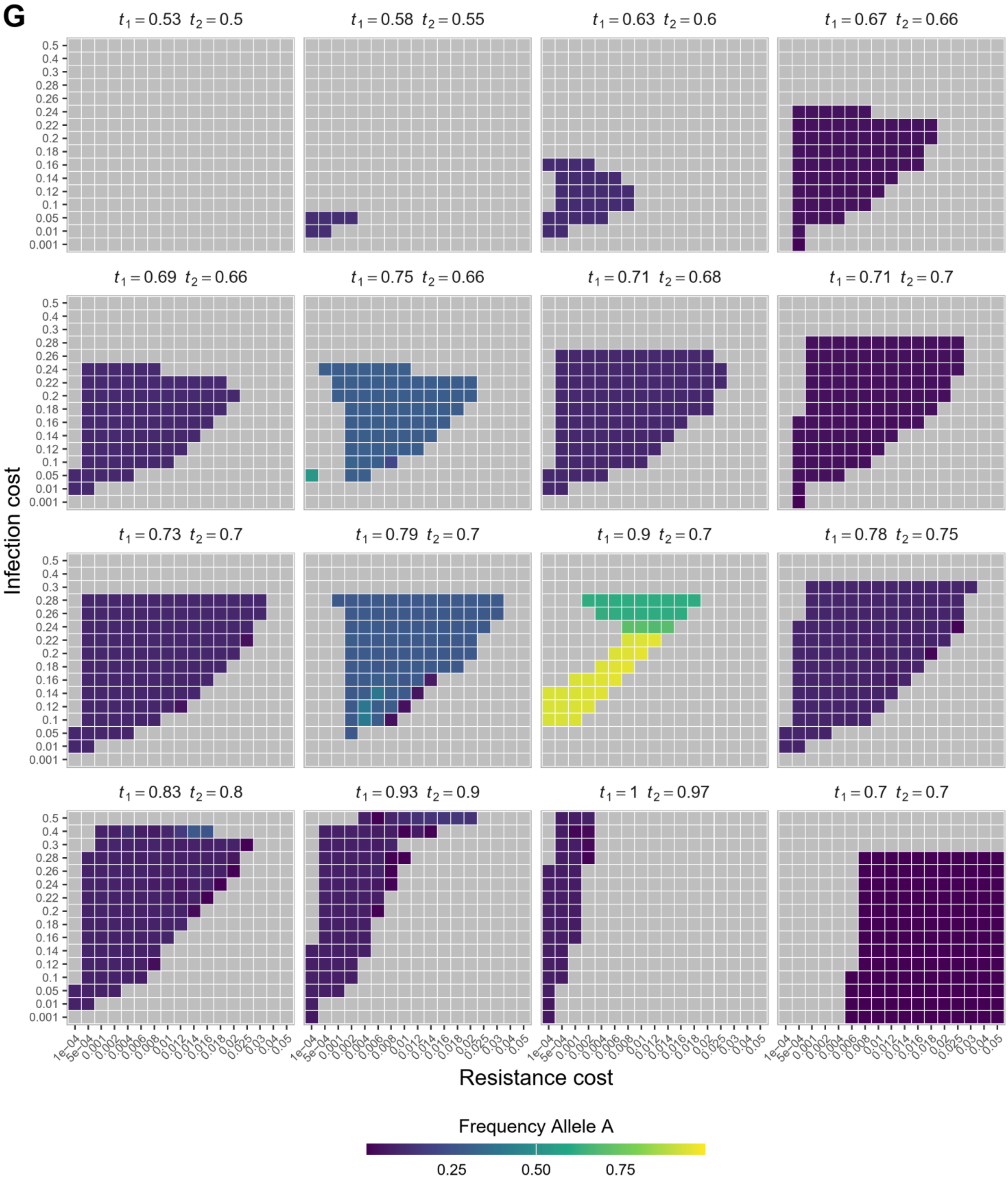

S6G Fig. Mean resistance allele A frequency, for *r* = 0.27. The data supporting this figure can be found in S2 Data.

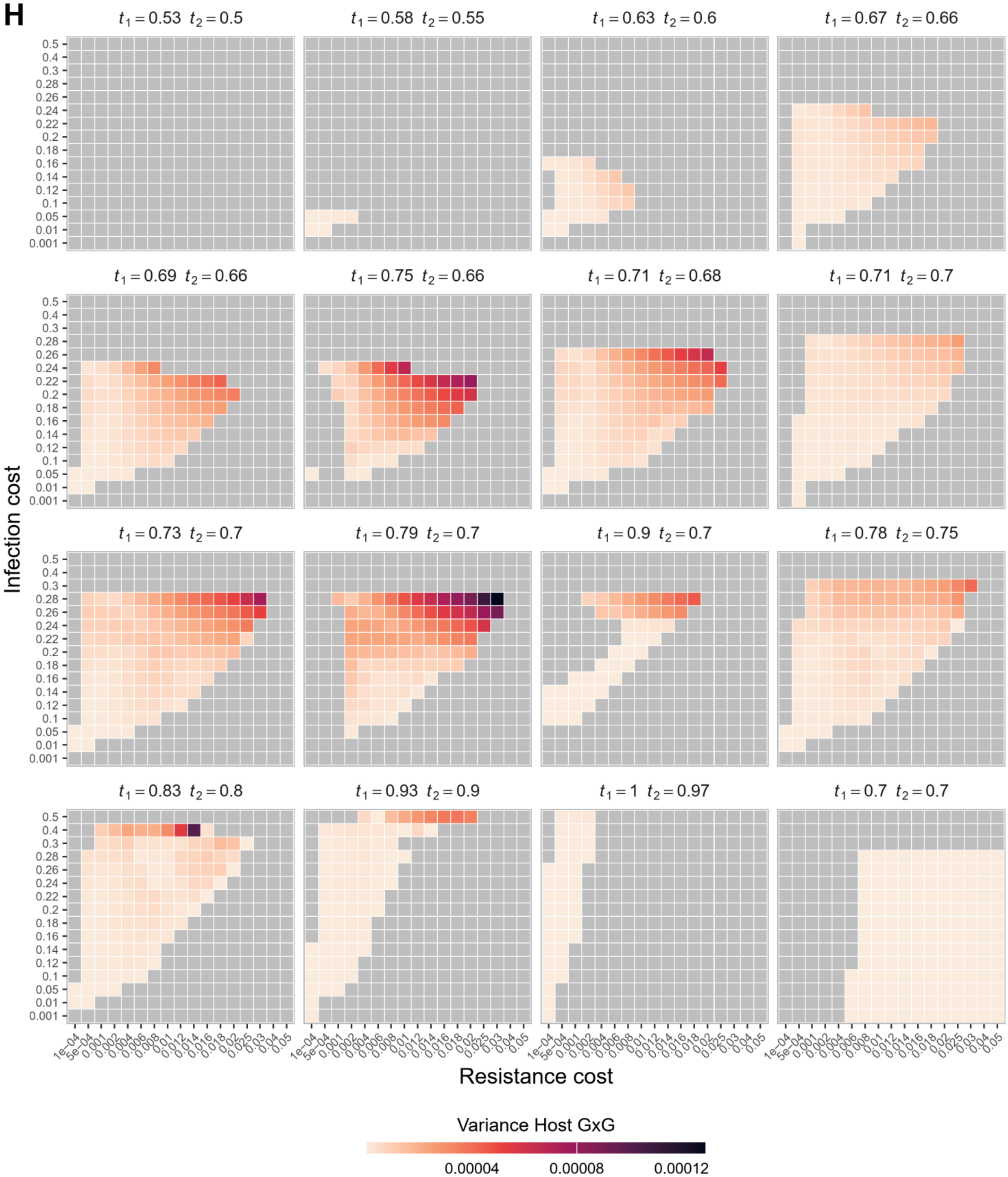

S6H Fig. Variance in host fitness attributable to the host × virus genotype interaction, for *r* = 0.27. Higher values indicate greater variance in host fitness attributable to specific host–virus genotype combinations. The data supporting this figure can be found in S2 Data.

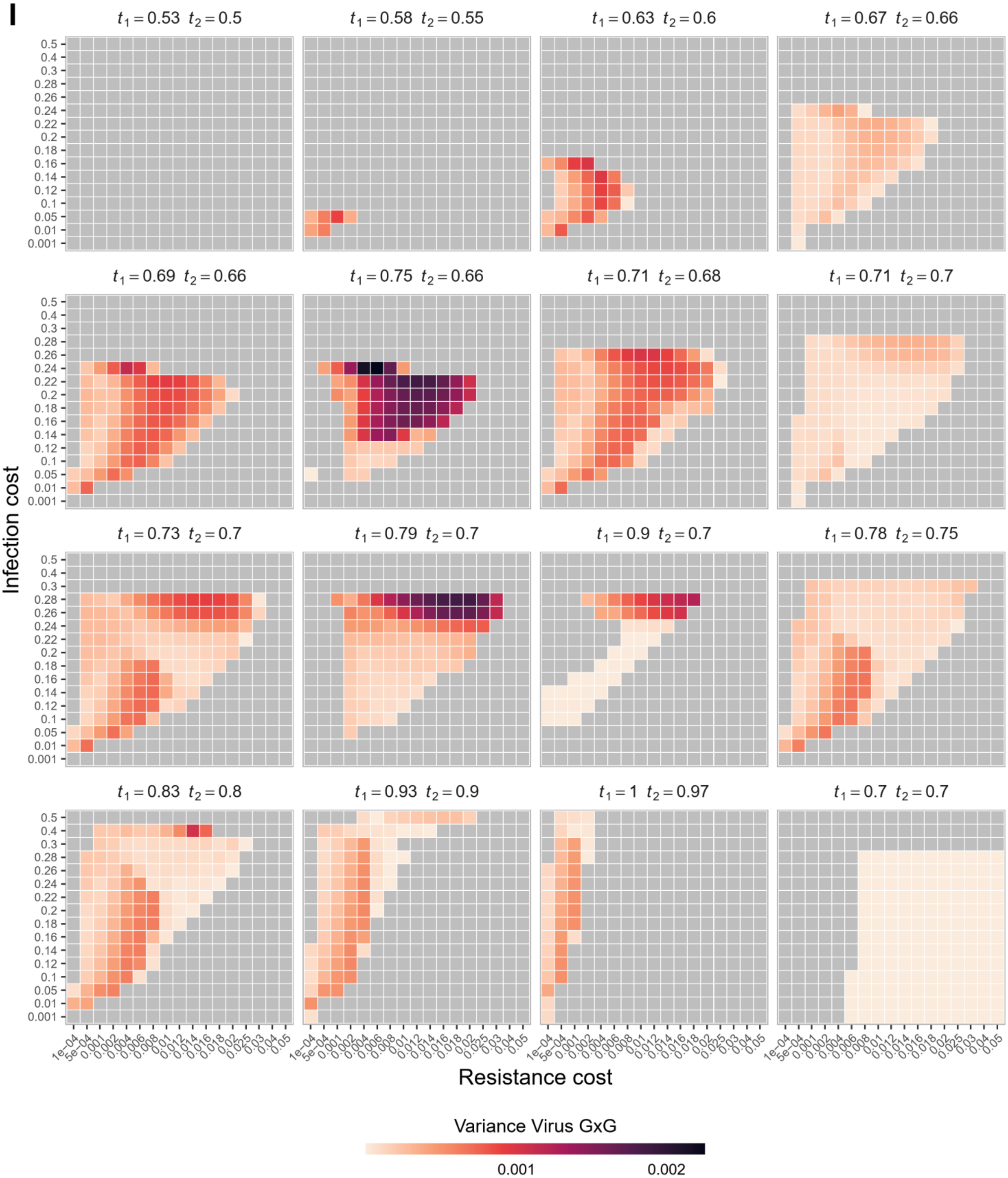

S6I Fig. Variance in virus fitness attributable to the host × virus genotype interaction, for *r* = 0.27. Higher values indicate greater variance in virus fitness attributable to specific host–virus genotype combinations. The data supporting this figure can be found in S2 Data.

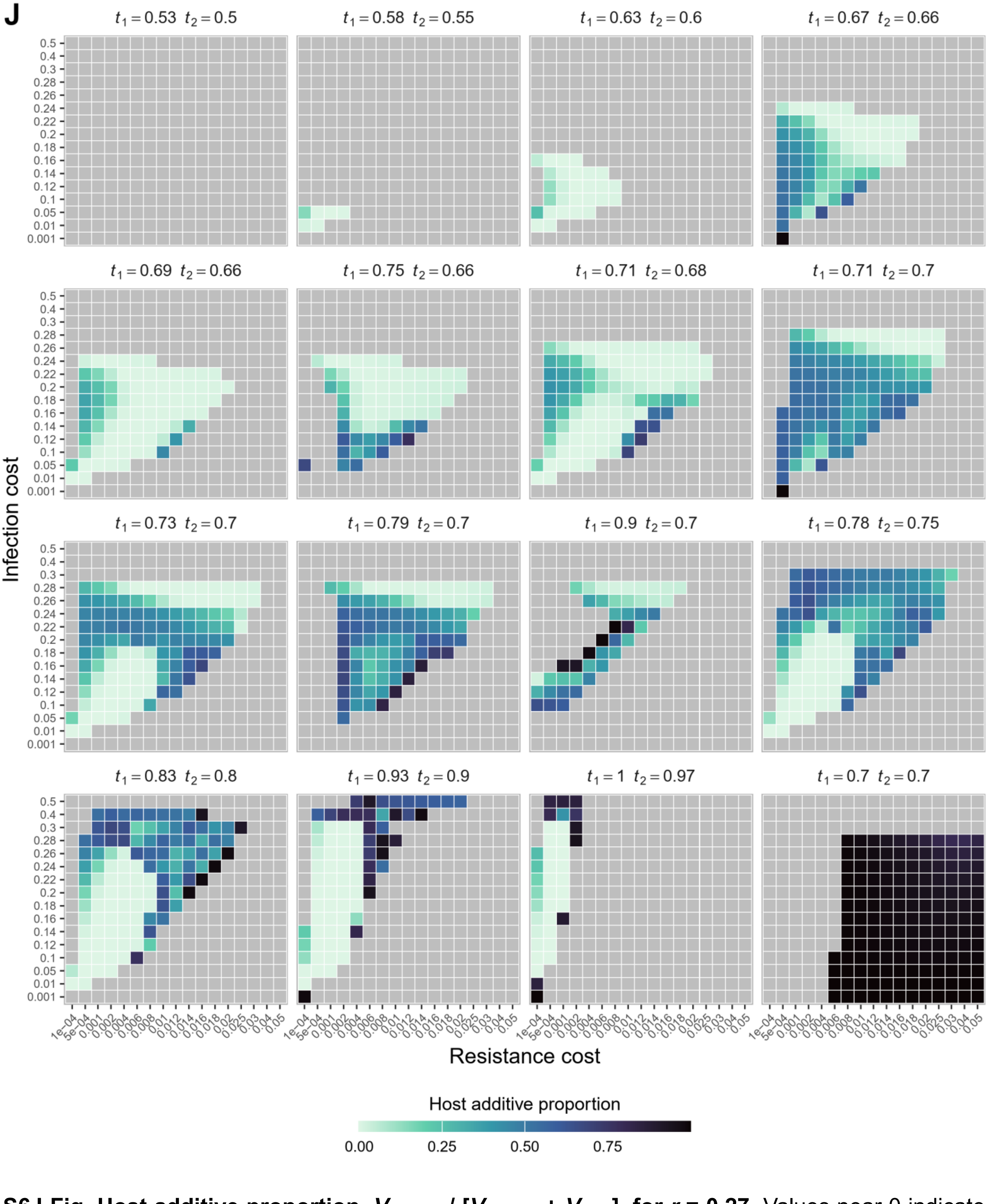

S6J Fig. Host additive proportion, $V_{G(host)}$ / [$V_{G(host)}$ + $V_{G\times G}$], for *r* = 0.27. Values near 0 indicate that these genetic components are dominated by genotype interactions; values near 1 indicate that the host main effect accounts for most of their combined variance. The data supporting this figure can be found in S2 Data.

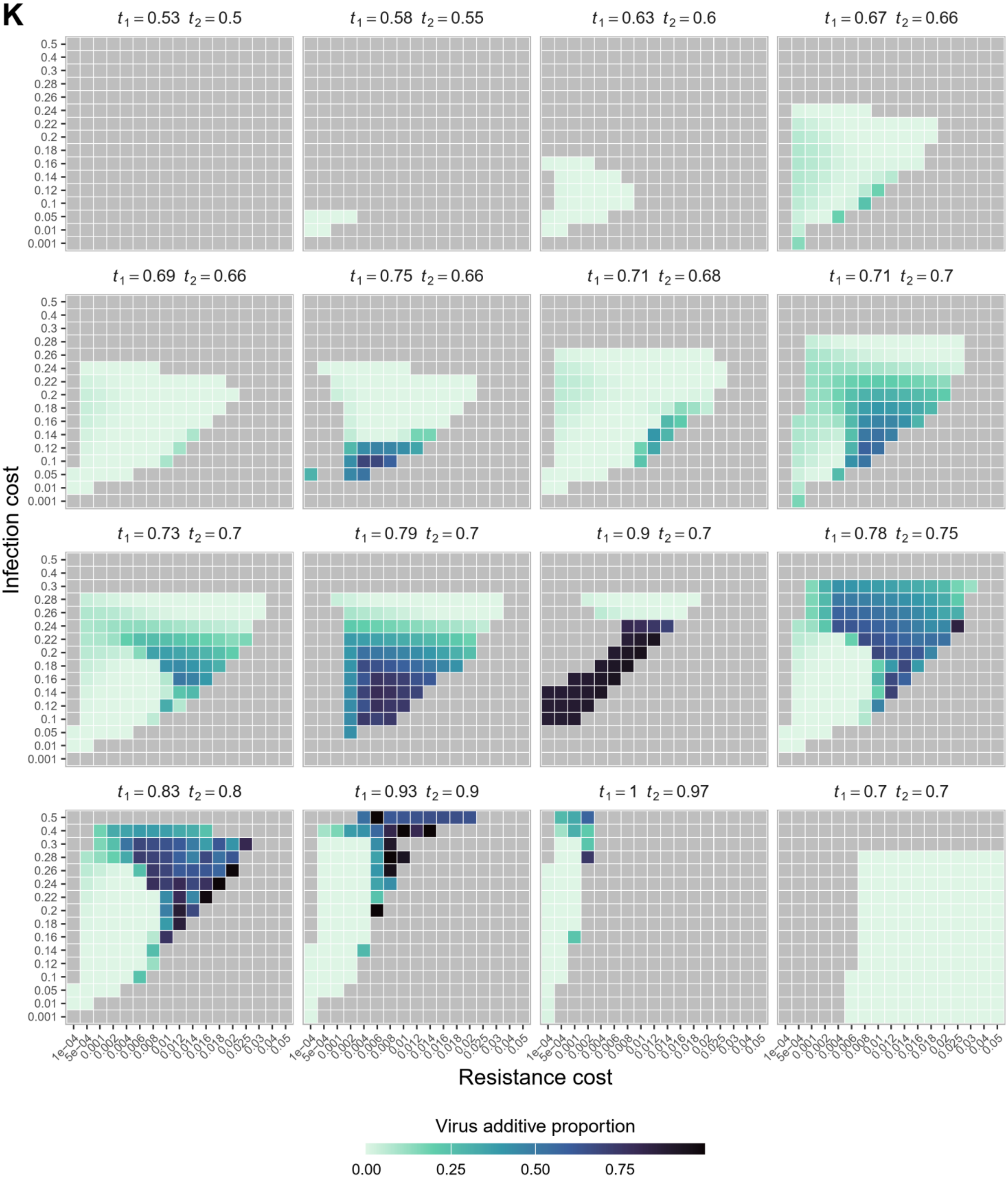

S6K Fig. Virus additive proportion, $V_{G(virus)}$ / [$V_{G(virus)}$ + $V_{G×G}$], for r = 0.27. Values near 0 indicate that these genetic components are dominated by genotype interactions; values near 1 indicate that the virus main effect accounts for most of their combined variance. The data supporting this figure can be found in S2 Data.
