# Supplementary material for: Host–virus interactions dominate fitness and conceal additive genetic variation
Source: PLoS Biol. 2026 Oct 22;24(10):e3003836. doi: 10.1371/journal.pbio.3003836 (PMC13690250; doi:10.1371/journal.pbio.3003836)
Supplement: Supplementary file 3 [file pbio.3003836.s001.docx]

S1 Text

This section extends the simple model of equations (1)–(3) in the main text to two co-circulating viral strains and two biallelic host resistance loci. This gives the generation-by-generation update rule used in all simulations. The notation introduced in the main text is reused throughout.

# S1.1 Full coevolution model

The population at the start of generation *g* is described by a 3 × 9 frequency matrix *x_i,H_*(*g*), where the row index *i* indexes infection state and the column index *H* indexes diploid host genotype at the two biallelic resistance loci *A/a* and *B/b*. Rows are ordered as row 1 = infected with Virus 1, row 2 = infected with Virus 2, row 3 = uninfected. The nine diploid genotypes are AABB, AABb, AAbb, AaBB, AaBb, Aabb, aaBB, aaBb, aabb. Frequencies sum to one,

$$\sum_{i,H} x_{i,H}^{\left( g \right)} = 1$$

Quantities in the derivation below refer to the same generation *g* unless primed (which denotes the next generation, *g* + 1).

## **Genotype-specific notation**

Two indicators describe how the resistance parameters *r* and *d* are applied to each host genotype:

*D(H)* — the number of loci at which host genotype *H* carries a dominant resistance allele (*A* at locus *A*, or *B* at locus *B*). *D*(*H*) therefore takes values 0, 1, or 2 and determines the per-host fertility penalty (1 − *d*)*^D(H)^*. The values of *D*(*H*) for each of the nine genotypes are given by the dominance definition and are listed explicitly in Table A.

1*_i,H_* — a binary resistance indicator equal to 1 if host *H* is resistant to virus *i* and 0 otherwise. Values depend on the coevolution regime (GFG1, GFG2, MA, or selective sweep) and are specified in Table A.

All other parameters (*t*_i_, *c*, *r*, *d*) follow the definitions given in the Materials and Methods.

Two further conventions are used throughout the derivation. A prime denotes a next-generation quantity: *x*′_i,H_ ≡ *x*_i,H_(*g* + 1), matching the main-text convention in which *p*′ is the next-generation adult infected frequency. *W*_d_ and *W*_c_ are the normalisation constants introduced in Steps 1 and 5 below (equations 4 and 12); they rescale the state so frequencies continue to sum to one after fertility selection and after viability selection respectively.

## **Generation cycle**

Each generation proceeds through five sequential steps, which correspond directly to the biological processes shown in Fig 9: (1) resistance cost on fertility, (2) viral transmission to gametes, (3) haplotype gamete pool, (4) random fertilisation and infection of zygotes, (5) viability selection on infected zygotes.

*Step 1 — Fertility selection.* Each host contributes to the gamete pool in proportion to its fertility. Hosts carrying dominant resistance alleles pay a multiplicative fertility cost (1 − *d*)^D(H)^. The post-selection parental frequencies are

$$y_{i,H} =\frac{x_{i,H} \cdot\left( 1 - d \right)^{D\left( H \right)}}{W_{d}}, W_{d} = \sum_{i,H} x_{i,H} \left( 1 - d \right)^{D\left( H \right)} \left( 4 \right)$$

*Step 2 — Effective transmission rate.* The transmission rate of virus *i* in host *H* is reduced by the factor (1 − *r*) whenever the host carries resistance matched to that virus,

$$t_{i,H}= t_{i}\left( 1 -r{\cdot1}_{i,H} \right) \left( 5 \right)$$

The resistance indicator 1*_i,H_* is defined model-by-model in Table A.

*Step 3 — Gamete pool.* Each parent contributes gametes that carry one of four haplotypes *h* ∈ {AB, Ab, aB, ab}. Let *m*_H,h_ denote the Mendelian segregation weight — the probability that a gamete from diploid genotype *H* carries haplotype *h* under free recombination; these weights take the values 0, ½, or ¼ according to the standard two-locus tables.

An infected parent (rows *i* = 1, 2) transmits virus *i* to a fraction *t*_i,H_ of its gametes and leaves the remainder uninfected. Uninfected parents (row 3) contribute only uninfected gametes. Pooling across genotypes gives the infected-gamete frequency, separately for each virus and haplotype,

$$g_{i,h}^{I}= \sum_{H} y_{i,H}\cdot t_{i,H}\cdot m_{H,h} \left( 6 \right)$$

and the uninfected-gamete frequency, by haplotype,

$$g_{h}^{U}= \sum_{H} \left[ y_{3,H}+ \sum_{i\in\{1,2\}} y_{i,H}\cdot(1 - t_{i,H}) \right]\cdot m_{H,h} \left( 7 \right)$$

The total haplotype frequency, summed over infection states, is

$$g_{h}= g_{h}^{U}+ \sum_{i\in\left\{ 1,2 \right\}} g_{i,h}^{I} \left( 8 \right)$$

*Step 4 — Zygote formation.* Male and female gametes unite at random. A zygote with diploid genotype *H* is formed by combining a maternal haplotype *h*_1_ with a paternal haplotype *h*_2_; write the Mendelian weight of that combination as *u*_h1,h2→H_ (the probability, under free recombination, that the pair *h*_1_, *h*_2_ produces diploid *H*). A zygote is infected either (a) through the maternal gamete, or (b) through the paternal gamete when the maternal gamete is uninfected. Summing over haplotype pairs, the infected-zygote frequency for virus *i* and genotype *H* is

$$z_{i,H}= \sum_{h_{1},h_{2}} \left. u_{h_{1},h_{2}\to H} \right.\cdot\left[ g_{i,h_{1}}^{I}\cdot g_{h_{2}}+ g_{h_{1}}^{U}\cdot g_{i,h_{2}}^{I} \right] \left( 9 \right)$$

and the uninfected-zygote frequency for genotype *H* is

$$z_{3,H}= \sum_{h_{1},h_{2}} u_{h_{1},h_{2}\to H}\cdot\left[ g_{h_{1}}^{U}\cdot g_{h_{2}}^{U} \right] \left( 10 \right)$$

Equations (9)–(10) generalize the two transmission routes of equation (2) in the main text: the first term inside the brackets of (9) is the maternal-transmission route (the simple-model term $\bar{p}$), and the second is the paternal route ((1 − $\bar{p}$) $\bar{p}$).

*Step 5 — Viability selection and renormalisation.* Infected zygotes incur a viability cost *c* before reaching the adult census; uninfected zygotes do not. The cost is the same for both viruses. Applying this cost and renormalizing so that the frequency matrix again sums to one gives the next-generation adult state (primed),

$$x_{i,H}^{'}= \frac{z_{i,H} \left( 1 - c \cdot1_{i\in\left\{ 1,2 \right\}} \right)}{W_{c}} \left( 11 \right)$$

$$W_{c}= \sum_{H} z_{3,H} + \left( 1 - c \right) \cdot\sum_{i\in\left\{ 1,2 \right\},H} z_{i,H} \left( 12 \right)$$

## Equations (4)–(12) close the recursion, mapping *x* to *x*′.

## **Sensitivity to the timing of virus loss**

Vertical-transmission rates are measured from infected adults to their adult offspring and therefore do not identify the stage at which virus loss occurs. In the original model, all incomplete transmission occurs during the production of gametes, and infected zygotes that survive the infection-induced viability cost remain infected until the adult census. We examined whether this assumption affected the coevolutionary dynamics by moving the existing virus-loss event to different stages of the life cycle, without adding an additional source of loss.

For virus *i* and host genotype *G*, the effective probability of retaining the virus was $t_{i,G}=t_{i}\left( 1-r\cdot1_{i,G} \right)$, as in the original model, where $1_{i,G}=1$ if host genotype *G* is resistant to virus *i*, and 0 otherwise. The complementary fraction $1-t_{i,G}$, lost the virus and entered the uninfected class while retaining the same host genotype.

We compared three positions of virus loss. In the original model, loss occurs during transmission to gametes and resistance acts according to the genotype of the infected parent. In the first alternative, loss occurs immediately after fertilisation but before viability selection, with resistance acting according to the offspring genotype. Zygotes that lose the virus therefore avoid the infection-induced viability cost *c*. In the second alternative, infected zygotes first experience the viability cost; among those that survive, virus loss then occurs before the adult census, again with resistance acting according to the offspring genotype.

The GFG1 parameter sweep was repeated for all three models using the parameter ranges and initial conditions of Fig 3, with each parameter combination simulated for 10,000 generations. The models were compared using the coefficient of variation of viral-genotype frequencies and the resulting classification of coevolutionary dynamics. Moving virus loss to after viability selection produced the same outcome classification as the original model in 286 of 288 parameter combinations. Loss before viability selection resulted in stronger damping and shifted the boundaries among dynamical regimes, but persistent oscillations remained possible under all three timings (S8 Fig).

## **Interaction matrices**

The three coevolution regimes (GFG1, GFG2, MA) differ only in the definitions of the resistance indicator 1_i,H_ and, consequently, in which genotypes contribute to *D*(*H*). All other steps of the generation cycle (equations 4–12) are unchanged across regimes. The selective sweep model uses the GFG2 interaction matrix with *d* = 0 (cost-free resistance) and sequential introduction of Virus 2 and allele *B* at fixed generations (see S4 Fig).

**Table A.** Resistance indicators 1_1,H_, 1_2,H_ and dominant-allele locus count *D*(*H*) for each of the nine diploid host genotypes, under the three genetic models (GFG1, GFG2, MA). The selective sweep model uses the GFG2 column entries throughout and sets *d* = 0.

| **Host genotype H** | ***D(H)*** | **GFG1: (1_₁,_ *_H_*, 1_₂,_ *_H_*)** | **GFG2: (1_₁,_ *_H_*, 1_₂,_ *_H_*)** | **MA: (1_₁,_ *_H_*, 1_₂,_ *_H_*)** |
| --- | --- | --- | --- | --- |
| *AABB* | 2 | (1, 0) | (1, 1) | (1, 0) |
| *AABb* | 2 | (1, 0) | (1, 1) | (1, 0) |
| *AAbb* | 1 | (1, 0) | (1, 0) | (1, 0) |
| *AaBB* | 2 | (1, 0) | (1, 1) | (1, 0) |
| *AaBb* | 2 | (1, 0) | (1, 1) | (1, 0) |
| *Aabb* | 1 | (1, 0) | (1, 0) | (1, 0) |
| *aaBB* | 1 | (0, 0) | (0, 1) | (0, 1) |
| *aaBb* | 1 | (0, 0) | (0, 1) | (0, 1) |
| *aabb* | 0 | (0, 0) | (0, 0) | (0, 1) |

In GFG1, allele *B* is absent from the population throughout the simulation, so the trajectory remains confined to the three *B*-free genotypes *AAbb*, *Aabb* and *aabb*; the full nine-genotype listing in Table A is included only for reference, and the additional genotypes appear with frequency zero. In MA, the aa genotype is resistant to Virus 2 yet carries no dominant resistance allele at locus *A*, so it contributes 0 to *D*(*H*) — this asymmetry is what breaks the symmetry between host and virus trade-offs in MA.

## **Fitness-variance decomposition**

At the start of each generation, and before advancing the recursion, we compute the realized fitness of every host-virus combination and decompose its variance across the joint distribution *x*_i,H_ into four components: two additive terms (host-genotype and virus-genotype main effects), one non-additive host × virus interaction term, and a covariance term that captures linkage between host genotype and infection status.

The fitness expressions depend on the population-wide infected-gamete frequency, the full-model analogue of the main-text $\bar{p}$. After Step 1 the fraction of gametes in the pool that carry a virus is

$$\bar{p}= \sum_{i,H} y_{i,H} \cdot t_{i,H}= \frac{\sum_{i,H} x_{i,H} {(1 - d)}^{D(H)} t_{i,H}}{W_{d}} \left( 13 \right)$$

with *t_3,H_* = 0 for the uninfected row so that uninfected parents contribute only to the denominator. In the simple model of equations (1)–(3) this reduces to $\bar{p}$ = *p* · *t*(1 − *r*).

Host fitness is the expected number of surviving offspring produced by a parent of type (*i*, *H*), summing maternally- and paternally transmitted infection routes and weighting by offspring viability,

$$w_{H}\left( i,H \right)= \left( 1 - d \right)^{D\left( H \right)}\cdot\left[ t_{i,H} \left( 1 - c \right)+ \left( 1 - t_{i,H} \right)\left( 1 - c\bar{p} \right) \right] \left( 14 \right)$$

The first term inside the brackets is the maternal-transmission route — gametes carrying virus *i* that survive at rate 1 − *c*. The second term is the paternal route applied to the parent's uninfected gametes: each such gamete pairs with an infected mate with probability $\bar{p}$ (surviving at rate 1 − *c*) or with an uninfected mate with probability 1 − $\bar{p}$ (surviving at rate 1), for an average viability of 1 − *c*$\bar{p}$. For an uninfected parent (*i* = 3), set *t_3,H_* = 0 and the expression reduces to (1 − *d*)^D(H)^ (1 − *c*$\bar{p}$) — the paternal route alone.

Virus fitness is the expected number of infected offspring transmitted by a virus-carrying parent; it is defined only for *i* ∈ {1, 2}:

$$w_{V}\left( i, H \right)= {(1 - d)}^{D(H)} \cdot t_{i,H} \cdot\left( 1 -\frac{\bar{p}}{2} \right) \left( 15 \right)$$

The factor (1 − $\bar{p}$ / 2) corrects for coinfection across the two transmission routes: an infected gamete whose partner gamete is already infected does not contribute a new viral lineage, and averaging this discount over the maternal and paternal routes yields the factor above.

Both fitness functions are treated as functions of the joint state (*i*, *H*), meaning that an individual's fitness depends on both its host genotype *H* and its infection state *i*.

For the host-fitness decomposition, the frequencies refer to the full population, including the uninfected state *i*=3. For the virus-fitness decomposition, which is defined only for *i* ∈ {1, 2}, the frequencies are conditioned on infection and renormalised to sum to one across the infected states.

The row marginal *P(i) = ∑_H_ x_i,H_* is the overall frequency of individuals in infection state *i*, summed across all host genotypes (i.e. the proportion of the whole population currently in state *i*). The column marginal *P(H) = ∑_i_ x_ᵢ,H_* is the overall frequency of host genotype *H* in the population, summed across all infection states (i.e. the genotype frequency of *H* regardless of whether those individuals are infected or uninfected). The conditional probabilities *P(i | H) = x_ᵢ,H_ / P(H)* and *P(H | i) = x_ᵢ,H_ / P(i)* describe, respectively, how likely each infection state is among hosts of a given genotype, and how likely each host genotype is among individuals in a given infection state.

For any host-virus combination (*i,H*), let *w*(*i,H*) denote the fitness function being decomposed — either host fitness *w_H_(i,H)* from equation (14) or virus fitness *w_V_(i,H)* from equation (15). The decomposition is applied separately to each. Two conditional means and one grand mean:

$$\mu_{H}= \sum_{i} P(i | H) w(i,H), \mu_{i}= \sum_{H} P(H | i) w(i,H) \left( 16 \right)$$

$$\mu= \sum_{i,H} x_{i,H} w\left( i, H \right)\left( 17 \right)$$

*μ_H_* is the mean fitness of genotype *H*, averaged over the infection states currently carried by that genotype, weighted by *P(i | H) = x_i,H_ / P(H).* *μ_i_* is the mean fitness within infection class *i*, averaged over the genotypes currently in that class, weighted by *P(H | i) = x_i,H_ / P(i)*. *μ* is the grand mean across the whole population.

The variance is then partitioned into four components:

$$V_{G(host)} = \sum_{H} P\left( H \right)\left( \mu_{H} - \mu\right)^{2}, V_{G(virus)} = \sum_{i} P\left( i \right)\left( \mu_{i} - \mu\right)^{2} \left( 18 \right)$$

*V*_G(_*_host_*_)_ is the host main effect: each host genotype is summarized by its conditional mean fitness μ_H_, and *V*_G(host)_ is the variance of these per-genotype means weighted by the genotype frequencies *P*(*H*). It measures how much of the variance in *w* is captured by host genotype alone, treating each genotype as a single number. *V*_G(virus)_ is the virus main effect, computed in the same way for infection state: each state is summarized by *μᵢ*, and *V_G(virus_*_)_ is the variance of these per-state means weighted by *P*(*i*). Together *V_G(host)_* and *V_G(virus)_* are the main effect components of the decomposition: their sum is the variance that would be present in *w* if host and virus contributed independently.

$$V_{G\times G} = \sum_{i,H} x_{i,H}\left[ w(i, H) - \mu_{H} - \mu_{i} + \mu\right]^{2} \left( 19 \right)$$

*V_G×G_* is the non-additive host-by-virus interaction term. For each cell (*i*,*H*), the additive prediction μ_H_ + μᵢ − μ is the fitness expected in that cell if host genotype and infection state contributed independently to *w*. The deviation *w*(*i*,*H*) − (μ_H_ + μᵢ − μ) is the part of the cell's fitness not explained by the two main effects, and *V_G×G_* is the population-weighted mean of these squared deviations.

$$V_{cov} = V_{total}- V_{G\left( host \right)} - V_{G\left( virus \right)} - V_{G\times G}, V_{total} = \sum_{i,H} x_{i,H}\left[ w(i, H) - \mu\right]^{2} \left( 20 \right)$$

*V*_cov_ is the residual: it is whatever remains after *V*_G(host)_, *V*_G(virus)_ and *V_G×G_* have been subtracted from the total variance *V_total_ = ∑_i,H_ x_i,H_ (w(i,H) − μ)².* Mechanically, *V*_cov_ captures linkage (non-independence) between host genotype and infection status. It becomes non-zero whenever specific host genotypes are disproportionately likely to carry specific viruses, i.e. when coevolutionary dynamics build linkage disequilibrium between host genotype and infection status.

The decomposition is applied separately to *w_H_* and *w_V_*. These steps result in the eight quantities — *V_G(host)_, V_G(virus)_, V_G×G_, V_cov_* for each of host and virus fitness (see S2 Data).

Simulations were implemented in R 4.4.0 using only base linear-algebra primitives.

## **Selective sweep model**

The selective sweep model uses the GFG2 interaction matrix (Table A) and sets *d* = 0 throughout (cost-free resistance). Simulations were run for 1,250 generations. Virus 1 and the resistant allele *A* were present from generation 0 at low frequency (infected total for virus 1 = 0.0001, prevalence of the allele *A* = 0.0001), while Virus 2 and allele *B* were initially absent. At generation 600, Virus 2 was introduced at 0.01% total frequency (infected total for virus 2 = 0.0001). At generation 700, allele *B* was introduced at frequency 0.0001 (prevalence of the allele *B* = 0.0001). Parameters were: *t₁* = 0.73, *t₂* = 0.70, *c* = 0.23, *r* = 0.27, *d* = 0. This design follows the hard-sweep scenario, where cost-free resistance allows the *B*-carrying haplotype to sweep to fixation following its introduction.

## **Parameter sweep**

Equations (4)–(12) were iterated deterministically for 40,000 generations for each parameter combination under the GFG1 interaction model. Simulations started with Virus 1 at a frequency of 0.2, Virus 2 at 0.1, resistance allele *A* at 0.1, and allele *B* absent. Mean frequencies and variance components were calculated over the final 50% of each simulation. Unfiltered coefficients of variation used to aid dynamical classification were calculated over the final 30%, whereas prevalence-filtered summaries used only generations within the final 50% in which both viral genotypes had frequencies above 0.0001.

Two parameters were varied on a grid: resistance cost *d* (horizontal axis) across 18 values spanning 0–0.05, and infection cost *c* (vertical axis) across 16 values spanning 0.001–0.5. Sixteen (*t₁, t₂*) transmission-rate pairs were used: the base transmission rate *t₁* spanned 0.53–1, and the cost of viral escape from host resistance, which is the reduction in base transmission rate carried by the escaping virus (*t₁ − t₂*), was tested at five levels: 0%, 1%, 3%, 9%, and 20%. The sweep was performed at two levels of resistance effect: *r* = 0.27 (S6 Fig) and *r* = 0.70 (S7 Fig).

For each parameter combination the simulation recorded: mean frequencies of resistance alleles and virus genotypes; the fitness variance components V_G(host)_, V_G(virus)_, and V_G×G_ for both host and virus fitness; the coefficient of variation (CV) of Virus 1, Virus 2, and allele *A* frequencies; the host and virus additive proportions V_G(host)_/[V_G(host)_ + V_G×G_] and V_G(virus)_/[V_G(virus)_ + V_G×G_]; and the qualitative type of coevolutionary dynamics classified from the tail window (S6 and S7 Figs).

Each simulation was assigned to one of seven outcomes. A run was stopped early if (i) both viral prevalences dropped below 10⁻¹⁵ (parasite extinction); (ii) one virus dropped below 10⁻³⁰ (competitive exclusion); or (iii) the resistance allele dropped below 10⁻³⁰ (susceptibility fixation). When one virus was lost, the run was labelled unstable equilibrium if the lost virus had at any point in the tail-window exceeded the surviving virus, and competitive exclusion otherwise. For runs completing all 40,000 generations, mean prevalence, CV, and a damping ratio comparing peak amplitudes at the start and end of the tail were used to assign each run to one of four outcomes. Runs with CV = 0 were assigned to stable equilibrium. For oscillatory runs (mean prevalence > 0.001 and CV > 0), a positive value of the damping ratio indicated damped oscillations (consistently shrinking peaks), and persistent oscillations otherwise. The persistent vs unstable boundary is the most sensitive, so ambiguous runs were given the most conservative label, persistent oscillations. Boundaries between slow regimes should therefore be treated as approximate.

*Parameter space heatmaps.* S6 and S7 Figs show the full heatmaps for *r* = 0.27 and *r* = 0.70 respectively. Each panel covers the *c* × *d* grid with one panel per transmission-rate pair. For all plotted statistics, only tail-window generations in which the prevalence of both viruses exceeded 0.01% were retained. Grey cells indicate parameter combinations for which no generations passed this prevalence filter.

The qualitative outcome across parameter space is shown in S6A and S7A Figs. At resistance effect *r* = 0.27, oscillatory runs arose mostly at infection costs around 0.01–0.4 and resistance costs 0.0001–0.03. Across parameter combinations retaining an infectivity cost, the fraction of oscillatory runs generally increased as the base transmission rate pair rose from *t*_1_ = 0.53, *t*_2_ = 0.50 to *t*_1_ = 0.83, *t*_2_ = 0.80, with no oscillations at the lowest pair. At *r* = 0.70, the pattern was similar but oscillations extended across slightly wider ranges of infection and resistance costs, from 0.001 to 0.4 and from 0.0001 to 0.05. Oscillation amplitudes are reflected in the CVs of Virus 1, Virus 2 and allele *A* frequencies (S6B–S6D and S7B–S7D Figs). Outside the maintained-polymorphism window, runs ended in competitive exclusion (the most common outcome), susceptibility fixation, or parasite extinction. Mean frequencies of Virus 1, Virus 2 and allele *A* are shown in S6E–S6G and S7E–S7G Figs.

Across the maintained-polymorphism window, V_G×G_ usually dominated fitness variance for both host and virus (S6H and S6I, S7H and S7I Figs), with the additive proportions V_G(host)_/[V_G(host)_ + V_G×G_] and V_G(virus)_/[V_G(virus)_ + V_G×G_] remaining small throughout (S6J, S6K, S7J and S7K Figs). This conclusion holds regardless of slow-regime assignment, as many runs sustained oscillations for tens of thousands of generations with trends too small to detect over the simulation window, making boundaries between slow regimes a coarse map rather than a precise classification.

# S1.2 Tables

**Table B. Genetic variance components (intra-class correlations) from univariate models**

Univariate linear mixed models were fitted separately for each trait, with host, virus and host × virus interaction included as random effects. Variance components were scaled to intraclass correlations (ICC), representing the proportion of total phenotypic variance explained by each component. Genetic variance (%) was calculated from the unrounded estimates by dividing each component by the sum of the host, virus and host × virus interaction components within that trait. Significance was assessed using likelihood-ratio tests comparing full and reduced models, assuming a 50:50 mix of χ² distributions with zero and one degree of freedom.

| Trait | *Component* | *Estimate (ICC)* | *SE* | Genetic variance (%) | *Bound* | *Χ^2^* | *p-value* |
| --- | --- | --- | --- | --- | --- | --- | --- |
| Viral load (M) | Host | 0.094 | 0.062 | 36.9 | P | 7.63 | 0.003 |
| Viral load (M) | Virus | 0.161 | 0.084 | 63.1 | P | 13.60 | <0.001 |
| Viral load (M) | Host:Virus | 0.000 | NA | 0.0 | B | 0.00 | 0.500 |
| Viral load (F) | Host | 0.053 | 0.051 | 8.9 | P | 2.25 | 0.067 |
| Viral load (F) | Virus | 0.259 | 0.114 | 43.7 | P | 17.47 | <0.001 |
| Viral load (F) | Host:Virus | 0.281 | 0.090 | 47.4 | P | 13.26 | <0.001 |
| Transmission (M) | Host | 0.083 | 0.057 | 14.7 | P | 6.35 | 0.006 |
| Transmission (M) | Virus | 0.131 | 0.073 | 23.3 | P | 11.01 | <0.001 |
| Transmission (M) | Host:Virus | 0.351 | 0.059 | 62.1 | P | 468.99 | <0.001 |
| Transmission (F) | Host | 0.165 | 0.086 | 30.2 | P | 15.64 | <0.001 |
| Transmission (F) | Virus | 0.039 | 0.038 | 7.1 | P | 2.06 | 0.076 |
| Transmission (F) | Host:Virus | 0.342 | 0.059 | 62.6 | P | 500.85 | <0.001 |
| Fecundity | Host | 0.096 | 0.056 | 40.2 | P | 16.10 | <0.001 |
| Fecundity | Virus | 0.016 | 0.018 | 6.6 | P | 1.39 | 0.119 |
| Fecundity | Host:Virus | 0.127 | 0.031 | 53.2 | P | 53.98 | <0.001 |
| Hatch rate | Host | 0.011 | 0.015 | 11.9 | P | 1.03 | 0.155 |
| Hatch rate | Virus | 0.000 | NA | 0.0 | B | 0.00 | 0.500 |
| Hatch rate | Host:Virus | 0.082 | 0.024 | 88.1 | P | 25.42 | <0.001 |

*Notes: P indicates a positive estimate in the unconstrained parameter space, and B indicates an estimate constrained to the boundary at zero. SE was not estimable for boundary cases. F and M denote female and male flies, respectively. The data underlying this table can be found in S1 Data.*

**Table C. Genetic correlations from the multivariate (five-trait) model**

A multivariate linear mixed model was fitted to estimate genetic correlations among viral load in females and males, transmission in females and males, and fecundity at the host, virus and host × virus interaction levels. Traits were scaled to standard deviation units. Approximate 95% confidence intervals can be calculated as the estimate ± 1.96 × SE.

| Level | *Trait 1* | *Trait 2* | *Correlation (r)* | *SE* | *Bound* |
| --- | --- | --- | --- | --- | --- |
| Host | Viral load (F) | Viral load (M) | 0.710 | 0.382 | U |
| Host | Transmission (M) | Viral load (M) | 0.528 | 0.416 | U |
| Host | Transmission (M) | Viral load (F) | 0.502 | 0.499 | U |
| Host | Transmission (F) | Viral load (M) | 0.893 | 0.210 | U |
| Host | Transmission (F) | Viral load (F) | 1.000 | NA | B |
| Host | Transmission (F) | Transmission (M) | 0.197 | 0.451 | U |
| Host | Fecundity | Viral load (M) | −0.355 | 0.414 | U |
| Host | Fecundity | Viral load (F) | −0.143 | 0.509 | U |
| Host | Fecundity | Transmission (M) | −1.000 | NA | B |
| Host | Fecundity | Transmission (F) | −0.112 | 0.412 | U |
| Virus | Viral load (F) | Viral load (M) | 1.000 | NA | B |
| Virus | Transmission (M) | Viral load (M) | 0.320 | 0.387 | U |
| Virus | Transmission (M) | Viral load (F) | −0.202 | 0.399 | U |
| Virus | Transmission (F) | Viral load (M) | 1.000 | NA | B |
| Virus | Transmission (F) | Viral load (F) | 0.458 | 0.335 | U |
| Virus | Transmission (F) | Transmission (M) | 0.571 | 0.387 | U |
| Virus | Fecundity | Viral load (M) | −0.006 | 0.571 | U |
| Virus | Fecundity | Viral load (F) | 0.004 | 0.557 | U |
| Virus | Fecundity | Transmission (M) | −0.747 | 0.451 | U |
| Virus | Fecundity | Transmission (F) | −0.355 | 0.682 | U |
| Host:Virus | Viral load (F) | Viral load (M) | NA | NA | B |
| Host:Virus | Transmission (M) | Viral load (M) | NA | NA | B |
| Host:Virus | Transmission (M) | Viral load (F) | −0.002 | 0.166 | U |
| Host:Virus | Transmission (F) | Viral load (M) | NA | NA | B |
| Host:Virus | Transmission (F) | Viral load (F) | 0.419 | 0.148 | U |
| Host:Virus | Transmission (F) | Transmission (M) | 0.332 | 0.116 | U |
| Host:Virus | Fecundity | Viral load (M) | NA | NA | B |
| Host:Virus | Fecundity | Viral load (F) | 0.058 | 0.195 | U |
| Host:Virus | Fecundity | Transmission (M) | −0.007 | 0.150 | U |
| Host:Virus | Fecundity | Transmission (F) | −0.013 | 0.151 | U |

*Notes: U indicates an unconstrained estimate, and B indicates a boundary estimate. For boundary entries, the correlation was either constrained to ±1 or could not be estimated because the variance of one trait was constrained to zero. NA indicates an undefined correlation due to zero variance in at least one trait. F and M denote female and male flies, respectively. The data underlying this table can be found in S1 Data.*

**Table D. Genetic correlations from bivariate models**

Separate bivariate models were fitted to estimate cross-sex genetic correlations for viral load and transmission and correlations between hatch rate and the other five traits. Bivariate models were used because hatch rate could not be included in the full multivariate model and to obtain focused estimates of the cross-sex correlations.

**a. Cross‑sex correlations for viral load and transmission**

| Level | *Trait 1* | *Trait 2* | *Correlation (r)* | *SE* | *Bound* |
| --- | --- | --- | --- | --- | --- |
| Host | Viral load (F) | Viral load (M) | 0.757 | 0.428 | U |
| Virus | Viral load (F) | Viral load (M) | 0.999 | NA | B |
| Host:Virus | Viral load (F) | Viral load (M) | NA | NA | B |
| Host | Transmission (F) | Transmission (M) | 0.168 | 0.461 | U |
| Virus | Transmission (F) | Transmission (M) | 0.535 | 0.436 | U |
| Host:Virus | Transmission (F) | Transmission (M) | 0.339 | 0.117 | U |

**b. Correlations involving hatch rate**

| Level | Trait 1 | Trait 2 | Correlation (r) | SE | Bound |
| --- | --- | --- | --- | --- | --- |
| Host | Hatch rate | Viral load (M) | −1.000 | NA | B |
| Host | Hatch rate | Viral load (F) | −1.000 | NA | B |
| Host | Hatch rate | Transmission (M) | −1.000 | NA | B |
| Host | Hatch rate | Transmission (F) | −0.427 | 0.593 | U |
| Host | Hatch rate | Fecundity | −0.739 | 0.644 | U |
| Virus | Hatch rate | All traits | NA | NA | B |
| Host:Virus | Hatch rate | Viral load (M) | NA | NA | B |
| Host:Virus | Hatch rate | Viral load (F) | −0.269 | 0.213 | U |
| Host:Virus | Hatch rate | Transmission (M) | −0.044 | 0.172 | U |
| Host:Virus | Hatch rate | Transmission (F) | −0.280 | 0.165 | U |
| Host:Virus | Hatch rate | Fecundity | 0.508 | 0.177 | U |

*Notes: U indicates an unconstrained estimate, and B indicates a boundary estimate. NA indicates an undefined correlation due to zero variance in at least one trait. All virus-level correlations involving hatch rate were undefined because the virus variance in hatch rate was constrained to zero. The host × virus correlation between hatch rate and male viral load was undefined because the male viral-load host × virus variance was constrained to zero. F and M denote female and male flies, respectively. The data underlying this table can be found in S1 Data.*

**Table E. Primers used in this study**

| Primer name | *Primer sequence (5'–3')* | *Application* |
| --- | --- | --- |
| MiS_9_F | ACTTCGATGGAGAGGGCAAT | Sanger sequencing |
| MiS_9_R | TGTCAGATGCATGAGGAACC | Sanger sequencing |
| MiS_81_F | AGACGACGGTGGAGAAAGAG | Sanger sequencing |
| MiS_81_R | CCAATCGTGGCCATGACATT | Sanger sequencing |
| MiS_72_F | GGAGGCGTTCATTCGTTGGTT | Sanger sequencing |
| MiS_72_R | CCATCCCCTGACTTCCGATCT | Sanger sequencing |
| MiS_62_F | CTTCCGACCTCTGCCCTTG | Sanger sequencing |
| MiS_62_R | AGGGTGACTCCTTTGTGATTGA | Sanger sequencing |
| MiS_63_F | CTTCCGACCTCTGCCCTTGA | Sanger sequencing |
| MiS_63_R | GGGTGACTCCTTTGTGATTGATCC | Sanger sequencing |
| MiS_52_F | CCTAGTTGAGAGACCAAGCCTAA | Sanger sequencing |
| MiS_52_R | CCCACATTAATTCTGTTGCAGCCT | Sanger sequencing |
| MiS_4_F | CCATCCCGGAACTAATGAACCA | Sanger sequencing |
| MiS_4_R | TCTCCATACAGCCGAGATGA | Sanger sequencing |
| MiS_31_F | TGGAGCACCCCTCTTAAACC | Sanger sequencing |
| MiS_31_R | GAGGTCCTGATGTTTAGCGTC | Sanger sequencing |
| MiS_22_F | GACGAGATTCACCGGGAAGAA | Sanger sequencing |
| MiS_22_R | ACGGGGTTGTCCGGATACAA | Sanger sequencing |
| MiS_1_F | ATACGCTGTGGTCTGGAGAT | Sanger sequencing |
| MiS_1_R | TGATATTCCGCTAGCCAACCA | Sanger sequencing |
| MiS_13_F | TCCTAGCGATCTTACTGACACA | Sanger sequencing |
| MiS_13_R | AGCGGTTTGATCATCTTGGGT | Sanger sequencing |
| Sig.San.L1_F | TCACAACCGGCTACAGGACC | Sanger sequencing |
| Sig.San.L1_R | ATCCCAATCGGTTAGTAGAAGCA | Sanger sequencing |
| Sig.San.N1_F | CACCCTTGTCCACCTGATCG | Sanger sequencing |
| Sig.San.N1_R | TCATGGTAGGCTGCCTCTTG | Sanger sequencing |
| MiSV.XM2_F | GAAATCAGGGACCAGTGTGC | Sanger sequencing |
| MiSV.XM2_R | CACCTCATGTCCTCCGA | Sanger sequencing |
| SV.PCR.1_F | AATGTCACCACCCGGATCTC | Sanger sequencing |
| SV.PCR.2_F | ATGTCACCACCCGGATCTCA | Sanger sequencing |
| SV.PCR.3_F | GTCACCACCCGGATCTCAA | Sanger sequencing |
| SV.PCR.1_R | YGYMTGGATAGTGYTTCYAGA | Sanger sequencing |
| SV.PCR.2_R | GYMTGGATAGTGYTTCYAGA | Sanger sequencing |
| MS_3.1_F | TCCCACTACTCTGACTCCCC | Sanger sequencing |
| MS_3.1_R | TGTAGCGACAGTTTTATAGCAT | Sanger sequencing |
| MS_3.2_F | CTCCCACTACTCTGACTCCC | Sanger sequencing |
| MS_3.2_R | GTAGCGACAGTTTTATAGCAT | Sanger sequencing |
| SVq.XM_7_F | TCATTACATCAGACTCCACAATG | qRT-PCR (viral load) |
| SVq.XM_7_R | TGGTTCATCTTATTCATGGCG | qRT-PCR (viral load) |

**Table F. Genetic variance components from analyses restricted to five host lines susceptible at all three major-effect resistance loci**

The univariate models described for Table B were repeated for viral load and transmission using DGRP lines 303, 443, 712, 801 and 850, which carry susceptible alleles at *p62, CHKov1* and *Ge-1*. All ten virus isolates were retained. Variance components and their percentages were calculated as described for Table B. Because the restricted analysis contained only five host levels, its estimates of host variance are less precise than those from the complete panel.

| Trait | *Component* | | *Estimate (ICC)* | | *SE* | | *Genetic variance (%)* | | *Bound* | | *Χ^2^* | | *p-value* | |
| --- | --- | --- | --- | --- | --- | --- | --- | --- | --- | --- | --- | --- | --- | --- |
| Viral load (M) | Host | 0.000 | | NA | | 0.0 | | B | | 0.00 | | 0.500 | |  |
| Viral load (M) | Virus | 0.208 | | 0.117 | | 96.7 | | P | | 6.21 | | 0.006 | |  |
| Viral load (M) | Host:Virus | 0.007 | | 0.119 | | 3.3 | | P | | 0.00 | | 0.477 | |  |
| Viral load (F) | Host | 0.009 | | 0.046 | | 1.4 | | P | | 0.04 | | 0.420 | |  |
| Viral load (F) | Virus | 0.269 | | 0.141 | | 44.3 | | P | | 6.78 | | 0.005 | |  |
| Viral load (F) | Host:Virus | 0.329 | | 0.130 | | 54.2 | | P | | 8.38 | | 0.002 | |  |
| Transmission (M) | Host | 0.079 | | 0.074 | | 15.1 | | P | | 3.79 | | 0.026 | |  |
| Transmission (M) | Virus | 0.163 | | 0.093 | | 31.2 | | P | | 7.16 | | 0.004 | |  |
| Transmission (M) | Host:Virus | 0.281 | | 0.071 | | 53.7 | | P | | 157.66 | | <0.001 | |  |
| Transmission (F) | Host | 0.026 | | 0.039 | | 8.4 | | P | | 0.81 | | 0.184 | |  |
| Transmission (F) | Virus | 0.030 | | 0.044 | | 9.7 | | P | | 0.65 | | 0.211 | |  |
| Transmission (F) | Host:Virus | 0.251 | | 0.061 | | 81.9 | | P | | 99.54 | | <0.001 | |  |

*Notes: Bound: P = positive (unconstrained parameter space), B = boundary (variance fixed to zero). F and M denote female and male flies, respectively. SE is not estimable for boundary cases. P-values are from likelihood-ratio tests. The data underlying this table can be found in S1 Data.*

**Table G. Phenotypic correlations among host-virus combination means**

Pearson correlations across the 90 host genotype × virus genotype combinations. Reproductive traits are expressed relative to uninfected control flies of the same host line.

| *Trait 1* | *Trait 2* | *Figure* | *n* | *Correlation (r)* | *95% CI* | *p-value* |
| --- | --- | --- | --- | --- | --- | --- |
| Transmission (F) | Transmission (M) | Fig 4D | 90 | 0.25 | 0.05 to 0.44 | 0.016 |
| Viral load (F) | Viral load (M) | Fig 5D | 89 | 0.50 | 0.33 to 0.64 | <0.001 |
| Viral load (F) | Transmission (F) | Fig 6A | 90 | 0.35 | 0.16 to 0.52 | <0.001 |
| Viral load (M) | Transmission (M) | Fig 6B | 89 | 0.21 | 0.01 to 0.40 | 0.044 |
| Fecundity | Hatch rate | Fig 7C | 90 | 0.17 | −0.04 to 0.36 | 0.118 |
| Hatch rate | Transmission (F) | Fig 8A | 90 | −0.38 | −0.54 to -0.18 | <0.001 |
| Fecundity | Transmission (M) | Fig 8B | 90 | −0.24 | −0.42 to -0.03 | 0.024 |
| Hatch rate | Transmission (M) | S5A Fig | 90 | −0.14 | −0.33 to 0.07 | 0.199 |
| Fecundity | Transmission (F) | S5B Fig | 90 | −0.04 | −0.25 to 0.17 | 0.689 |
| Larval offspring | Transmission (F) | S5C Fig | 90 | −0.19 | −0.38 to 0.02 | 0.070 |
| Larval offspring | Transmission (M) | S5D Fig | 90 | −0.25 | −0.44 to -0.05 | 0.016 |

*Notes: r is the Pearson correlation coefficient with its 95% confidence interval. n is 89 where one host-virus combination lacked a male viral load estimate. The data underlying this table can be found in S1 Data.*

**Table H. Association between viral phylogenetic distance and host-specific effects on transmission and viral load**

For each trait, the mean of each virus across host lines was subtracted from the values for that virus, removing the virus main effect. Euclidean distances between viruses were calculated from these centred host-specific effects and compared with patristic distances from the sigma virus phylogeny using one-sided Mantel tests with 9,999 permutations. Holm-adjusted *p* values account for the four tests.

| *Trait* | *Viruses* | *Host lines* | *Mantel r* | *One-sided p* | *Holm-adjusted p* |
| --- | --- | --- | --- | --- | --- |
| *Transmission (F)* | 10 | 9 | 0.631 | 0.074 | 0.223 |
| *Transmission (M)* | 10 | 9 | 0.064 | 0.325 | 0.347 |
| *Viral load (F)* | 10 | 9 | 0.708 | 0.010 | 0.041 |
| *Viral load (M)* | 10 | 8 | 0.404 | 0.173 | 0.347 |

*Notes: Positive Mantel r values indicate that closely related isolates had more similar centred profiles. F, female; M, male. Male viral load was analysed using eight host lines because the Au115-DGRP443 combination had no male viral-load estimate; all other analyses used nine host lines. All analyses included ten viral isolates. The phenotypic data underlying this table can be found in S1 Data, and the phylogenetic tree is provided in S3 Data.*

**Table I. Genetic variance components for viral load and transmission using viral isolates collected in the UK**

Models included all nine DGRP host lines and the seven viral isolates collected in the UK: E320, Me44, Ed36, Gr6, Au45, Au115 and Ch384. Estimates are intra-class correlations and therefore represent proportions of total phenotypic variance. Genetic variance (%) gives each component as a percentage of the sum of the host, virus and Host:Virus components within that trait.

| Trait | *Component* | *Estimate (ICC)* | *SE* | *Genetic variance (%)* | *Bound* | *Χ^2^* | *p-value* |
| --- | --- | --- | --- | --- | --- | --- | --- |
| Viral load (M) | Host | 0.197 | 0.114 | 63.1 | P | 6.63 | 0.005 |
| Viral load (M) | Virus | 0.115 | 0.077 | 36.9 | P | 13.15 | <0.001 |
| Viral load (M) | Host:Virus | 0.000 | NA | 0.0 | B | 0.00 | 0.500 |
| Viral load (F) | Host | 0.313 | 0.149 | 48.7 | P | 4.39 | 0.018 |
| Viral load (F) | Virus | 0.105 | 0.079 | 16.3 | P | 17.24 | <0.001 |
| Viral load (F) | Host:Virus | 0.225 | 0.097 | 35.0 | P | 8.27 | 0.002 |
| Transmission (M) | Host | 0.206 | 0.113 | 37.9 | P | 2.21 | 0.069 |
| Transmission (M) | Virus | 0.051 | 0.049 | 9.4 | P | 15.00 | <0.001 |
| Transmission (M) | Host:Virus | 0.288 | 0.068 | 52.8 | P | 228.39 | <0.001 |
| Transmission (F) | Host | 0.045 | 0.048 | 9.1 | P | 6.87 | 0.004 |
| Transmission (F) | Virus | 0.127 | 0.080 | 25.6 | P | 1.83 | 0.088 |
| Transmission (F) | Host:Virus | 0.323 | 0.065 | 65.3 | P | 285.35 | <0.001 |

*Notes: F, female; M, male; P, positive estimate; B, estimate bound at zero. The χ^2^ statistics and p-values are from likelihood-ratio tests against a 50:50 mix of χ^2^ distributions with zero and one degree of freedom. Significance is based on these likelihood-ratio tests rather than the ratio of each estimate to its standard error. The data underlying this table can be found in S1 Data.*
