# Supplementary material for: Host–virus interactions dominate fitness and conceal additive genetic variation
Source: PLoS Biol. 2026 Oct 22;24(10):e3003836. doi: 10.1371/journal.pbio.3003836 (PMC13690250; doi:10.1371/journal.pbio.3003836)
Supplement: Supplementary file 4 [file pbio.3003836.s015.docx]

Dear Dr Roberts,

We thank you and the reviewers for the careful and constructive comments. We have revised the manuscript extensively in response and provide a detailed point-by-point account of the changes below. All manuscript changes are identified using the line numbers in the revised version. The reference list has also been updated to include literature cited in the revised text and to correct incomplete bibliographic records.

**Editorial requests.**

**a) Please address the comments from the reviewers.**

Response: We have addressed all reviewer comments point by point below and revised the manuscript accordingly.

**b) We're finding the current Title a little hard to parse. Please could you consider some more explicit versions - we suggest "Host–virus genotype interactions dominate transmission, virulence and load during infection of Drosophila by sigma virus" or "Host–virus genotype interactions erode additive fitness variance by dominating transmission, virulence and load" (my personal preference would be the latter).**

**Response:** We thank the editor for the suggestion. We have adopted the preferred version, and the title now reads: "Host–virus genotype interactions erode additive fitness variance by dominating transmission, virulence and load". The short running title is unchanged.

**c) Please address my Data Policy requests below; specifically, we need you to supply the numerical values underlying Figs 1ABCDEF, 2ABC, 3ABCDE, 4ABCDE, 5AB, 6ABCD, 7AB, S2ABCDEF, S3ABCDEF, S4ABC, S5ABCD, S6ABCDEFGHIJK, S7ABCDEFGHIJK, either as a supplementary data file or as a permanent DOI’d deposition.**

**Response:** We have provided all underlying numerical values as Supporting Information. S1 Data contains all individual experimental observations and replicates underlying Figs 4–8 and S5 Fig, together with descriptions of the raw and derived variables. The accompanying analysis and figure-generation code shows how the plotted means and errors were calculated. Individual experimental observations underlying Figs 4–8 and S5 Fig are provided in S1 Data, together with descriptions of the raw and derived variables. Simulation outputs underlying Figs 2 and 3 and S2–S4 and S6–S8 Figs are provided in S2 Data, including the per-generation trajectories and parameter-sweep results. Figs 1 and 9 and S1 Fig are schematic and have no underlying numerical data. The sequence alignment and tree underlying the phylogeny in Fig 4 are provided in S3 Data. The variance-component and genetic-correlation estimates are reported in S1 Text. The analysis, simulation and figure-generation code is also archived on Zenodo (DOI: 10.5281/zenodo.21841131).

**d) Please cite the location of the data clearly in all relevant main and supplementary Figure legends, e.g. “The data underlying this Figure can be found in S1 Data” or “The data underlying this Figure can be found in** [**https://zenodo.org/records/XXXXXXXX**](https://zenodo.org/records/XXXXXXXX)**; ; also update your Data Availability Statement accordingly.**

**Response:** We have added the location of the underlying data to every quantitative figure legend. The experimental figure legends refer to S1 Data, the simulation figure legends refer to S2 Data, and the phylogeny in Fig 4 refers to S3 Data. The legends of schematic figures state that they have no underlying numerical data. We have updated the Data Availability Statement accordingly.

**e) Please make any custom code available, either as a supplementary file or as part of your data deposition.**

**Response:** All custom code is deposited on Zenodo (DOI: 10.5281/zenodo.21841131), including the coevolutionary simulation model and the analysis and figure-generation scripts. The Data Availability Statement cites this DOI.

**Reviewer #1:**

This manuscript presents a methodologically ambitious and mathematically rigorous investigation into host-pathogen coevolution. The extensive scale of the experiment and the subsequent application of quantitative genetic models provide a compelling framework for understanding the maintenance of genetic variation. We congratulate the authors on the execution of an experimental undertaking of this magnitude. To ensure the manuscript's conclusions are robustly supported, we suggest the following revisions.

**1) Considering the broad readership of PLoS Biology, the manuscript would benefit from a more accessible conceptual foundation early in the text.** While the manuscript is clearly written, it assumes a high level of specialized prior knowledge regarding quantitative genetics and the decomposition of fitness variance. We recommend including a short paragraph early in the introduction that conceptually defines the genotype-by-genotype interactions and explains why distinguishing between additive and non-additive fitness variance is a critical piece of the "big picture" in evolutionary biology. This will help readers to understand and value the work.

**Response:** We thank the reviewer. We have added a short paragraph at the very start of the Introduction:

**Changes in manuscript, Introduction (lines 46-71):**

“Adaptation by natural selection depends not simply on how much genetic variation a population contains, but on how reliably that variation predicts fitness. Directional selection responses require additive genetic variance which results in differences in mean fitness being predictably passed from parents to offspring. However, if the relative fitness of a genotype depends on the environment it experiences, genotype-by-environment interactions (G×E) can reduce this predictability: a parental genotype that thrives in one setting may fare poorly when expressed by offspring in another. Therefore, there can be abundant genetic variation in phenotype, but little additive variance in fitness. Because selection can act only on consistent differences in mean fitness, such interactions can sometimes slow or even stall the rate of adaptation, even in populations that are genetically variable [1,2]. A biologically important case arises when the relevant ‘environment’ is actually the genotype of another organism [3]. In interactions between males and females [4], competitors, or hosts and their parasites and pathogens (hereafter parasite), the fitness of each partner can depend directly on the genotype of the other [5–7]. This raises the potential for non-additive genotype-by-genotype (G×G) interactions [8] that can reduce the additive genetic variance in the fitness of both parties.

Host–parasite interactions are an excellent model in which to test for G×G effects, because the fitness of each partner depends directly on the genotype of the other: hosts are harmed by their parasites, while parasites must infect a host to survive and reproduce [9]. This mutual dependence makes each party part of the other's selective environment, so that an adaptation in one selects for a counter-adaptation in the other [10]. The resulting antagonistic coevolution has been implicated in the diversification of immune systems [11], the maintenance of sexual over asexual reproduction [12] and the evolution of sexual selection [13], and it can drive ongoing and exceptionally rapid evolutionary change in both host and parasite.”

**2) There is a discrepancy between the text and the data presented in Figure 5 that must be resolved.** Lines 329-331 state that transmission is positively correlated with viral load, reporting positive Pearson correlation coefficient. However, the regression lines plotted in both panels of Figure 5 exhibit clear negative slopes, indicating that higher viral loads correlate with decreased transmission.

**Response:** We thank the reviewer for catching this. The viral-load and fitness panels had been inadvertently swapped during figure assembly. Fig 6 now correctly plots sex-specific viral load against transmission and the plotted relationships, reported correlations and text are consistent.

**3) The host genetic variance is calculated from a non-random subset of nine DGRP lines, which were deliberately chosen to represent specific combinations of major-effect resistance alleles.** The authors must address whether this stratification artificially inflates the perceived genetic and interaction variances compared to what would be observed in a naturally segregating, panmictic population. Furthermore, pairing a localized North American fly population with geographically diverse viral isolates introduces artificial allopatry. The resulting high GH×GP variance may reflect the breakdown of local adaptation rather than the equilibrium state of a sympatric coevolving population. The authors should explicitly discuss these limitations and how sympatric dynamics might alter the ratio of main effects to interaction effects.

**Response:** A related Comment was made below by Reviewer 2 which we address here.

We thank both reviewers. We agree that our panel is not a random sample of host and viral genotypes from a single population at a single time, and that variance components are properties of the set of genotypes from which they are estimated. We have investigated the impact in three ways.

Restricting the host panel. Repeating the analyses using only the five lines susceptible at all three loci leaves G_H_×G_P_ significant and the largest genetic component for transmission in both sexes and for female viral load (Table F in S1 Text). For female transmission the restriction removes almost all of the main-effect variance. The known resistance polymorphisms appear to contribute disproportionately to the main effects; they do not generate the specificity.

Restricting the viral panel. We have now done the equivalent test on the virus side. Seven of our ten isolates were collected in the United Kingdom and this subset is a much closer approximation to a sample from one region than the full panel. G_H_×G_P_ remains the largest genetic component of transmission in both females (65% of genetic variance) and males (53%; Table I in S1 Text). For female viral load it falls to 35%, below the host main effect (49%). The reviewers' concern is therefore well founded for viral replication but not for transmission, the trait on which viral fitness primarily depends.

If genotype specificity arises from the divergence among isolates, closely related isolates should share host specificities. We removed each isolate's mean across the host lines, leaving only its interaction deviations, and compared Euclidean distances between these profiles with patristic distances from the viral phylogeny by Mantel test. Related isolates had more similar interaction profiles for female viral load (r = 0.71, Holm-adjusted p = 0.041), but not for female transmission (r = 0.63, adjusted p = 0.22), male viral load (r = 0.40, adjusted p = 0.35) or male transmission (r = 0.06, adjusted p = 0.35). The two new analyses therefore agree: the composition of the viral panel matters for viral load and not for transmission. Phylogenetic structure in the interaction is not the same as geographical structure, and it is the latter that would compromise our estimates. The best-characterised source of genotype specificity in this system, sensitivity to the *p62* resistance allele, is phylogenetically clustered because the escape type arose recently and spread as a lineage, yet sensitive and escape types coexist within single natural populations. Lineage-associated specificity is therefore expected within a panmictic population and does not require the geographical mixing of our panel.

We nonetheless agree that our estimates characterise the panel we assembled rather than a population at coevolutionary equilibrium, and because no isolate came from the DGRP source population we cannot say what the balance of main and interaction variance would be in a sympatric sample. The Discussion now states this directly.

Finally, the allopatry in our design may matter. However, *D. melanogaster* is a human commensal with high gene flow, the North American population from which the DGRP derives was founded by immigration from Europe in the late 19th century, and European and North American DMelSV isolates share a common ancestor only ~200 years ago. The known resistance polymorphisms segregate on both continents. The combinations we assayed therefore likely occur in nature; they are not pairings of long-separated coevolutionary lineages.

**Changes in manuscript, Methods (lines 938–942):**

“We repeated the transmission and viral load models using two restricted datasets: first, only the five DGRP lines susceptible at all three known resistance loci (303, 443, 712, 801 and 850), retaining all ten viral isolates; and second, all nine DGRP lines but only the seven viral isolates collected in the UK (E320, Me44, Ed36, Gr6, Au45, Au115 and Ch384).”

**Changes in manuscript, Methods (lines 962–969):**

“Additionally, we tested whether closely related viruses had similar patterns of host-specific effects across fly lines. For transmission and viral load in each sex, we subtracted each virus's mean across fly lines from its value in every line, removing the virus main effect. We then calculated Euclidean distances between these sets of deviations for each pair of viruses and compared them with phylogenetic distances, measured as the summed branch lengths between each pair of viruses, using one-sided Mantel tests with 9,999 permutations. We also adjusted *p* values across the four tests using Holm’s method.”

**Changes in manuscript, Results (lines 317–322):**

“These interactions persisted when the analysis was restricted to the five susceptible lines, although both the interaction and host main-effect estimates were lower and less precise with fewer lines (Table F in S1 Text). Restricting the analysis to the seven UK viral isolates likewise left G_H_×G_P_ as the largest genetic component of transmission in both females (65%; ICC = 0.32 ± 0.07 SE, p < 0.001) and males (53%; ICC = 0.29 ± 0.07 SE, p < 0.001; Table I in S1 Text).”

**Changes in manuscript, Results (lines 375–381):**

“Restricting the analysis to the five susceptible lines gave the same pattern (Table F in S1 Text). With the seven UK isolates, G_H_×G_P_ remained undetectable in males and significant in females, but in females it fell to 35% of the genetic variance (ICC = 0.22 ± 0.10 SE, *p* = 0.002), below the host main effect (ICC = 0.31 ± 0.15 SE, *p* = 0.018; Table I in S1 Text). Therefore, the interaction variance in female viral load depends on the viral genotypes assayed, although estimates from only seven isolates are less precise.”

**Changes in manuscript, Results (lines 427–436):**

“To examine whether the G_H_×G_P_ interaction reflected viral evolutionary history, we tested whether related isolates shared similar host-specific effects. For each trait, we removed each isolate’s mean and compared Euclidean distances between the resulting G_H_×G_P_ profiles with patristic genetic distance on the viral phylogeny (Fig 4A) across the 45 pairs of isolates. Closely related isolates had more similar G_H_×G_P_ profiles for female viral load (Mantel *r* = 0.71, Holm-adjusted *p* = 0.041). Female transmission showed a positive but non-significant relationship (*r* = 0.63, adjusted *p* = 0.223), while neither male viral load (*r* = 0.40, adjusted *p* = 0.347) nor male transmission (*r* = 0.06, adjusted *p* = 0.347) was significantly associated with phylogenetic relatedness (Table H in S1 Text).”

**Changes in manuscript, Discussion (lines 560–581):**

“Two features of our experimental design could in principle have influenced how variance was partitioned: the host lines were chosen around three major-effect resistance polymorphisms, and the viral isolates came from geographically separated populations rather than from the source population of the host lines. Neither fully explains the interactions in transmission, the trait on which viral fitness primarily depends. G_H_×G_P_ remained the largest genetic component of transmission in both sexes when either the host panel was restricted to lines carrying the susceptible allele at all three loci or the viral panel was restricted to isolates from the UK (Tables F and I in S1 Text). We also detected no relationship between the G_H_×G_P_ specificity of transmission and viral phylogeny. Female viral load behaved differently as restricting the viral panel to the UK reduced its interaction variance below the host main effect (Table I in S1 Text). However, the G_H_×G_P_ interaction variance remained substantial.

Geographical structure will affect our estimates. The best-characterised source of genotype specificity in this system is the sensitivity of viral genotypes to the *p62* polymorphism. This may in part explain why G_H_×G_P_ effects were clustered on the viral phylogeny, as the escape type arose and spread recently. However, both the host and viral polymorphisms are found within multiple natural populations [44–46]. What our data cannot establish is the precise balance of main and interaction variance that would be found in a population sampled at one place and time. Obtaining that estimate would require contemporaneous samples of host and viral genotypes from the same population.”

We added three supplementary tables:

“S1F Table. Genetic variance components from analyses restricted to five host lines susceptible at all three major-effect resistance loci.”

“S1H Table. Association between viral phylogenetic distance and host-specific effects on transmission and viral load.”

“S1I Table. Genetic variance components for viral load and transmission using viral isolates collected in the UK.”

Table F reports the restricted host-line analysis, Table H reports the Mantel tests of viral relatedness and host-specific effects, and Table I reports the variance components obtained using the seven viral isolates collected in the UK.

**4) The mathematical model detailed in Figure 8 contains a rigid biological assumption regarding the developmental trajectory of the virus.** The model dictates that all surviving infected zygotes inevitably develop into infected adults, subject only to the viability cost parameter c. This formulation assumes a zero percent viral clearance rate during host development. The authors should clarify whether complete viral persistence through metamorphosis occurs for the sigma virus. If the host can clear the infection during larval stages, the authors should consider incorporating a clearance parameter into the model, or at least discuss how virus clearance might alter the predicted coevolutionary oscillations.

**Response:** We thank the reviewer for this point. Our original model assumed complete persistence of infection through development. Sigma virus infections are generally considered persistent, and we are not aware of direct evidence that infected flies clear the virus during larval development or metamorphosis. However, virus loss has not been quantified directly.

Vertical transmission is measured from infected adults to their adult offspring. These measurements therefore do not distinguish failure to transmit the virus to gametes from loss of infection during offspring development. We therefore performed a separate analysis in which we moved the virus-loss event to three positions in the life cycle: during transmission to gametes, immediately after fertilization but before viability selection, and after viability selection but before the adult stage. When loss occurred during transmission, resistance acted according to parental genotype. When it occurred after fertilization, resistance acted according to the offspring genotype. We repeated the GFG1 parameter sweep using the parameter ranges and initial condition of Fig3, with each combination simulated for 10,000 generations.

Moving virus loss to after viability selection produced the same outcome classification as the original model in 286 of 288 parameter combinations. Placing virus loss before viability selection resulted in stronger damping and shifted the boundaries among dynamical regimes. However, the qualitative conclusions remained: damped and persistent oscillations were possible under all three timings. The predicted coevolutionary oscillations are therefore robust to uncertainty about the stage at which virus loss occurs, although the timing can affect their strength.

We retained the original model in the main analysis and provide the full comparison analysis in S1 Text and S8 Fig, with the underlying data in S2 Data.

**Changes in manuscript, Results (lines 235-241):**

“In our experiments, vertical-transmission rates are measured between adult generations, so they do not reveal whether virus loss occurs during transmission to gametes or during offspring development. To check our assumptions are not affecting our conclusions, we explored the effects of viral loss at different stages in the life cycle. While these changes did alter the boundaries where the dynamics shifted between regimes, oscillatory dynamics were consistently present and our qualitative conclusions remained (S8 Fig; S1 Text).”

**Changes in S1 Text:**

Vertical-transmission rates are measured from infected adults to their adult offspring and therefore do not identify the stage at which virus loss occurs. In the original model, all incomplete transmission occurs during the production of gametes, and infected zygotes that survive the infection-induced viability cost remain infected until the adult census. We examined whether this assumption affected the coevolutionary dynamics by moving the existing virus-loss event to different stages of the life cycle, without adding an additional source of loss.

For virus *i* and host genotype *G*, the effective probability of retaining the virus was $t_{i,G}=t_{i}\left( 1-r\cdot1_{i,G} \right)$, as in the original model, where 1*_i,G_*=1 if host genotype *G* is resistant to virus *i*, and 0 otherwise. The complementary fraction 1‑ *t_i,G_* lost the virus and entered the uninfected class while retaining the same host genotype.

We compared three positions of virus loss. In the original model, loss occurs during transmission to gametes and resistance acts according to the genotype of the infected parent. In the first alternative, loss occurs immediately after fertilisation but before viability selection, with resistance acting according to the offspring genotype. Zygotes that lose the virus therefore avoid the infection-induced viability cost *c*. In the second alternative, infected zygotes first experience the viability cost; among those that survive, virus loss then occurs before the adult census, again with resistance acting according to the offspring genotype.

The GFG1 parameter sweep was repeated for all three models using the parameter ranges and initial conditions of Fig 3, with each parameter combination simulated for 10,000 generations. The models were compared using the coefficient of variation of viral-genotype frequencies and the resulting classification of coevolutionary dynamics. Moving virus loss to after viability selection produced the same outcome classification as the original model in 286 of 288 parameter combinations. Loss before viability selection resulted in stronger damping and shifted the boundaries among dynamical regimes, but persistent oscillations remained possible under all three timings (S8 Fig).

**5) We strongly recommend expanding the Discussion to address whether these findings can be extrapolated to horizontally transmitted viruses.**

**Response:** We thank the reviewer and have added a paragraph to the Discussion addressing this:

**Changes in manuscript, Discussion (lines 652-682):**

“Although the concentration of fitness variance in G_H_×G_P_ may partly reflect features specific to sigma virus, the dynamics and genotype-specific effects that generate this pattern in our models are not restricted to vertically transmitted parasites. For example, temporal studies of *Daphnia*-*Pasteuria* and wild flax–flax rust found reciprocal cycling in host resistance and parasite infectivity through time [93,94], while genomic evidence from *Daphnia* resistance loci indicates that these polymorphisms can persist over long timescales [95]. Fluctuating selection and the maintenance of polymorphisms are therefore common in horizontally transmitted parasites. Furthermore, G_H_×G_P_ interactions are widespread across many host and parasite traits [6,7]. However, experiments in other systems typically do not decompose variance components for both parties, and studies of horizontally transmitted pathogens often measure traits, such as parasite burden or infectivity, which may not reliably reflect fitness. For example, the burden of the parasite *Ordospora colligate* in *Daphnia* did not reliably predict estimates of fitness from persistence in experimental populations across time [96]. A notable exception is the monarch butterfly–*Ophryocystis* system, where host and parasite genotypes interact to determine virulence and transmission in wild populations (although here the variance was not decomposed into main and interaction components) [6]. In our system, the number of infected offspring provides a simple measure of parasite fitness because the virus is vertically transmitted, and multiple host traits are relatively easily measurable.

The balance of variance components is also contingent on whether coevolution sustains genotype specificity (G_H_×G_P_) or favours broadly effective resistance and infectivity (main effects). This may be system specific, with broadly infective phages evolving during bacteria-phage coevolution [97], and general host resistance emerging in the arms race between an alga and its DNA virus [98]. Other features of viral biology may matter too: reassortment or recombination can move compatibility alleles between viral backgrounds, whereas sigma virus is non-recombining. Therefore, while we would expect our results to hold qualitatively across many other systems, quantitatively, the extent to which the concentration of fitness variance in G_H_×G_P_ extends beyond this system remains an open and testable question.”

**Reviewer #2:**

This study investigates the effects of host-pathogen coevolution by negative frequency-dependent selection (NFDS) on genetic variation in both host and pathogen. Specifically, the authors estimate what fraction of the genetic variation in virulence and transmission that is heritable vs bound in host genotype-by-pathogen genotype interactions (G*G). Interestingly, the authors found that most of the variation is in the G*G variance component. They also found strong G*G*sex interactions which is intriguing; cannot think of any previous studies that have shown that. The authors use a novel approach to a question that is of broad interest. The paper is very well written and the presentation is clear.

**The only major issue I can see concerns the sampling design of host and, especially, pathogen genotypes.** NFDS is a process that occurs within populations. To estimate the consequences of NFDS (as regards e.g. variance components) one should therefore have random samples of host and pathogen genotypes from a given population at a given point in time. Here, the authors have rather tried to maximize the genetic diversity, especially for the virus where samples are from both Europe and North America (and no isolate seems to be from where the fly lines were originally collected). Thus, the pathogen in particular is likely much more genetically diverse in this study than what is the case in a natural population. Also, it is not matched to the host population. I suspect that this might inflate the G*G variance component. I understand that it would be a daunting task to collect both fly and virus lines from a given population, but this limitation of the study should still be acknowledged and discussed (the authors touch on this on L 460-467 but I think the potential problem with the sampling design needs to be made more clear).

**Response:** see Reviewer 1, comment 3.

**Other relatively minor comments/questions:**

**L 23-24 & L 406-407**: Is it NFDS that leads to G*G (as implied by wording on L 23-24) or G*G that leads to NFDS (as implied by wording on L 406-407)? I would say the latter, but guess it depends on whether you think of G*G "mechanistically" (where G*G->NFDS) or the G*G variance component (in which case NFDS drives G*G variance). In any case, I think it would be good to use a consistent perspective throughout the ms.

**Response:** Our grammar made this point ambiguous in the abstract. We now consistently state that G×G interactions can generate negative frequency-dependent selection. We reworded the abstract sentence.

**Changes in manuscript, Abstract (lines 22-28):**

“Genotype-by-genotype interactions on fitness can generate negative frequency-dependent selection, which helps maintain this variation by preventing any host or pathogen genotype from retaining a permanent advantage. Because these interactions place genetic variance in the interaction term rather than in additive differences among hosts or pathogens, that variance is masked from directional selection and can remain 'cryptic'.”

**L 34**: With the experimental design used here (inbred lines), I believe the main effect of host estimates the broad-sense heritability, i.e. not only additive but also dominance and epistasis, right? And I think it would be same for the virus. If so, this should be clarified throughout.

**Response:** Thank you for pointing out that this is not mentioned. We have clarified the interpretation of the variance components separately for the experimental analysis and the theoretical model. In the experimental analysis, the host and virus terms are main-effect variances; their genetic interpretation is explained in the Results. In the theoretical model, *V_G(host)_* and *V_G(virus)_* denote the host and virus main-effect variances, while *V_GxG_* denotes the interaction variance.

**Changes in manuscript, Results (lines 268-275):**

“As the virus is haploid and non-recombining, its genome is transmitted clonally. There is no dominance, and any epistasis among viral loci is inherited intact and so contributes solely to the additive component of genetic variance. The virus main-effect variance is therefore an estimate of the additive genetic variance. The host lines are inbred, so the main-effect variance will capture non-additive (dominance and epistatic) variance. Nevertheless, most genetic variance is theoretically expected to be additive [69], so this term should predominantly reflect additive genetic variance.”

**Changes in manuscript, Methods (lines 931-938):**

“First, we fitted a univariate model to each of the six traits, with no fixed effects specified and host identity, virus identity, and their interaction fitted as random effects. For fecundity and hatch rate, the line-specific mean of uninfected flies was fitted as a fixed covariate and only infected flies were included as responses. This yielded estimates of the host and virus main-effect variances, and variance attributable to G_H_×G_P_ interactions. We scaled all three variance components to intra-class correlations (i.e. proportions of total phenotypic variance).”

**L 58**: "pathogens targeted by that [host] allele" implies infection matrix of the Gene-For-Gene type (or inverse Matching Allele), while in Matching Allele it is the pathogen that targets the host. Since your analysis is agnostic to whether the G*G is of GFG or MA type, I suggest using another word than "target" that is neutral to the direction of the interaction.

**Response:** We have replaced "targeted by that allele" with the direction-neutral "affected by that allele."

**Changes in manuscript, Introduction (lines 79-82):**

“When resistance alleles spread through a population, the prevalence of pathogen genotypes affected by those alleles can decline to a point where these costs outweigh the benefits of resistance [8].”

**L 131-132**: The minor allele frequency of the host polymorphism is surprisingly low (<0.1) for a balanced polymorphism. It seems unrealisitc that this can maintained in the long term in the face of drift, or? Perhaps worth a comment and/or comparison with previous similar models.

**Response:** We agree that the deterministic model does not capture the risk that drift removes alleles when they become rare. We discuss this in a new paragraph in the Discussion.

**Changes in manuscript, Discussion (lines 516-525):**

“Our model is deterministic, describing dynamics in the absence of drift. Negative frequency-dependent selection opposes the loss of rare alleles, but our models predict that in some parameter space alleles become transiently rare. Here, persistence will depend on the effective population size [73,74]. In small or strongly subdivided populations, host and virus alleles may fix, eliminating the variation on which the interaction term depends. If fixation occurs locally, coevolution may generate differences between rather than within populations, in which case whether G_H_×G_P_ dominates fitness variance is in part a demographic question. However, in our system we know the resistance and viral polymorphisms segregate within wild populations.”

**L 154-155 & fig 1**: I think it would be good to include a fig specifying the infection matrix and explain if this corresponds to GFG or MA (or perhaps inverse GFG/MA).

**Response:** We thank the reviewer. We have added S1 Fig showing the infection matrix for each model, and now describe the one-locus gene-for-gene (GFG1), two locus variant of gene-for-gene (GFG2) and matching-allele (MA) models in the Methods.

**Changes in manuscript, Results (lines 161-162):**

“In line with classical gene-for-gene models (S1 Fig), both host resistance and viral escape incurred costs [65].”

**Changes in manuscript, Methods (lines 768-769):**

“Three coevolution models were implemented (S1 Fig).”

**Changes in S1 Text, Interaction matrices:**

“S1A Table. Resistance indicators 11,H, 12,H and dominant-allele locus count D(H) for each of the nine diploid host genotypes, under the three genetic models (GFG1, GFG2, MA). The selective sweep model uses the GFG2 column entries throughout and sets d = 0.”

**L 329-331**. Explain how the negative slopes in fig 5 correspond to positive correlations between transmission and viral load.

**Response:** We thank the reviewer for catching this. The viral-load and fitness panels had been inadvertently swapped during figure assembly. Fig 6 now correctly plots sex-specific viral load against transmission, and the plotted relationships, reported correlations and text are consistent.

**L 378-379**: It is not clear to me why this would reflect tolerance rather than resistance. As you did not include load as a covariate you cannot separate resistance and tolerance, or am I missing something?

**Response:** We thank the reviewer, and we agree. We have removed the tolerance interpretation from the Results. Tolerance is discussed later only as a possible explanation.

**Changes in manuscript, Results (lines 464-468):**

“The main effect of viral genotype did not significantly influence hatch rate or fecundity (Fig 7D), indicating no detectable effect of viral genotype on host reproduction at the population level. Host genotype effects were similarly weak for hatch rate (ICC = 0.011 ± 0.015 SE, *p* = 0.16) but accounted for 40% of the total genetic variance in fecundity (ICC = 0.096 ± 0.056 SE, *p* < 0.001, Fig 7D).”

**Changes in manuscript, Discussion (lines 631-639):**

“One possible explanation for this decoupling is tolerance: host genotypes may differ in the reproductive loss they suffer at a given viral burden, so that higher replication need not translate into greater harm. Genetic variation in fecundity tolerance has been documented in insects [20], including *D.* *melanogaster* infected with *Drosophila* C virus [89]. Under vertical transmission, this could benefit both partners because the virus depends on host reproduction for transmission [71,72]. Tolerance that preserves fecundity therefore also preserves opportunities for viral transmission, potentially weakening selection against otherwise costly infections [55,56].“

**Reviewer #3:**

General comment: The integrated approach combining simulations and experiments to investigating negative frequency-dependent selection (NFDS) makes this study quite interesting for the host/viral interactions field. The authors first established explicit theoretical expectations using coevolutionary models and then test these predictions experimentally across a large host-virus factorial design. The results and inferences derived from the D. melanogaster-Sigma virus system are likely to provide a useful framework for investigating genotype-by-genotype interactions and coevolutionary dynamics in other host-virus systems, generating testable hypotheses that can be evaluated across a broader range of biological models.

**The authors appropriately acknowledge that many genetic correlation estimates are associated with large standard errors and should be interpreted cautiously (lines 336-337 and 501-504).** **Nevertheless, some sections of the Results still draw biological conclusions from rather imprecise estimates.** For example, the discussion of sex-specific transmission architecture (lines 253-261) interprets host and virus cross-sex genetic correlations despite substantial uncertainty around these estimates (host: rg = 0.19 ± 0.46; virus: rg = 0.57 ± 0.39).

**Response:** We thank the reviewer. We have revised these sections to distinguish the comparatively precise interaction correlations from the poorly estimated host and virus main-effect correlations, and have softened the biological interpretations accordingly.

**Changes in manuscript, Results (lines 328-329):**

“The cross-sex correlations of the viral and host main effects were poorly estimated (virus: 𝑟_𝑔_ = 0.54 ± 0.44 SE; host: 𝑟_𝑔_ = 0.17 ± 0.46 SE).”

Similarly, the conclusions regarding shared genetic control of viral replication between sexes (lines 303-308) are based on a host genetic correlation estimated as rg = 0.71 ± 0.39.

**Changes in manuscript, Results (lines 381-386):**

“The cross-sex correlations of the host and viral main effects were positive but imprecisely estimated (main effect host genotype: 𝑟_𝑔_ = 0.76 ± 0.43 SE; the viral estimate was bound near one, with the among-virus variance close to zero, and is not interpretable; Table D in S1 Text). These estimates are consistent with some shared genetic influences on viral load across sexes, but do not establish their strength.”

Finally, the interpretation of viral load-transmission relationships (lines 336-343) relies on several genetic correlations with large standard errors (e.g., host effect in males: rg = 0.52 ± 0.42; host effect in females: rg = 0.46 ± 0.34). **I suggest the authors to consider further softening these interpretations.**

**Changes in manuscript, Results (lines 414-419):**

“In females, the interaction term showed a positive genetic correlation (host-virus interaction 𝑟_𝑔_ = 0.42 ± 0.15 SE; all between-trait genetic correlations are reported in Table C in S1 Text). The main effects in females and all effects in males were poorly estimated (Table C in S1 Text). Therefore, despite a lack of statistical power for most correlations, the female interaction estimate suggests partly shared genetic determination of viral load and transmission.”

**750-751:** "A large sex-specific difference in RpL32 expression was detected, requiring sex- specific normalization" **Is it expected for D. melanogaster? Another gene could have provided better normalization for this experimental design?**

**Response:** Yes, this difference is expected. RpL32 (Rp49) is normally expressed at higher levels in females than males in D. melanogaster (for example, FlyAtlas 2 reports a whole-body male-to-female expression ratio of about 0.6). It is also the standard reference gene for this species. Because sex is a major factor in gene expression in Drosophila, reference genes commonly show some sex bias, although another reference gene might show less sex bias and would require separate validation. However, this difference does not affect our within-sex variance-component estimates: we mean-centred RpL32 Ct within each host-genotype × virus-genotype × sex combination and analysed viral load as a sex-specific trait, so the difference in mean expression between the sexes does not enter the variance-component estimates on which our conclusions are based. We have added a note to this effect in the Methods and cited FlyAtlas 2.

**Changes in manuscript, Methods (lines 917-926):**

“A large sex-specific difference in *RpL32* expression was detected, requiring normalization that accounted for sex. This difference is expected in *D. melanogaster*: *RpL32* (*Rp49*) is normally expressed more highly in females than males (FlyAtlas 2; [107]). Normalizing within each sex removes this baseline difference from the viral load estimates. *RpL32* Ct values were mean-centred within each host genotype × virus genotype × sex combination, and the overall mean *RpL32* Ct was added back to retain the original Ct scale and allow comparison with the sigma virus Ct values. Normalized ΔCt, representing relative viral RNA load, was calculated as the mean-centred *RpL32* Ct minus the Ct of the targeted sigma virus gene. Relative viral RNA load corresponds to log_2_ viral load.”

**The Discussion is quite long and often repeat the results.**

**Response:** We agree and have revised the Discussion to reduce repetition of the Results. We removed the opening recap of the experimental design, condensed the summary of the theoretical results, deleted a paragraph that relied on imprecisely estimated genetic correlations, and removed the repeated numerical comparison of male and female transmission. We also made smaller cuts and edits throughout. New material added in response to other reviewers focuses on the sampling design, limitations and generalisation of the results.

**Main changes in manuscript, Discussion:**

We deleted the paragraph beginning “Next, we carried out a large-scale study with more than 4000 flies …”

We replaced the paragraph beginning “The dynamics are highly sensitive to parameter choice…” with the following more concise summary (lines 506–508): “Although the dynamics were sensitive to parameters, ranging from damped to persistent oscillations, fitness variance for both host and virus was consistently dominated by the *V_G×G_* term.”

We deleted the paragraph beginning “Unlike transmission, viral load showed a strong phenotypic correlation and relatively high genetic correlation between males and females...”.

We deleted the sentence “For transmission and female viral load, genetic effects collectively explained more than half of the phenotypic variance, with GH×GP accounting for much of this contribution.”

We deleted the sentence “Infected females transmitted the virus to 85% of their offspring on average, compared to 26% for infected males.”

**While the authors appropriately focus on the Drosophila-sigma virus system, the Discussion could more explicitly acknowledge the extent to which the observed patterns may depend on the biology of sigma virus itself.** Sigma virus is a vertically transmitted, host-specialized negative-sense RNA virus, and its coevolutionary dynamics may differ substantially from those of horizontally transmitted viruses, segmented RNA viruses capable of reassortment, or large DNA viruses with different evolutionary constraints. **A brief discussion of the potential limits to generalizing the observed dominance of GH×GP interactions across other host-virus systems would also strengthen the manuscript.**

**Response:** We agree. This comment overlaps with Reviewer 1, comment 5. In response to both comments, we added a paragraph discussing the limits to generalising the quantitative dominance of GH×GP beyond sigma virus. It considers evidence from horizontally transmitted systems and explains why transmission mode, viral genome architecture and the nature of genetic interactions could alter the balance between main and interaction effects.

**Reviewer #4:**

This manuscript describes an ambitious and rigorous study that combines theory and experiment to address important questions in the biology of host-parasite coevolution. The results are fascinating and I learned a great deal from reading the manuscript. I expect that this work will ultimately become a classic of coevolutionary biology.

My absolute favorite bits include the rigor and clarity of the evidence of GxG for transmission, as well as the compelling evidence for sex specificity in many of the genetic and GxG effects. In general, I find the empirical results considerably more interesting and compelling than the theory but can see that the theory may fill a gap in the literature on vertically transmitted parasites while also explicitly tracking GxG variance.

I have several suggestions to improve the clarity and impact of the manuscript, as follows.

**Suggestions for revision:**

**1)	Clarify/broaden the framing to acknowledge the diversity of modes of selection.**

In many places, the narrow evolutionary framing was jarring. For ex, the sentence about an "evolutionary response" (p. 6 lines 118-119) implies that balancing selection is not evolution. Perhaps instead specify directional selection here? Similarly, on p. 25 lines 527-528: "coevolution masking heritable variation from the effects of natural selection" could equally be framed as "coevolution dynamically maintaining heritable variation via natural selection." I take your point that net fitness advantages of a given G disappear, even if there are transient advantages. But I don't see the need for an exclusive focus on directional selection so suggest also acknowledging diversity-promoting balancing selection.

**Response:** We agree, and thank the reviewer. We have rewritten these sections:

**Changes in manuscript, Abstract (lines 22-28):**

“Genotype-by-genotype interactions on fitness can generate negative frequency-dependent selection, which helps maintain this variation by preventing any host or pathogen genotype from retaining a permanent advantage. Because these interactions place genetic variance in the interaction term rather than in additive differences among hosts or pathogens, that variance is masked from directional selection and can remain 'cryptic'.”

**Changes in manuscript, Abstract (lines 38-40):**

“Our results demonstrate that coevolution can substantially mask heritable genetic variation from directional selection by rendering fitness context dependent.”

**Changes in manuscript, Introduction (lines 145-148):**

“Thus, despite extensive genetic effects on infection outcomes, coevolution has reduced heritable variation in fitness for both host and pathogen, potentially limiting the response to directional selection.”

**Changes in manuscript, Discussion (lines 686-690):**

“Our results show that, in a natural vertically transmitted virus system, genetic variance across a suite of host and virus traits was dominated by the V_G×G_ interaction term, consistent with coevolution masking heritable variation from directional selection while maintaining it through negative frequency-dependent (balancing) selection.”

**2)	Explain figures more fully and clearly.** The manuscript includes many figures of considerable complexity and in several cases, the explanations were insufficient for me. I was especially baffled by the apparent disconnect between the description of figure 5 in the text and the appearance of both panels: the relationships do not look positive, and indeed, if correlated at all, the data suggest a negative relationship (e.g., a cost of virulence?). Please check and clarify? perhaps phenotypic correlations obscure the direction of the genetic correlations? Or did I miss a key point here? I also think that Fig 2 needs more explanation, especially of the curved region (surrounding the diagonal cost line) that is associated with higher CV and AGV. Does that range of cost balances somehow alter GxG impacts?

**Response:** We thank the reviewer. For the former Fig 5, now Fig 6, the mismatch was caused by an accidental panel swap during figure assembly, which we have corrected (as noted in our replies to Reviewer 1); the panels, the reported correlations and the text now agree.

For the former Fig 2, now Fig 3, we have added further explanation in the Results and figure legend.

**Changes in manuscript, Results (lines 213-226):**

“A higher coefficient of variation marks parameters where the polymorphism persists through large, ongoing allele-frequency cycles (Fig 3A). These cycles repeatedly shift variance onto the host and virus main effects, so the additive share of fitness variance is higher when genotype frequencies fluctuate more (Fig 3B and 3C). The extent to which genotype frequencies fluctuate depends on the costs of infection and resistance (Fig 3A), and this relationship in turn changes depending on other model parameters (S6 Fig and S7 Fig). Even so, when averaged over time the interaction term was larger than the corresponding additive component in most, but not all, parameter combinations that maintained polymorphism (Fig 3B and 3C; S6 and S7 Fig). Additive components could also briefly exceed the interaction component within individual generations. Thus, genotype‑by‑genotype interactions arising during coevolution are expected, in most conditions, to substantially depress additive genetic variance in host and virus fitness.”

**Changes in Fig 3 legend (lines 248-263):**

Rewritten to include more detail.

**3)	Deepen discussion of all results, whether or not they align with the GxG-centred framing.** The hard-won results seem under-discussed in various ways. If space permits, I suggest some deepening of the Discussion section. For ex, inspired by p. 11 lines 217-219: this is totally fascinating and seems a promising area for future study. Perhaps elaborate on how to investigate potentially sex-specific mechanisms and what we might learn from such studies? I also think it is important for the authors to better probe the relative lack of GxG for fly fecundity and hatch rate: For ex, on p. 22 lines 447-450, this inference doesn't sit quite right, in part because "dominates" doesn't seem right if only ~6-10% of variance is explained, and if host main effects can rival the explanatory power of GxG for fecundity. What unmanipulated, unmeasured traits in this study influence fecundity and hatch rate? The Discussion needs to better capture these interesting nuances. The message of GxG impacts still will come across! Finally, you mention the possibility of tolerance as an explanation in the results but don't go there in the Discussion - please do?

**Response:** We thank the reviewer for these suggestions, which have helped us deepen the Discussion. We have trimmed repetition elsewhere so that the section has not grown substantially, and we address each point below.

Reviewer 4, comment 3 “Perhaps elaborate on how to investigate potentially sex-specific mechanisms and what we might learn from such studies?”

**Response:** We agree this is a promising direction. We now greatly expand this section of the Discussion:

**Changes in manuscript, Discussion (lines 582-614):**

“Sex is among the most pervasive sources of variation in infection outcome. Males and females differ in susceptibility, immune signalling and pathology in animals from insects to humans [83,84], and in *Drosophila* these differences are associated with distinct behavioural, epithelial, cellular and systemic defences [84]. Consistent with prior research, we found the sigma virus is a striking example of this, with strong sex differences in transmission rates [14,30]. This likely results from the virus having different transmission routes in males and females, through ovaries and oocytes versus the male germline and sperm. Infection-induced transcriptional responses also differ between the sexes [85]. Quantifying viral load directly in ovaries, testes and gametes across host-virus combinations could test whether sex-specific transmission reflects differences in reproductive-tissue infection rather than whole-body viral load. While maternal transmission is the primary route of sigma virus spread, male transmission is essential for viral persistence at the population level: in its absence, the fitness costs of infection would gradually reduce the viral prevalence [33,57,68]. The variance in transmission is similar in both males and females. As there is expected to be selection on the host to reduce transmission to offspring and the virus to increase transmission in both sexes, there are comparable influences of maternal and paternal transmission on fitness variance.

Work on the sexual dimorphism of infection outcomes typically treats sex as a main effect, asking whether one sex is more resistant or has a different immune response. It is less understood whether the specificity of host–pathogen interactions is sex-specific. Our results demonstrate that the identity of the host-pathogen genotype combinations where infection succeeds differ between the sexes. The host–virus interactions in transmission were only weakly shared across the sexes (cross-sex *r_g_* = 0.34 ± 0.12 SE). Maternal and paternal transmission are therefore two distinct traits with partly separate genetic and mechanistic bases, not one trait expressed twice. This means that selection on one party will not optimise both routes of transmission, because a viral genotype with high transmission in eggs is only weakly predictive of transmission in sperm, and a host genotype that blocks transmission through eggs may not prevent transmission through sperm. This means sex becomes a further source of context dependence alongside partner genotype, dispersing genetic variance still further from the main effects on which directional selection acts.”

Reviewer 4, comment 3:”I also think it is important for the authors to better probe the relative lack of GxG for fly fecundity and hatch rate…"

**Response:** We agree. We softened the general statement so it no longer implies dominance for reproductive traits, and we added a paragraph discussing why the total genetic contribution is smaller there, drawing on sigma-specific evidence that whole-body titre does not consistently predict reproductive harm. We also clarify in the Results that much of the phenotypic variation remained outside the measured genetic effects. Because our design cannot identify the individual or environmental sources of this residual variation, we avoid attributing it to particular unmeasured traits. We also state explicitly that reproductive responses were not uniformly negative and treat these apparent increases as an open question.

**Changes in manuscript, Discussion (lines 535-537):**

“We find G_H_×G_P_ was the largest genetic component for every trait except male viral load. For host reproductive traits, however, that genetic component was itself small.”

**Changes in manuscript, Discussion (lines 615-627):**

“For fly life-history traits, although G_H_×G_P_ remained the largest genetic component, the host main effect was of comparable magnitude for fecundity, indicating consistent differences among host lines in their reproductive response to infection. Previous sigma virus studies reported lower egg production in infected flies [33] and reduced egg viability but no significant overall effect on male or female fertility [66], although neither examined combinations of host and viral genotypes. Intriguingly, despite well-documented fitness costs of sigma infection [33,34,39,68,86,87], the reproductive effect of infection was not uniform but depended on the particular host–virus combination and sometimes led to increases in female reproductive output, mirroring weakly supported reports of minor reproductive gains in infected males [66,88]. Whether these apparent increases represent reproducible biological responses to infection, and whether they occur in natural populations, remains unclear.”

Reviewer 4, comment 3: “Finally, you mention the possibility of tolerance as an explanation in the results but don't go there in the Discussion…”

**Response:** We agree and now discuss tolerance as a possible explanation in the Discussion, where the decoupling between viral load and reproductive harm is presented:

**Changes in manuscript, Discussion (lines 631-639):**

“One possible explanation for this decoupling is tolerance: host genotypes may differ in the reproductive loss they suffer at a given viral burden, so that higher replication need not translate into greater harm. Genetic variation in fecundity tolerance has been documented in insects [20], including *D.* *melanogaster* infected with *Drosophila* C virus [89]. Under vertical transmission, this could benefit both partners because the virus depends on host reproduction for transmission [71,72]. Tolerance that preserves fecundity therefore also preserves opportunities for viral transmission, potentially weakening selection against otherwise costly infections [55,56].”

**4)	Explain the system better for a wide audience who will largely be unfamiliar with it.** For a generalist audience, a bit more explanation of the system in the main text might help outsiders understand the biology as well as your methods. For ex, why are the viruses at such low prevalence, if solely vertically transmitted? Is it because they tend to be virulent (in other studies, if not this one)? In the Methods: why do you need a "balancer" stock? might your requirement for "stable transmission rates" (p. 32 lines 672-673) have made it inevitable that you would not observe selective sweeps? A figure to illustrate the complexities of the line establishment and experimental design might also be useful to convey system info and thus strengthen reader confidence in the approaches and findings.

**Response:** We thank the reviewer and have added explanation of the system for a general readership. The Introduction now identifies sigma virus as a negative-sense RNA virus (Rhabdoviridae) and explains why incomplete transmission and the fitness costs of infection maintain it at low prevalence, with paternal transmission contributing to its persistence. In the Methods, we now state the purpose of the balancer stock and clarify that “stable transmission” refers to stabilisation of the infection before phenotypic testing. We have also removed the potentially misleading reference to “stable transmission rates.” The experiment measured fixed host–virus combinations and was not designed to observe selective sweeps through time; these dynamics were examined in the model (S4 Fig). We added Fig 1 to illustrate sigma virus transmission and the experimental design.

**Changes in manuscript, Introduction (lines 110-118):**

“The sigma virus (DMelSV) is a negative-sense RNA virus (family *Rhabdoviridae*) and a natural pathogen of *Drosophila melanogaster* that infects 0–20% of wild flies [30–32]. DMelSV is transmitted vertically through eggs and at a lower rate through sperm [30,33–37], and laboratory and field studies have estimated that infection results in a ~20% reduction in fitness [30,38,39] (Fig 1A). Incomplete vertical transmission, combined with the fitness costs of infection, prevents the virus from reaching fixation and instead maintains it at low prevalence. Although transmission through sperm occurs less efficiently than transmission through eggs, it is required to maintain the virus in the population [33].”

**Changes in manuscript, Methods (lines 815-825):**

*“Generation of infected lines.* Infected host–virus lines were established by pairing each of the nine host lines with each of the ten viral isolates. Viral isolates were introduced into DGRP host genetic backgrounds using a backcross scheme adapted from Wilfert & Jiggins (2010) [46], using the translocation balancer stock *T(2;3)*CyO-TM8 (CyO, *Adh*[nB]: TM8, *l(3)DTS4*[1], Bloomington). Balancer chromosomes carry dominant visible markers and suppress recombination, allowing specific chromosomes to be tracked through crosses. The translocation between chromosomes 2 and 3 makes the two main autosomes segregate in the progeny of the cross as if they were a single chromosome, simplifying the genetic crosses. We used the balancer stock to introduce each virus isolate into the intended DGRP genetic background in a controlled way and to confirm the resulting genotypes.”

**Changes in manuscript, Methods (lines 836-838):**

“Established stocks were maintained for two or more generations until infections were stabilised before phenotypic assays were performed. “

**Changes in manuscript, Methods (lines 869-870):**

“Infection was scored using the standard CO₂ assay, in which infected flies are paralysed by exposure to CO₂.”

**Changes in manuscript, Introduction:**

New figure:

**Suggested minor corrections:**

**a)	p.17 lines 352-353 (subtitle: Genetic interactions between host and virus reduce the effect of virulence on host fitness):** if a reduced effect of infection on host fitness is just due to all of the unexplained variation in host fitness (i.e., influences that are not accounted for here), then this inference and therefore subheading do not appear well founded; perhaps revise?

**Response:** We agree. Host and viral genetics together explained only 9% of the variance in hatch rate and 24% in fecundity, so most of the variation is not explained by the measured genetic effects. We have revised the subheading to refer specifically to the genetic component.

**Changes in manuscript, Results (lines 454-455):**

“Interactions between host and virus genotype reduce the additive genetic variance in host reproduction”

**Changes in manuscript, Results (lines 477-480):**

“Thus, host–virus genetic interactions are a detectable contributor to variation in the reproductive effects of infection, but they do not account for most of the observed phenotypic variation.”

**b)	Why just viral fitness p. 18 line 375?** **Why just host fitness p. 19 line 385?** These paragraphs are about fecundity and hatch rate, which are crucial to fitness of both partners. Perhaps revise to better integrate these descriptions?

**Response:** We agree, these traits matter to both partners. Because the virus is transmitted through the eggs, fecundity and hatch rate are fitness components for the host and the virus. We now state this once at the start of the section, and we no longer switch between "viral fitness" and "host fitness" in the results.

**Changes in manuscript, Results (lines 461-462):**

“As the virus is passed on through the eggs, these traits affect the fitness of both the host and the virus.”

**Changes in manuscript, Results (lines 481-483):**

“The main effect of viral genotype did not significantly influence hatch rate or fecundity (Fig 7D), indicating no detectable effect of viral genotype on host reproduction at the population level.”

**c)	p.21 line 434**: perhaps more accurate to say "mediators of fitness effects."

**Response:** We agree. This wording occurred in the opening summary of the experimental design, which has now been removed as part of shortening the Discussion. The potentially ambiguous description of viral load as a fitness measure has therefore also been removed.

**d)	p. 22 lines 458-459**: this interaction variance is only cryptic at the population scale! Seems worth acknowledging that you expect it to be very visible at individual scale. I do very much like the framing by the end of the paragraph, though!

**Response:** We thank the reviewer and agree. We now distinguish the effect of partner genotype on individual fitness from the population-scale result obtained after averaging across partner genotypes.

**Changes in manuscript, Discussion (lines 571-573):**

“Fitness depends on partner genotype but averaged across partners no host or virus genotype has a consistent advantage for directional selection to act on, so the interaction variance is cryptic only at the population scale.”

**e)	p. 23 lines 483-484**: the sentence beginning with "Moreover" was baffling. I thought you were already discussing effects of infection on fly fitness. Are you talking about some other trait? Did you forget to specify males here? Please rephrase for clarity?

**Response:** We apologise for the confusion. The original sentence did not clearly distinguish the female reproductive traits measured in our experiment from previous reports concerning infected males. We have rephrased it to identify both results explicitly.

**Changes in manuscript, Discussion (lines 650-656):**

“Intriguingly, despite well-documented fitness costs of sigma infection [33,34,39,68,86,87], the reproductive effect of infection was not uniform but depended on the particular host–virus combination and sometimes led to increases in female reproductive output, mirroring weakly supported reports of minor reproductive gains in infected males [66,88]. Whether these apparent increases represent reproducible biological responses to infection, and whether they occur in natural populations, remains unclear.”

**f)	p. 36 line 764**: not quite sure what you mean by "a factor defined by the pairwise combination" i.e., its arbitrarily assigned number or something? do you just mean a random interaction term for each pair?

**Response:** We thank the reviewer. We reworded the sentence for clarity. As the reviewer notes, the separate random interaction term for every host-virus pair provides the estimate of GH×GP interaction variance.

**Changes in manuscript, Methods (lines 966-968):**

“First, we fitted a univariate model to each of the six traits, with no fixed effects specified and host identity, virus identity, and their interaction fitted as random effects.”
