# Supplementary material for: Host–virus interactions dominate fitness and conceal additive genetic variation
Source: PLoS Biol. 2026 Oct 22;24(10):e3003836. doi: 10.1371/journal.pbio.3003836 (PMC13690250; doi:10.1371/journal.pbio.3003836)
Supplement: Peer review history

# Editor report: Decision Letter 0

**DOI:** 10.1371/journal.pbio.3003836.r001

**Authors:** Roland Roberts

18 May 2026

Dear Dr Belyi,

Thank you for submitting your manuscript entitled "Drosophila–virus genotype interactions dominate transmission, virulence and load, eroding additive fitness variance" for consideration as a Research Article by PLOS Biology.

Your manuscript has now been evaluated by the PLOS Biology editorial staff [as well as by an academic editor with relevant expertise, and I'm writing to let you know that we would like to send your submission out for external peer review.

However, before we can send your manuscript to reviewers, we need you to complete your submission by providing the metadata that is required for full assessment. To this end, please login to Editorial Manager where you will find the paper in the 'Submissions Needing Revisions' folder on your homepage. Please click 'Revise Submission' from the Action Links and complete all additional questions in the submission questionnaire.

Once your full submission is complete, your paper will undergo a series of checks in preparation for peer review. After your manuscript has passed the checks it will be sent out for review. To provide the metadata for your submission, please Login to Editorial Manager (https://www.editorialmanager.com/pbiology) within two working days, i.e. by May 20 2026 11:59PM.

If your manuscript has been previously peer-reviewed at another journal, PLOS Biology is willing to work with those reviews in order to avoid re-starting the process. Submission of the previous reviews is entirely optional and our ability to use them effectively will depend on the willingness of the previous journal to confirm the content of the reports and share the reviewer identities. Please note that we reserve the right to invite additional reviewers if we consider that additional/independent reviewers are needed, although we aim to avoid this as far as possible. In our experience, working with previous reviews does save time.

If you would like us to consider previous reviewer reports, please edit your cover letter to let us know and include the name of the journal where the work was previously considered and the manuscript ID it was given. In addition, please upload a response to the reviews as a 'Prior Peer Review' file type, which should include the reports in full and a point-by-point reply detailing how you have or plan to address the reviewers' concerns.

During the process of completing your manuscript submission, you will be invited to opt-in to posting your pre-review manuscript as a bioRxiv preprint. Visit http://journals.plos.org/plosbiology/s/preprints for full details. If you consent to posting your current manuscript as a preprint, please upload a single Preprint PDF.

Feel free to email us at plosbiology@plos.org if you have any queries relating to your submission.

Kind regards,

Roli Roberts

Roland Roberts, PhD

Senior Editor

PLOS Biology

rroberts@plos.org

# Peer review: Decision Letter 1

**DOI:** 10.1371/journal.pbio.3003836.r002

**Authors:** Roland Roberts

1 Jul 2026

Dear Dr Belyi,

Thank you for your patience while your manuscript "Drosophila–virus genotype interactions dominate transmission, virulence and load, eroding additive fitness variance" was peer-reviewed at PLOS Biology. It has now been evaluated by the PLOS Biology editors, an Academic Editor with relevant expertise, and by four independent reviewers.

You'll see that reviewer #1 is positive, but wants you to improve accessibility, fix a discrepancy in Fig 5, wonders about the consequences of geographically mismatched virus and host populations, asks about including a clearance term in your model, and wants you to discuss generalisability. Reviewer #2 is also positive, but is concerned about the impact of the virus/host mismatch (and the greater diversity of the former), s/he also spots the issue with Fig 5 and has a number of other requests for clarification. Reviewer #3 is again positive, but wants you to tone down some conclusions and tighten up the Discussion. Reviewer #4 is very positive, but his/her four major points all concern issues of clarity and accessibility, especially for a broader readership (including a Fig showing the experimental design).

Based on the reviews, we are likely to accept this manuscript for publication, provided you satisfactorily address the points raised by the reviewers. Please also make sure to address the following data and other policy-related requests.

IMPORTANT - please attend to the following:

a) Please address the comments from the reviewers.

b) We're finding the current Title a little hard to parse. Please could you consider some more explicit versions - we suggest "Host–virus genotype interactions dominate transmission, virulence and load during infection of Drosophila by sigma virus" or "Host–virus genotype interactions erode additive fitness variance by dominating transmission, virulence and load" (my personal preference would be the latter).

c) Please address my Data Policy requests below; specifically, we need you to supply the numerical values underlying Figs 1ABCDEF, 2ABC, 3ABCDE, 4ABCDE, 5AB, 6ABCD, 7AB, S2ABCDEF, S3ABCDEF, S4ABC, S5ABCD, S6ABCDEFGHIJK, S7ABCDEFGHIJK, either as a supplementary data file or as a permanent DOI’d deposition.

d) Please cite the location of the data clearly in all relevant main and supplementary Figure legends, e.g. “The data underlying this Figure can be found in S1 Data” or “The data underlying this Figure can be found in https://zenodo.org/records/XXXXXXXX; ; also update your Data Availability Statement accordingly.

e) Please make any custom code available, either as a supplementary file or as part of your data deposition.

As you address these items, please take this last chance to review your reference list to ensure that it is complete and correct. If you have cited papers that have been retracted, please include the rationale for doing so in the manuscript text, or remove these references and replace them with relevant current references. Any changes to the reference list should be mentioned in the cover letter that accompanies your revised manuscript.

In addition to these revisions, you may need to complete some formatting changes, which you will receive in a follow up email. A member of our team will be in touch with a set of requests shortly. If you do not receive a separate email within a few days, please assume that checks have been completed, and no additional changes are required.

We expect to receive your revised manuscript within three weeks.

To submit your revision, please go to https://www.editorialmanager.com/pbiology/ and log in as an Author. Click the link labelled 'Submissions Needing Revision' to find your submission record. Your revised submission must include the following:

- a cover letter that should detail your responses to any editorial requests, if applicable, and whether changes have been made to the reference list

- a Response to Reviewers file that provides a detailed response to the reviewers' comments (if applicable, if not applicable please do not delete your existing 'Response to Reviewers' file.)

- a track-changes file indicating any changes that you have made to the manuscript.

NOTE: If Supporting Information files are included with your article, note that these are not copyedited and will be published as they are submitted. Please ensure that these files are legible and of high quality (at least 300 dpi) in an easily accessible file format. For this reason, please be aware that any references listed in an SI file will not be indexed. For more information, see our Supporting Information guidelines:

https://journals.plos.org/plosbiology/s/supporting-information

*Published Peer Review History*

Please note that you may have the opportunity to make the peer review history publicly available. The record will include editor decision letters (with reviews) and your responses to reviewer comments. If eligible, we will contact you to opt in or out. Please see here for more details:

https://plos.org/published-peer-review-history/

*Press*

Should you, your institution's press office or the journal office choose to press release your paper, please ensure you have opted out of Early Article Posting on the submission form. We ask that you notify us as soon as possible if you or your institution is planning to press release the article.

*Protocols deposition*

To enhance the reproducibility of your results, we recommend that if applicable you deposit your laboratory protocols in protocols.io, where a protocol can be assigned its own identifier (DOI) such that it can be cited independently in the future. Additionally, PLOS ONE offers an option for publishing peer-reviewed Lab Protocol articles, which describe protocols hosted on protocols.io. Read more information on sharing protocols at https://plos.org/protocols?utm_medium=editorial-email&utm_source=authorletters&utm_campaign=protocols

Please do not hesitate to contact me should you have any questions.

Sincerely,

Roli Roberts

Roland Roberts, PhD

Senior Editor

rroberts@plos.org

PLOS Biology

------------------------------------------------------------------------

DATA POLICY:

You may be aware of the PLOS Data Policy, which requires that all data be made available without restriction: http://journals.plos.org/plosbiology/s/data-availability. For more information, please also see this editorial: http://dx.doi.org/10.1371/journal.pbio.1001797

Note that we do not require all raw data. Rather, we ask that all individual quantitative observations that underlie the data summarized in the figures and results of your paper be made available in one of the following forms:

1) Supplementary files (e.g., excel). Please ensure that all data files are uploaded as 'Supporting Information' and are invariably referred to (in the manuscript, figure legends, and the Description field when uploading your files) using the following format verbatim: S1 Data, S2 Data, etc. Multiple panels of a single or even several figures can be included as multiple sheets in one excel file that is saved using exactly the following convention: S1_Data.xlsx (using an underscore).

2) Deposition in a publicly available repository. Please also provide the accession code or a reviewer link so that we may view your data before publication.

Regardless of the method selected, please ensure that you provide the individual numerical values that underlie the summary data displayed in the following figure panels as they are essential for readers to assess your analysis and to reproduce it: Figs 1ABCDEF, 2ABC, 3ABCDE, 4ABCDE, 5AB, 6ABCD, 7AB, S2ABCDEF, S3ABCDEF, S4ABC, S5ABCD, S6ABCDEFGHIJK, S7ABCDEFGHIJK. NOTE: the numerical data provided should include all replicates AND the way in which the plotted mean and errors were derived (it should not present only the mean/average values).

IMPORTANT: Please also ensure that figure legends in your manuscript include information on where the underlying data can be found, and ensure your supplemental data file/s has a legend.

Please ensure that your Data Statement in the submission system accurately describes where your data can be found.

------------------------------------------------------------------------

CODE POLICY

Per journal policy, if you have generated any custom code during the course of this investigation, please make it available without restrictions. Please ensure that the code is sufficiently well documented and reusable, and that your Data Statement in the Editorial Manager submission system accurately describes where your code can be found. More information on our Code Policy, what and how to share can be found here: https://journals.plos.org/plosbiology/s/code-availability

Please note that we cannot accept sole deposition of code in GitHub, as this could be changed after publication. However, you can archive this version of your publicly available GitHub code to Zenodo. Once you do this, it will generate a DOI number, which you will need to provide in the Data Accessibility Statement (you are welcome to also provide the GitHub access information). See the process for doing this here: https://docs.github.com/en/repositories/archiving-a-github-repository/referencing-and-citing-content

------------------------------------------------------------------------

DATA NOT SHOWN?

- Please note that per journal policy, we do not allow the mention of "data not shown", "personal communication", "manuscript in preparation" or other references to data that is not publicly available or contained within this manuscript. Please either remove mention of these data or provide figures presenting the results and the data underlying the figure(s).

------------------------------------------------------------------------

REVIEWERS' COMMENTS:

Reviewer #1:

This manuscript presents a methodologically ambitious and mathematically rigorous investigation into host-pathogen coevolution. The extensive scale of the experiment and the subsequent application of quantitative genetic models provide a compelling framework for understanding the maintenance of genetic variation. We congratulate the authors on the execution of an experimental undertaking of this magnitude. To ensure the manuscript's conclusions are robustly supported, we suggest the following revisions.

1) Considering the broad readership of PLoS Biology, the manuscript would benefit from a more accessible conceptual foundation early in the text. While the manuscript is clearly written, it assumes a high level of specialized prior knowledge regarding quantitative genetics and the decomposition of fitness variance. We recommend including a short paragraph early in the introduction that conceptually defines the genotype-by-genotype interactions and explains why distinguishing between additive and non-additive fitness variance is a critical piece of the "big picture" in evolutionary biology. This will help readers to understand and value the work.

2) There is a discrepancy between the text and the data presented in Figure 5 that must be resolved. Lines 329-331 state that transmission is positively correlated with viral load, reporting positive Pearson correlation coefficient. However, the regression lines plotted in both panels of Figure 5 exhibit clear negative slopes, indicating that higher viral loads correlate with decreased transmission.

3) The host genetic variance is calculated from a non-random subset of nine DGRP lines, which were deliberately chosen to represent specific combinations of major-effect resistance alleles. The authors must address whether this stratification artificially inflates the perceived genetic and interaction variances compared to what would be observed in a naturally segregating, panmictic population. Furthermore, pairing a localized North American fly population with geographically diverse viral isolates introduces artificial allopatry. The resulting high GH×GP variance may reflect the breakdown of local adaptation rather than the equilibrium state of a sympatric coevolving population. The authors should explicitly discuss these limitations and how sympatric dynamics might alter the ratio of main effects to interaction effects.

4) The mathematical model detailed in Figure 8 contains a rigid biological assumption regarding the developmental trajectory of the virus. The model dictates that all surviving infected zygotes inevitably develop into infected adults, subject only to the viability cost parameter c. This formulation assumes a zero percent viral clearance rate during host development. The authors should clarify whether complete viral persistence through metamorphosis occurs for the sigma virus. If the host can clear the infection during larval stages, the authors should consider incorporating a clearance parameter into the model, or at least discuss how virus clearance might alter the predicted coevolutionary oscillations.

5) We strongly recommend expanding the Discussion to address whether these findings can be extrapolated to horizontally transmitted viruses.

Reviewer #2:

This study investigates the effects of host-pathogen coevolution by negative frequency-dependent selection (NFDS) on genetic variation in both host and pathogen. Specifically, the authors estimate what fraction of the genetic variation in virulence and transmission that is heritable vs bound in host genotype-by-pathogen genotype interactions (G*G). Interestingly, the authors found that most of the variation is in the G*G variance component. They also found strong G*G*sex interactions which is intriguing; cannot think of any previous studies that have shown that. The authors use a novel approach to a question that is of broad interest. The paper is very well written and the presentation is clear.

The only major issue I can see concerns the sampling design of host and, especially, pathogen genotypes. NFDS is a process that occurs within populations. To estimate the consequences of NFDS (as regards e.g. variance components) one should therefore have random samples of host and pathogen genotypes from a given population at a given point in time. Here, the authors have rather tried to maximize the genetic diversity, especially for the virus where samples are from both Europe and North America (and no isolate seems to be from where the fly lines were originally collected). Thus, the pathogen in particular is likely much more genetically diverse in this study than what is the case in a natural population. Also, it is not matched to the host population. I suspect that this might inflate the G*G variance component. I understand that it would be a daunting task to collect both fly and virus lines from a given population, but this limitation of the study should still be acknowledged and discussed (the authors touch on this on L 460-467 but I think the potential problem with the sampling design needs to be made more clear).

Other relatively minor comments/questions:

L 23-24 & L 406-407: Is it NFDS that leads to G*G (as implied by wording on L 23-24) or G*G that leads to NFDS (as implied by wording on L 406-407)? I would say the latter, but guess it depends on whether you think of G*G "mechanistically" (where G*G->NFDS) or the G*G variance component (in which case NFDS drives G*G variance). In any case, I think it would be good to use a consistent perspective throughout the ms.

L 34: With the experimental design used here (inbred lines), I believe the main effect of host estimates the broad-sense heritability, i.e. not only additive but also dominance and epistasis, right? And I think it would be same for the virus. If so, this should be clarified throughout.

L 58: "pathogens targeted by that [host] allele" implies infection matrix of the Gene-For-Gene type (or inverse Matching Allele), while in Matching Allele it is the pathogen that targets the host. Since your analysis is agnostic to whether the G*G is of GFG or MA type, I suggest using another word than "target" that is neutral to the direction of the interaction.

L 131-132: The minor allele frequency of the host polymorphism is surprisingly low (<0.1) for a balanced polymorphism. It seems unrealisitc that this can maintained in the long term in the face of drift, or? Perhaps worth a comment and/or comparison with previous similar models.

L 154-155 & fig 1: I think it would be good to include a fig specifying the infection matrix and explain if this corresponds to GFG or MA (or perhaps inverse GFG/MA).

L 329-331. Explain how the negative slopes in fig 5 correspond to positive correlations between transmission and viral load.

L 378-379: It is not clear to me why this would reflect tolerance rather than resistance. As you did not include load as a covariate you cannot separate resistance and tolerance, or am I missing something?

Reviewer #3:

General comment: The integrated approach combining simulations and experiments to investigating negative frequency-dependent selection (NFDS) makes this study quite interesting for the host/viral interactions field. The authors first established explicit theoretical expectations using coevolutionary models and then test these predictions experimentally across a large host-virus factorial design. The results and inferences derived from the D. melanogaster-Sigma virus system are likely to provide a useful framework for investigating genotype-by-genotype interactions and coevolutionary dynamics in other host-virus systems, generating testable hypotheses that can be evaluated across a broader range of biological models.

The authors appropriately acknowledge that many genetic correlation estimates are associated with large standard errors and should be interpreted cautiously (lines 336-337 and 501-504). Nevertheless, some sections of the Results still draw biological conclusions from rather imprecise estimates. For example, the discussion of sex-specific transmission architecture (lines 253-261) interprets host and virus cross-sex genetic correlations despite substantial uncertainty around these estimates (host: rg = 0.19 ± 0.46; virus: rg = 0.57 ± 0.39). Similarly, the conclusions regarding shared genetic control of viral replication between sexes (lines 303-308) are based on a host genetic correlation estimated as rg = 0.71 ± 0.39. Finally, the interpretation of viral load-transmission relationships (lines 336-343) relies on several genetic correlations with large standard errors (e.g., host effect in males: rg = 0.52 ± 0.42; host effect in females: rg = 0.46 ± 0.34). I suggest the authors to consider further softening these interpretations.

750-751: "A large sex-specific difference in RpL32 expression was detected, requiring sex- specific normalization"

Is it expected for D. melanogaster? Another gene could have provided better normalization for this experimental design?

The Discussion is quite long and often repeat the results. While the authors appropriately focus on the Drosophila-sigma virus system, the Discussion could more explicitly acknowledge the extent to which the observed patterns may depend on the biology of sigma virus itself. Sigma virus is a vertically transmitted, host-specialized negative-sense RNA virus, and its coevolutionary dynamics may differ substantially from those of horizontally transmitted viruses, segmented RNA viruses capable of reassortment, or large DNA viruses with different evolutionary constraints. A brief discussion of the potential limits to generalizing the observed dominance of GH×GP interactions across other host-virus systems would also strengthen the manuscript.

Reviewer #4:

This manuscript describes an ambitious and rigorous study that combines theory and experiment to address important questions in the biology of host-parasite coevolution. The results are fascinating and I learned a great deal from reading the manuscript. I expect that this work will ultimately become a classic of coevolutionary biology.

My absolute favorite bits include the rigor and clarity of the evidence of GxG for transmission, as well as the compelling evidence for sex specificity in many of the genetic and GxG effects. In general, I find the empirical results considerably more interesting and compelling than the theory but can see that the theory may fill a gap in the literature on vertically transmitted parasites while also explicitly tracking GxG variance.

I have several suggestions to improve the clarity and impact of the manuscript, as follows.

Suggestions for revision:

1)Clarify/broaden the framing to acknowledge the diversity of modes of selection.

In many places, the narrow evolutionary framing was jarring. For ex, the sentence about an "evolutionary response" (p. 6 lines 118-119) implies that balancing selection is not evolution. Perhaps instead specify directional selection here? Similarly, on p. 25 lines 527-528: "coevolution masking heritable variation from the effects of natural selection" could equally be framed as "coevolution dynamically maintaining heritable variation via natural selection." I take your point that net fitness advantages of a given G disappear, even if there are transient advantages. But I don't see the need for an exclusive focus on directional selection so suggest also acknowledging diversity-promoting balancing selection.

2)Explain figures more fully and clearly. The manuscript includes many figures of considerable complexity and in several cases, the explanations were insufficient for me. I was especially baffled by the apparent disconnect between the description of figure 5 in the text and the appearance of both panels: the relationships do not look positive, and indeed, if correlated at all, the data suggest a negative relationship (e.g., a cost of virulence?). Please check and clarify? perhaps phenotypic correlations obscure the direction of the genetic correlations? Or did I miss a key point here? I also think that Fig 2 needs more explanation, especially of the curved region (surrounding the diagonal cost line) that is associated with higher CV and AGV. Does that range of cost balances somehow alter GxG impacts?

3)Deepen discussion of all results, whether or not they align with the GxG-centred framing. The hard-won results seem under-discussed in various ways. If space permits, I suggest some deepening of the Discussion section. For ex, inspired by p. 11 lines 217-219: this is totally fascinating and seems a promising area for future study. Perhaps elaborate on how to investigate potentially sex-specific mechanisms and what we might learn from such studies? I also think it is important for the authors to better probe the relative lack of GxG for fly fecundity and hatch rate: For ex, on p. 22 lines 447-450, this inference doesn't sit quite right, in part because "dominates" doesn't seem right if only ~6-10% of variance is explained, and if host main effects can rival the explanatory power of GxG for fecundity. What unmanipulated, unmeasured traits in this study influence fecundity and hatch rate? The Discussion needs to better capture these interesting nuances. The message of GxG impacts still will come across! Finally, you mention the possibility of tolerance as an explanation in the results but don't go there in the Discussion - please do?

4)Explain the system better for a wide audience who will largely be unfamiliar with it. For a generalist audience, a bit more explanation of the system in the main text might help outsiders understand the biology as well as your methods. For ex, why are the viruses at such low prevalence, if solely vertically transmitted? Is it because they tend to be virulent (in other studies, if not this one)? In the Methods: why do you need a "balancer" stock? might your requirement for "stable transmission rates" (p. 32 lines 672-673) have made it inevitable that you would not observe selective sweeps? A figure to illustrate the complexities of the line establishment and experimental design might also be useful to convey system info and thus strengthen reader confidence in the approaches and findings.

Suggested minor corrections:

a)p.17 lines 352-353: if a reduced effect of infection on host fitness is just due to all of the unexplained variation in host fitness (i.e., influences that are not accounted for here), then this inference and therefore subheading do not appear well founded; perhaps revise?

b)Why just viral fitness p. 18 line 375? Why just host fitness p. 19 line 385? These paragraphs are about fecundity and hatch rate, which are crucial to fitness of both partners. Perhaps revise to better integrate these descriptions?

c)p.21 line 434: perhaps more accurate to say "mediators of fitness effects."

d)p. 22 lines 458-459: this interaction variance is only cryptic at the population scale! Seems worth acknowledging that you expect it to be very visible at individual scale. I do very much like the framing by the end of the paragraph, though!

e)p. 23 lines 483-484: the sentence beginning with "Moreover" was baffling. I thought you were already discussing effects of infection on fly fitness. Are you talking about some other trait? Did you forget to specify males here? Please rephrase for clarity?

f)p. 36 line 764: not quite sure what you mean by "a factor defined by the pairwise combination" i.e., its arbitrarily assigned number or something? do you just mean a random interaction term for each pair?

# Author comment: Author response to Decision Letter 2

**DOI:** 10.1371/journal.pbio.3003836.r003

18 Aug 2026

Attachment Submitted filename: Response_to_Reviewers.docx

# Editor report: Decision Letter 2

**DOI:** 10.1371/journal.pbio.3003836.r004

**Authors:** Roland Roberts

20 Aug 2026

Dear Aleksei,

Thank you for the submission of your revised Research Article "Host–virus interactions dominate fitness and conceal additive genetic variation" for publication in PLOS Biology. On behalf of my colleagues and the Academic Editor, Harmit Malik, I'm pleased to say that we can in principle accept your manuscript for publication, provided you address any remaining formatting and reporting issues. These will be detailed in an email you should receive within 2-3 business days from our colleagues in the journal operations team; no action is required from you until then. Please note that we will not be able to formally accept your manuscript and schedule it for publication until you have completed any requested changes.

IMPORTANT: I'm aware that I had previously suggested a different Title, which you then adopted. However, the Academic Editor strongly suggested that it be changed to something "pithier and more impactful," and suggested "Host–virus interactions dominate fitness and conceal additive genetic variation." I've taken the liberty of changing the title to this, but please let me know if this presents any problems.

Please take a minute to log into Editorial Manager at http://www.editorialmanager.com/pbiology/, click the "Update My Information" link at the top of the page, and update your user information to ensure an efficient production process.

PRESS: We frequently collaborate with press offices. If your institution or institutions have a press office, please notify them about your upcoming paper at this point, to enable them to help maximise its impact. If the press office is planning to promote your findings, we would be grateful if they could coordinate with biologypress@plos.org. If you have previously opted in to the early version process, we ask that you notify us immediately of any press plans so that we may opt out on your behalf.

We also ask that you take this opportunity to read our Embargo Policy regarding the discussion, promotion and media coverage of work that is yet to be published by PLOS. As your manuscript is not yet published, it is bound by the conditions of our Embargo Policy. Please be aware that this policy is in place both to ensure that any press coverage of your article is fully substantiated and to provide a direct link between such coverage and the published work. For full details of our Embargo Policy, please visit http://www.plos.org/about/media-inquiries/embargo-policy/.

Thank you again for choosing PLOS Biology for publication and supporting Open Access publishing. We look forward to publishing your study.

Sincerely,

Roli Roberts

Roland G Roberts, PhD, PhD

Senior Editor

PLOS Biology

rroberts@plos.org
